# Supplementary material for: Monthly sulfadoxine/pyrimethamine-amodiaquine or dihydroartemisinin-piperaquine as malaria chemoprevention in young Kenyan children with sickle cell anemia: A randomized controlled trial
Source: PLoS Med. 2022 Oct 10;19(10):e1004104. doi: 10.1371/journal.pmed.1004104 (PMC9591057; doi:10.1371/journal.pmed.1004104)
Supplement: S1 Protocol — (DOCX) [file pmed.1004104.s002.docx]

**Enhancing Preventive Therapy of Malaria In children with Sickle cell anemia in East Africa (EPiTOMISE)**

A randomized trial of daily Proguanil, monthly Sulfadoxine/Pyrimethamine-Amodiaquine, or monthly Dihydroartemsinin-Piperaquine for the prevention of malaria in children with sickle cell anemia in Homa Bay, Kenya

**Protocol Identifying Number: 1R01HL134211**

**Principal Investigators:** **Festus Njuguna MBChB MMed PhD (Pediatrics)**

Moi University School of Medicine

**Steve M Taylor MD MPH**

Duke Clinical Research Institute

Duke University School of Medicine

**Co-Investigator: Wendy P O’Meara PhD**

Duke Global Health Institute

**Funded by: The U.S. National Institutes of Health/ National Heart, Lung and Blood Institute (NIH/NHLBI)**

**Version Number: v10.0**

**April 14, 2020**

Table of Contents

[LIST OF ABBREVIATIONS 5](#_Toc523141801)

[STATEMENT OF COMPLIANCE 7](#_Toc523141802)

[PROTOCOL SUMMARY 9](#_Toc523141803)

[SCHEMATIC OF STUDY DESIGN 10](#_Toc523141804)

[AMENDMENT HISTORY 11](#_Toc523141805)

[1 INTRODUCTION: BACKGROUND & SCIENTIFIC RATIONALE 12](#_Toc523141806)

[1.1 Background Information 12](#_Toc523141807)

[1.1.1 Epidemiology of sickle cell anemia and malaria in Africa 12](#_Toc523141808)

[1.1.2 Chemoprevention of malaria in high-risk groups 13](#_Toc523141809)

[1.1.3 Study site 14](#_Toc523141810)

[1.1.4 Potential efficacy of chemoprevention agents 15](#_Toc523141811)

[1.2 Rationale 16](#_Toc523141812)

[1.3 Potential Risks and Benefits 17](#_Toc523141813)

[1.3.1 Known potential risks 17](#_Toc523141814)

[1.3.2 Known potential benefits 18](#_Toc523141815)

[2 OBJECTIVES AND PURPOSE 19](#_Toc523141816)

[3 STUDY DESIGN AND ENDPOINTS 20](#_Toc523141817)

[3.1 Description of the Study Design 20](#_Toc523141818)

[3.2 Primary Endpoint 20](#_Toc523141819)

[3.3 Secondary Endpoints 20](#_Toc523141820)

[4 STUDY ENROLLMENT AND WITHDRAWAL 23](#_Toc523141821)

[4.1 Participant Inclusion Criteria 23](#_Toc523141822)

[4.2 Participant Exclusion Criteria 23](#_Toc523141823)

[4.3 Strategies for Recruitment and Retention 23](#_Toc523141824)

[4.4 Participant Withdrawal or Termination 24](#_Toc523141825)

[4.4.1 Reasons for withdrawal or termination 24](#_Toc523141826)

[4.4.2 Handling of participant withdrawals or termination 24](#_Toc523141827)

[4.5 Premature Termination or Suspension of Study 25](#_Toc523141828)

[5 STUDY AGENT 26](#_Toc523141829)

[5.1 Study Agents and Control Description 26](#_Toc523141830)

[5.1.1 Acquisition 26](#_Toc523141831)

[5.1.2 Formulation, appearance, packaging, and labeling 26](#_Toc523141832)

[5.1.3 Product storage and stability 27](#_Toc523141833)

[5.1.4 Dosing and administration 27](#_Toc523141834)

[5.1.5 Route of administration 28](#_Toc523141835)

[5.1.6 Duration of therapy 28](#_Toc523141836)

[5.1.7 Tracking of dose 28](#_Toc523141837)

[5.2 Study Agent Accountability Procedures 28](#_Toc523141838)

[6 STUDY PROCEDURES AND SCHEDULE 29](#_Toc523141839)

[6.1 Study Procedures/Evaluations 29](#_Toc523141840)

[6.1.1 Study specific procedures 29](#_Toc523141841)

[6.1.2 Standard of care study procedures 29](#_Toc523141842)

[6.2 Laboratory Procedures/Evaluations 30](#_Toc523141843)

[6.2.1 Clinical laboratory evaluations 30](#_Toc523141844)

[6.2.2 Other assays or procedures 30](#_Toc523141845)

[6.2.3 Specimen preparation, handling, and storage 31](#_Toc523141846)

[6.2.4 Specimen shipment 31](#_Toc523141847)

[6.3 Study Schedule 31](#_Toc523141848)

[6.3.1 General screening procedures 31](#_Toc523141849)

[6.3.2 Enrollment 32](#_Toc523141850)

[6.3.3 Routine follow-up visits 33](#_Toc523141851)

[6.3.4 Final study visit 34](#_Toc523141852)

[6.3.5 Early termination visit 34](#_Toc523141853)

[6.3.6 Acute-care visits through month 12 34](#_Toc523141854)

[6.3.7 Schedule of events table through month 12 35](#_Toc523141855)

[6.4 Concomitant Medications, Treatments, and Procedures 35](#_Toc523141856)

[6.4.1 Precautionary medications, treatments, and procedures 36](#_Toc523141857)

[6.5 Prohibited Medications, Treatments, and Procedures 36](#_Toc523141858)

[6.6 Rescue Medications, Treatments, and Procedures 36](#_Toc523141859)

[6.7 Participant Access to Study Agent at Study Closure 36](#_Toc523141860)

[7 ASSESSMENT OF SAFETY 37](#_Toc523141861)

[7.1 Specification of Safety Parameters 37](#_Toc523141862)

[7.1.1 Definition of Suspected Unexpected Serious Adverse Reaction (SUSAR) 37](#_Toc523141863)

[7.1.2 Ascertainment of SUSARs 37](#_Toc523141864)

[7.1.3 Time Period and Frequency for Event Assessment and Follow-Up 38](#_Toc523141865)

[7.2 Reporting Procedures 38](#_Toc523141866)

[7.2.1 Reporting of SUSARs 38](#_Toc523141867)

[7.2.2 Reporting of Participant Deaths 38](#_Toc523141868)

[7.2.3 Reporting of Unanticipated Problems 38](#_Toc523141869)

[7.2.4 Summary of Event Reporting 38](#_Toc523141870)

[7.3 Study Halting Rules 38](#_Toc523141871)

[7.4 Safety Oversight 39](#_Toc523141872)

[8 CLINICAL MONITORING 40](#_Toc523141873)

[9 STATISTICAL CONSIDERATIONS 41](#_Toc523141874)

[9.1 Statistical and Analytical Plans 41](#_Toc523141875)

[9.2 Statistical Hypotheses 41](#_Toc523141876)

[9.3 Analysis Datasets 41](#_Toc523141877)

[9.4 Description of Statistical Methods 42](#_Toc523141878)

[9.4.1 General approach 42](#_Toc523141879)

[9.4.2 Analysis of the primary efficacy endpoint 42](#_Toc523141880)

[9.4.3 Analysis of the secondary endpoints 43](#_Toc523141881)

[9.4.4 Data and safety analyses 43](#_Toc523141882)

[9.4.5 Adherence and retention analyses 44](#_Toc523141883)

[9.4.6 Baseline descriptive statistics 44](#_Toc523141884)

[9.4.7 Planned interim analyses 44](#_Toc523141885)

[9.4.8 Additional sub-group analyses 45](#_Toc523141886)

[9.4.9 Tabulation of individual response data 45](#_Toc523141887)

[9.4.10 Exploratory analyses 45](#_Toc523141888)

[9.5 Sample Size 45](#_Toc523141889)

[9.6 Measures to Minimize Bias 47](#_Toc523141890)

[9.6.1 Enrollment/Randomization 47](#_Toc523141891)

[10 SOURCE DOCUMENTS AND ACCESS TO SOURCE DATA/DOCUMENTS 48](#_Toc523141892)

[11 QUALITY ASSURANCE AND QUALITY CONTROL 49](#_Toc523141893)

[12 ETHICS/PROTECTION OF HUMAN SUBJECTS 50](#_Toc523141894)

[12.1 Ethical Standard 50](#_Toc523141895)

[12.2 Institutional Review Board 50](#_Toc523141896)

[12.3 Informed Consent Process 50](#_Toc523141897)

[12.3.1 Consent and other informational documents provided to participants 50](#_Toc523141898)

[12.3.2 Consent procedures and documentation 51](#_Toc523141899)

[12.4 Participant and Data Confidentiality 51](#_Toc523141900)

[12.4.1 Research use of stored human samples, specimens or data 52](#_Toc523141901)

[12.5 Potential Future Use of Stored Specimens 52](#_Toc523141902)

[13 DATA HANDLING AND RECORD KEEPING 54](#_Toc523141903)

[13.1 Data Collection and Management Responsibilities 54](#_Toc523141904)

[13.2 Study Records Retention 54](#_Toc523141905)

[13.3 Protocol Deviations 54](#_Toc523141906)

[13.4 Publication and Data Sharing Policy 54](#_Toc523141907)

[13.5 Data and Safety Monitoring Plan 55](#_Toc523141908)

[14 STUDY ADMINISTRATION 56](#_Toc523141909)

[14.1 Coordinating Center 56](#_Toc523141910)

[15 CONFLICT OF INTEREST POLICY 57](#_Toc523141911)

[16 LITERATURE REFERENCES 58](#_Toc523141912)

[APPENDIX A: STUDY INFORMATION SHEET (ENGLISH) 62](#_Toc523141913)

[APPENDIX B: STUDY INFORMATION SHEET (KISWAHILI) 63](#_Toc523141914)

[APPENDIX C: STUDY INFORMATION SHEET (KISWAHILI TO ENGLISH) 64](#_Toc523141915)

[APPENDIX D: STUDY INFORMATION SHEET (DHOLUO) 65](#_Toc523141916)

[APPENDIX E: STUDY INFORMATION SHEET (DHOLUO TO ENGLISH) 66](#_Toc523141917)

[APPENDIX F: INFORMED CONSENT (ENGLISH) 67](#_Toc523141918)

[APPENDIX G: INFORMED CONSENT (KISWAHILI) 78](#_Toc523141919)

[APPENDIX H: INFORMED CONSENT (KISWAHILI TO ENGLISH) 90](#_Toc523141920)

[APPENDIX J: INFORMED CONSENT (DHOLUO) 101](#_Toc523141921)

[APPENDIX K: INFORMED CONSENT (DHOLUO TO ENGLISH) 111](#_Toc523141922)

[APPENDIX L: MEDICATIONS THAT PROLONG THE QT INTERVAL 122](#_Toc523141923)

#

## LIST OF ABBREVIATIONS

| ACT | Artemisinin-combination therapy |
| --- | --- |
| ACS | Acute chest syndrome |
| AE | Adverse event |
| AL | Artemether-lumefantrine |
| ALT | Alanine aminotransferase |
| AMPATH | Academic Model Providing Access to Healthcare |
| AP | Atovaquone-proguanil |
| AT | As-treated |
| ATP | According-to-protocol |
| BABY-HUG | Pediatric hydroxyurea Phase 3 clinical trial (NCT00006400) |
| BID | Bis in die (twice a day) |
| BUN | Blood urea nitrogen |
| CBC | Complete blood count |
| CC | Coordinating center |
| CFR | Code of Federal Regulations (United States) |
| CQ | Chloroquine |
| DBS | Dried blood spot |
| DCRI | Duke Clinical Research Institute |
| DP | Dihydroartemisinin-piperaquine |
| DSMB | Data and Safety Monitoring Board |
| DSMP | Data and Safety Monitoring Plan |
| DUMC | Duke University Medical Center |
| eCRF | Electronic case report form |
| ECG | Electrocardiogram |
| EDC | Electronic data capture |
| EPiTOMISE | Enhancing Preventive Therapy of Malaria In children with Sickle cell anemia in East Africa |
| FDA | Food and Drug Administration (United States) |
| GCP | Good clinical practice |
| GLP | Good laboratory practice |
| GMP | Good manufacturing practice |
| H_O_ | Null hypothesis |
| H_A_ | Alternative hypothesis |
| HBCH | Homa Bay County Hospital |
| HbF | Hemoglobin F |
| HbSS | Hemoglobin SS |
| HIV | Human Immunodeficiency Virus |
| HPLC | High Performance Liquid Chromatography |
| IATA | International Air Transportation Association |
| ICF | Informed consent form |
| ICH | International Council for Harmonisation of Technical Requirements for Pharmaceuticals for Human Use |
| ICMJE | International Council of Medical Journal Editors |
| IPT | Intermittent preventative therapy |
| IR | Incidence rate |
| IRB | Institutional Review Board |
| IREC | Institutional Research Ethics Committee (Moi University) |
| ITN | Insecticide-treated bednet |
| ITT | Intention-to-treat |
| LAR | Legally Authorized Representative |
| LM | Light microscopy |
| MOP | Manual of procedures |
| MTRH | Moi Teaching and Referral Hospital |
| MQ | Mefloquine |
| MQAS | Mefloquine-artesunate |
| MSM | Medical Safety Monitor |
| NHIF | National Hospital Insurance Fund (Kenya) |
| NHLBI | National Heart, Lung, and Blood Institute (United States) |
| NIH | National Institutes of Health (United States) |
| PCR | Polymerase chain reaction |
| PfPR_2-10_ | *Plasmodium falciparum* prevalence rate in children 2-10 years old |
| PI | Principal investigator |
| PO | Per os |
| PPB | Pharmacy and Poisons Board (Kenya) |
| PROMOTE | Prevention of Malaria and HIV Disease in Tororo trial (NCT00948896) |
| PYR | Pyrimethamine |
| QTcF | Fridericia’s Corrected QT Interval |
| RDT | Rapid diagnostic test |
| RTS,S/AS01 | Recombinant protein-based *P. falciparum* vaccine (aka “Mosquirix”) |
| SAE | Serious Adverse Event |
| SAP | Statistical Analysis Plan |
| SCA | Sickle cell anemia |
| SD | Standard deviation |
| SMC | Seasonal malaria chemoprevention |
| SNP | Single nucleotide polymorphism |
| SOC | Standard of care |
| SOP | Standard operating procedure |
| SP-AQ | Sulfadoxine/pyrimethamine-amodiaquine |
| SPC | Summary of product characteristics |
| SUSAR | Suspected, Unexpected Serious Adverse Reaction |
| UP | Unanticipated Problem |
| UPIRTSO | Unanticipated Problem Involving Risk to Subjects or Others |
| WHO | World Health Organization |

## STATEMENT OF COMPLIANCE

This trial will be carried out in accordance with Good Clinical Practice (GCP), as required by the International Conference on Harmonisation of Technical Requirements for Registration of Pharmaceuticals for Human Use (ICH) E6. All personnel involved in the conduct of this trial will complete prior to their involvement Human Subjects Protection Training.

I agree to ensure that all staff members involved in the conduct of this study are informed about their obligations in meeting the above commitments.

________________________________

Principal Investigator

________________________________

Signed

______________

Date

________________________________

Principal Investigator

________________________________

Signed

______________

Date

##

## PROTOCOL SUMMARY

| Title: | Enhancing Preventive Therapy of Malaria In children with Sickle cell anemia in East Africa (EPiTOMISE) |
| --- | --- |
| Precis: | This is a randomized, three-arm, open-label, clinical trial of malaria chemoprevention in children with sickle-cell anemia (SCA) at a single site in Homa Bay, Kenya. The study will enroll 246 children under 10 years of age, randomize participants 1:1:1 to one of three malaria chemoprevention regimens, and follow participants monthly for 12 months in order to record clinical episodes of malaria or SCA-related morbidity. Analyses will compare the efficacy of each regimen to prevent malaria and SCA morbidity. |
| Objectives: | Primary: To compare the efficacy of daily proguanil with monthly sulfadoxine/pyrimethanine-amodiaquine (SP-AQ) and with monthly dihydroartemisinin-piperaquine (DP) on the incidence of falciparum malaria in children with SCA.  Secondary: To compare the efficacy of these malaria chemoprevention strategies on the incidence of major complications of SCA. |
| Endpoints: | Primary: incidence of malaria over 12 months.  Secondary: incidence of SCA-related morbidity (pain crises, transfusions, acute chest syndrome), all-cause hospitalization or deaths, severe malaria, drug-resistant malaria parasites |
| Population: | Children of both genders between 1 and 10 years of age with laboratory-confirmed SCA living in malaria-endemic portions of Homa Bay or Migori Counties, Kenya |
| Phase: | 4 |
| Number of Sites Enrolling Participants: | 1: Homa Bay County Hospital, Homa Bay, Kenya |
| Description of Study Agents : | Proguanil administered daily at a dosage determined by participant weight; or, sulfadoxine/pyrimethamine-amodiaquine administered orally over 3 days monthly at a dosage determined by participant age; or, dihydroartemisinin-piperaquine administered orally over 3 days monthly at a dosage determined by participant weight**.** |
| Study Duration: | Approximately 48 months |
| Participant Duration: | 12 months |

## SCHEMATIC OF STUDY DESIGN

Screen potential participants by inclusion and exclusion criteria; obtain history, document. Obtain informed consent.

Prior to

Enollment

Randomize 246 subjects

Enrollment/ Baseline Visit

Acute-care Visits

Final Study

Visit

Routine

Follow-up

Visits

(monthly)

Structured medical history and physical exam

Administer immunizations (if applicable); Enrollment in Kenyan NHIF

Venous blood draw, ECG

Dispense study medications

Structured medical history and physical exam

Outcome, AE, and adherence assessments

Venous blood draw (every third visit)

Test for malaria parasites with RDT*

ECG (in subset of monthly DP participants)

Dispense study medications

Structured medical history and physical exam

Outcome assessment

Test for malaria parasites with RDT*

Structured medical history and physical exam

Outcome, AE, and adherence assessments

Venous blood draw

Test for malaria parasites with RDT*

ECG (in subset of monthly DP participants)

* If temperature of > 37.5C or history of objective or subjective fever in the preceding 24 hours.

## AMENDMENT HISTORY

| Amendment No. | Protocol Version No. | Date Issued | Author(s) of Changes | Details of Changes Made |
| --- | --- | --- | --- | --- |
| 1 | V1.0 |  | Sheila Clapp | Changing approved protocol from V0.4 to V1.0 |
| 2 | V2.0 |  | Sheila Clapp | Added in new exclusion criteria removed the assent for children >7, and other minor changes |
| 3 | V3.0 |  | Sheila Clapp | Removed the provision of the Pneumococcal vaccine at enrollment |
| 4 | V4.0 |  | Sheila Clapp | Removed sentence regarding distributing the study medications package inserts; added dose categories for dihydroartemisinin-piperaquine for children > 25kg; clarified severe malaria language, and other minor corrections. |
| 5 | V5.0 |  | Steve Taylor  Sheila Clapp | Removed co-investigator; clarified safety event classification and reporting |
| 6 | V6.0 |  | Steve Taylor | Further clarified safety event classification, reporting, and study halting procedures; clarified hemoglobin electrophoresis testing lab; added HPLC as an alternate to electrophoresis for HbSS confirmation and HbF quantification.Revised study eligibility criteria, moved enrollment blood tests to screening, and increased hemoglobin blood tests to monthly. |
| 7 | V7.0 |  | Steve Taylor | Revised section 7.2 and added to Section 7.3 study halting guideline |
| 8 | V8.0 |  | Cindy Green  Angie Wu | Revised section 9.4.1, 9.4.2, 9.4.3 and 9.4.4 regarding data analysis. |
| 9 | V9.0 |  | Ernest Ojwang | Revised the Dholuo and Dholuo to English informed consent. |
| 10.0 | V10.0 |  | Sheila Clapp | Minor language change to Dholuo ICF after v9.0 submitted |

## 1 **INTRODUCTION: BACKGROUND & SCIENTIFIC RATIONALE**

### 1.1 Background Information

### 1.1.1 Epidemiology of sickle cell anemia and malaria in Africa

Over 240,000 children with sickle cell anemia (SCA) are born in Africa annually.^1^ This number will increase to over 350,000 annual births of children with SCA by the year 2050.^2^ Without sophisticated medical care, SCA patients in African settings die at young ages: in a Western Kenya cohort of newborns, 25% of SCA children died before their 3rd birthday.^3^ Caring effectively for these children will be a major challenge for developing medical and public health systems in Africa including Kenya (**Figure 1**), and modeling studies suggest that the adequate provision of effective preventive care can substantially reduce the mortality of these children.^2^ Preventive care for SCA children must be evidence-based and tailored to the unique epidemiology of comorbidities in African settings.

Children under 5 years of age in sub-Saharan Africa also suffer the majority of the annual 350 million infections and 500,000 deaths globally.^4^ Reducing this burden is a global public health priority, particularly in areas of high transmission like Western Kenya (**Figure 1**). In the absence of an effective vaccine, global malaria control requires effective treatments and a suite of preventive measures that act upon the parasite, environment, and host. Among these preventive strategies is the administration of prophylactic antimalarials to high risk groups, including pregnant women, infants, and children exposed to seasonal malaria transmission. In these high-risk groups malaria morbidity is substantially reduced by routine periodic intake of effective antimalarials.

Children with SCA are at high risk of life-threatening malaria. In East Africa,^5,6^ children with SCA admitted to the hospital with malaria parasites were more likely to die than those without parasites. Malaria is also a precipitant of sickle-cell pain crises, by unclear mechanisms.^7,8^ It remains unclear how SCA influences the overall risk of malaria, because most studies have been hospital based and therefore unsuited to capture mild episodes.^5,6,9^ The twin observations that malaria is more severe in SCA children and precipitates painful crises in these children indicate that the prevention of *P. falciparum* infections is critical to prolong the survival of SCA children in malaria-endemic areas.^10^

**Figure 1.** Allele prevalence of HbS (left; lighter is larger) and transmission intensity of *P. falciparum* (right; darker is greater) in Kenya. Study site is Homa Bay, and study administration is shared with team members in Eldoret. Images from the Malaria Atlas Project*. https://malariaatlas.org/*

### 1.1.2 Chemoprevention of malaria in high-risk groups

Across Africa, children and pregnant women constitute high-risk groups for malaria and are specifically targeted by prevention programs. These programs include intermittent preventive therapy (IPT) with antimalarials. Among children, IPT for infants (IPTi) with sulfadoxine-pyrimethamine (SP) for and children (IPTc) with SP+amodiaquine (SP-AQ) have been studied widely. Both approaches are safe and reduce the risk of severe malaria by 38% (IPTi-SP)^11^ and 69-87% (IPTc-SP+AQ).^12,13^ Both strategies have been recommended by WHO for malaria control,^14,15^ though IPTc, implemented as seasonal malaria chemoprophylaxis (SMC) in West Africa, has been implemented more quickly. These approaches demonstrate that chemoprevention of high-risk groups to prevent malaria morbidity is feasible and effective.

Children with SCA are higher-risk subsets of infants and children that are targeted by IPTi and ITPc programs, and it is unclear if these regimens that are effective in the general pediatric population are also suitable for children with SCA. Additionally, because the efficacy of chemoprevention programs is critically dependent on the epidemiology of drug resistance, both IPTi^14^ and IPTc^15^ are recommended by WHO only when resistance is below a threshold which is exceeded throughout East Africa. Therefore, current malaria chemoprevention regimens will not protect many children in Africa with SCA.

Six published clinical trials have compared regimens to prevent malaria in people with SCA (**Table 1**). Two randomized studies from Nigeria in the early 2000s investigated the efficacy of daily Proguanil, compared with weekly mefloquine in older children^16^ or placebo and weekly pyrimethamine in younger children.^17^ In the former, the incidence of malaria was comparable between groups; in the latter, there were no differences between groups in the prevalence of parasites during active followup or in the incidence of painful crises, but children receiving proguanil had lower parasite densities. On the basis of this finding, daily Proguanil is recommended by the guidelines promulgated by Ministries of Health across Africa, including Kenya.^18^ Subsequent studies by Nakibuuka *et al*.^19^ and Diop *et al.*^20^ were suboptimal owing to short followup or older participants. Most recently, Olaosebikan *et al.*^21^ randomized 270 Nigerian children and young adults to daily Proguanil or every other month administration of SP-AQ or mefloquine-artesunate (MQAS). In this study, SP-AQ and MQAS were well-tolerated, but the study was not powered to detect clinically-significant differences in malaria incidence. Therefore, there is a lack of adequately-powered studies of monthly treatment in SCA children with extended followup to prevent outcomes with clear clinical impact in East Africa.

| **Study** | **Site** | **Followup** | **N** | **Age** | **Intervention** | **Results** |
| --- | --- | --- | --- | --- | --- | --- |
| Warley et al., 1965^22^ | Uganda | 6-12m | 126 | 75% < 6y | weekly CQ + monthly PCN | Decreased parasite prevalence with CQ+PCN |
| Nwokolo et al.,^16^ 2001 | Nigeria | 6m | 113 | >5y | weekly MQ v. daily Pro | No difference in malaria |
| Eke et al., 2003^17^ | Nigeria | 9m | 101 | 1-16y | weekly PYR v. daily Pro v. placebo | * No difference in *P. falciparum* or SCA events  * Reduced parasite densities in Pro group  * Reduced RBC transfusions in Pro & PYR groups |
| Nakibuuka et al., 2009^19^ | Uganda | 1m | 242 | 6m-12y | monthly SP v. weekly CQ | * 50% reduction in *P. falciparum* malaria in SP group  * non-significant reduction in hospitalizations in SP group |
| Diop et al., 2011^20^ | Mali | 5m | 60 | Mean 23y | monthly SP v. placebo | * non-significant reductions in transfusions and parasite prevalence in SP group |
| Olaosebikan et al., 2015^21^ | Nigeria | 14m | 270 | 63% <10y | Daily Pro v. q2mo SP-AQ or MQAS | * SP-AQ and MQAS well-tolerated  * reduced malaria in SP-AQ and MQAS |

**Table 1**. Published studies of malaria chemoprevention in SCA patients. CQ: chloroquine; PCN: penicillin; PRO: proguanil; MQ: mefloquine; PYR: pyrimethamine; SP: sulfadoxine-pyrimethamine; AQ: amodiaquine; MQ: mefloquine; AS: artesunate; m: months.

### 1.1.3 Study site

Homa Bay, Kenya, is holoendemic for *P. falciparum*: in 2009, the entire population within the catchment area of Homa Bay County Hospital (HBCH) was at risk of *P. falciparum*, and over 20% lived in areas in which the *P. falciparum* parasite rate in children aged 2-10years (*Pf*PR_2-10_, a standard index of malaria transmission intensity) is predicted to exceed 40%.*^23^* From 2006-08, an estimated 9 children per 1000 under 14 years were admitted to HBCH annually for malaria, twice the rate of non-malaria admissions and similar to other lakeside cities.*^24^* In the outpatient setting, in a 2013 study in the HBCH of 677 febrile children, 46% had falciparum malaria.*^25^* Therefore, the transmission of falciparum malaria in and around Homa Bay is substantial.

The SCA clinic in Homa Bay, Kenya, is operated by a collaboration between Academic Model Providing Access to Health Care (AMPATH) HematoOncology and Moi Teaching and Referral Hospital (MTRH). Established in 2013, the SCA clinic at Homa Bay County Hospital (HBCH) currently provides regular care to 1,400 SCA patients, of whom 67% are ≤ 10 years old. Despite comprehensive supportive care, the burden of disease in these SCA children is substantial. All 1,400 patients are enrolled in an electronic registry; clinical enrollment questionnaires indicated substantial morbidity in the preceding 12 months: 49% reported 3 or more hospitalizations, 45% reported at least 3 painful episodes, and 25% reported at least 3 blood transfusions. No malaria-specific datapoints are currently routinely gathered, but 56% of enrollees also reported at least 3 febrile episodes, which, in areas of intense malaria transmission like Homa Bay (see above), are likely to comprise many episodes of falciparum malaria. Because of this, on followup visits, 86% endorsed receipt of Proguanil or other antimalarial prophylaxis and 48% endorsed penicillin prophylaxis. This SCA clinic’s patient population, disease burden, and existing infrastructure allow immediate, direct testing of new strategies to prevent malaria in SCA children in Africa.

### 1.1.4 Potential efficacy of chemoprevention agents

Studies of the efficacy of Proguanil prophylaxis are detailed above (**Table 1**). Notably, molecular genetic studies indicate that parasite susceptibility to Proguanil is significantly reduced by mutations in the parasite’s *dhfr* gene.^26,27^ These mutations are very common in Western Kenya,^28^ and it is unclear if the clinical prophylactic effect of Proguanil is retained in areas of East Africa where these parasite mutations are prevalent.

Is SP-AQ likely to be effective against *P. falciparum* in Western Kenya? The prevalence of parasite molecular markers of SP resistance is high in Western Kenya,^28^ but SP-AQ has retained appreciable activity against parasites in therapy studies. In a 2003 study in Mbita,^29^ adequate clinical response was achieved in 87% of children with uncomplicated falciparum malaria that were treated with SP-AQ. AQ, when coupled with artesunate as AS-AQ, is an effective partner drug for uncomplicated malaria therapy.^30^ For prophylaxis, few studies of SP-AQ have been conducted in areas with parasite resistance similar to contemporary Kenya. In a study of intermittent preventive therapy in schoolchildren aged 5-18 years in Western Kenya, thrice-yearly SP-AQ reduced anemia and improved school performance.^31^ This study was conducted in 2005-6 when the prevalence of markers of SP resistance had reached nearly 100%, indicating that, despite the high prevalence of molecular SP resistance in Kenyan parasites, SP-AQ was effective as chemoprevention.

DP is an artemisinsin-combination therapy (ACT) that is highly effective as both therapy and chemoprevention. In over 1,000 children in West, Central, and Eastern Africa, the day 28 PCR-corrected efficacy of DP for uncomplicated malaria in a per-protocol population was 95%; this efficacy was non-inferior to the first-line therapy artemether-lumefantrine (AL), and the reduced incidence of new infections in the DP group during the 42-day followup also indicated a substantial post-treatment prophylactic effect.^32^ This latter effect was confirmed in a Cochrane review, in which DP reduces the number of new infections after treatment by 30%.^33^ In chemoprevention studies, monthly DP has reduced the incidence of malaria by 58% to 77%.^34,35^ DP’s high parasiticidal efficacy of DP and prolonged post-treatment prophylaxis make it an attractive candidate for intermittent preventive therapy.

SP-AQ has a favorable safety profile. Agranulocytosis or hepatotoxicity were reported in travelers receiving weekly AQ,^36^ but recent use of SP-AQ in large trials of seasonal malaria chemoprevention (SMC) has enabled a comprehensive accounting of adverse effects in African children. In over 100,000 courses of SP-AQ administered as SMC to over 35,000 children,^37^ there were no cases of Stevens-Johnson Syndrome, serious blood dyscrasias, or hepatic damage; the most common AE was vomiting after SP-AQ (5.9%). In over 1,000 Gambian children allocated to receive SP-AQ, SP-Piperaquine, or DP for three monthly doses^38^ and who were actively queried for AEs after each dose, the most common symptoms after SP-AQ were cough (15%), diarrhea (13%), and anorexia (8%), but these frequencies were similar to those receiving the other regimens and lower than controls who had received no drug regimen, suggesting that they were not attributable to SP-AQ itself.

Proguanil safety has been investigated most closely when administered in combination with atovaquone (Malarone). In a meta-analysis of atovaquone-proguanil (AP) use for daily prophylaxis in travelers and residents of endemic areas, the rate of AEs was not higher than that for placebo,^39^ including data from a study of school-age children in Gabon in which rates were similar between AP (Proguanil dosing between 1.3 – 2.4 mg/kg) and placebo groups.^40^

DP is also well-tolerated. In two studies of DP administered monthly to children as prophylaxis in Burkina Faso,^34^ and in Gambia,^38^ the most common reported adverse events were cough, diarrhea, and vomiting, similar to those receiving SP-AQ. In a chemoprevention study in Ugandan children, there were fewer grade 3-4 adverse events in the monthly DP arm than in the placebo, monthly SP, or daily trimethoprim-sulfadoxine arms.^35^ Piperaquine harbors the potential for QT prolongation,^41^ and a recent trial of DP as prophylaxis in healthy Thai adults was terminated early owing to QTc prolongation of 46ms.^42^ This phenomenon was not observed in a treatment study of over 1,500 African children under 5 years old.^32^ Few studies with repeated dosing have investigated this phenomenon: QTc intervals were reportedly normal in the PROMOTE study of SMC in Ugandan children,^35^ and trials of monthly DP prophylaxis in Thai adults^43^ and Ugandan,^44^ Gambian,^38^ and Senegalese children^45^ have indicated excellent tolerability of repeated DP, but not included formal QTc evaluations. Notably, however, sickle cell anemia itself may increase the risk of QTc prolongation:^46^ in one study of SCA patients between 10-24 years, the prevalence of a QTc interval > 440msec was 38%,^47^ possibly owing to chronically-elevated pulmonary arterial pressures, recurrent acute chest syndrome, and hemolysis.^48^

### 1.2 **Rationale**

The EPiTOMISE trial will evaluate the efficacy on malariologic and hematologic endpoints of two monthly malaria chemoprevention regimens compared with the standard of care. The rationale for this project is that intermittent therapy with highly-active antimalarials is highly effective as malaria prevention in other high-risk groups. Therefore, intermittent therapy with more efficacious antimalarials may provide adequate protection from malaria and reduce the incidence of hematologic morbidity owing to SCA. Our hypotheses are that:

1. both monthly SP-AQ and monthly DP will be superior to daily Proguanil at preventing falciparum malaria;
2. both monthly SP-AQ and DP will be superior to daily Proguanil at preventing painful events; and
3. receipt of chemoprevention will increase the prevalence of markers of resistance in breakthrough infections in recipients of SP-AQ or Proguanil but not DP.

### 1.3 Potential Risks and Benefits

### 1.3.1 Known potential risks

*Privacy.* Care will be taken to protect the privacy of the participants and their guardians, but there is a risk that others may inadvertently see patient medical information and thus compromise privacy in the delivery of medical care.

*Blood drawing.* Risks of venipuncture and heel- or fingerpricks include pain, self-limited

bleeding, and infection of the soft tissues.

*Study medications.* Proguanil is the current standard of care for chemoprevention of malaria in children with SCA in Kenya. Possible adverse reactions that are listed in the Summary of Product Characteristics (SPC) include allergic reactions, mild gastric intolerance including diarrhea or constipation, mouth ulceration, stomatitis, skin reactions, reversible hair loss, drug fever, cholestasis, or hematologic changes in recipients with renal insufficiency.^49^ In clinical studies using Proguanil alone or co-formulated with Atovaquone, Proguanil has been well-tolerated.

SP-AQ is a combination therapy comprised of sulfadoxine and pyrimethamine (two antifolate antimicrobials) co-administered with amodiaquine. Common adverse effects observed with amodiaquine include headache, vomiting, abdominal pain, diarrhea, pruritis, and fever, while rare but severe side effects include retinopathy and hepatitis. Common adverse effects observed with SP include abdominal pain, diarrhea, urticarial, pruritis, skin eruptions, headache, and fever.^50^ As noted above, large-scale deployment of SP-AQ as seasonal malaria chemoprevention in West Africa has suggested a favorable safety profile in children when used as preventive therapy.

DP is an artemisinin-combination therapy consisting of the artemisinin derivative dihydroartemisinin and the bisquinoline piperaquine. DP is also well-tolerated. Common adverse effects in children after DP receipt include cough, anemia, thrombocytopenia, leukopenia, anorexia, QTc prolongation, vomiting, diarrhea, abdominal pain, dermatitis, rash, and asthenia.^51^ As noted above, in a chemoprevention study in Ugandan children, there were fewer grade 3-4 adverse events in the monthly DP arm than in the placebo, monthly SP, or daily trimethoprim-sulfadoxine arms.

*Alternative study medications.* Other regimens have been studied as chemoprevention in various groups of children in Africa, including monthly dosing of mefloquine-artesunate, mefloquine alone, SP-artesunate, or amodiaquine-artesunate, or daily trimethoprim-sulfamethoxazole. We selected DP as a treatment arm over these options owing to the protracted period of prophylaxis conferred by the prolonged half-life of piperaquine, the putative absence of resistance to either component, and emerging evidence that, in other high-risk groups, DP is very effective as prophylaxis. We selected SP-AQ as a treatment arm owing to abundant data indicating favorable safety and tolerability profiles, its low cost, and its effectiveness in prior studies of prevention despite prevalent parasite drug resistance.

### 1.3.2 Known potential benefits

This study will potentially have immediate and long-range potential benefits to the human participants. Immediately, participants will receive free study medications to prevent malaria and treat malaria, free medications (for those under 5 years of age) to prevent bacterial infections, monthly comprehensive assessments and free testing for malaria parasites when they have subjective or objective fevers, a catch up routine immunization, and enrollment in the Kenya National Hospital Insurance Fund (NHIF). These services have the potential to reduce morbidity from both malaria and SCA.

Long-range potential benefits include improved growth owing to reduced illness in the childhood period, improved school attendance and perhaps performance owing to reduced absenteeism, improved supportive care, and likely improved survival could result from the trial if the results of the trial enhance guidelines for care of children with SCA in Africa.

## 2 OBJECTIVES AND PURPOSE

The primary objective of the EPiTOMISE trial is to compare the efficacy of daily Proguanil with that of monthly SP-AQ or monthly DP to prevent *P. falciparum* malaria in children with SCA.

The secondary objectives of this trial are to:

1. compare the efficacy of daily Proguanil, monthly SP-AQ, and monthly DP to prevent painful events in children with sickle cell anemia, and
2. compare the impact of malaria chemoprevention regimens on molecular markers of parasite drug resistance to Proguanil, SP-AQ, and DP.

## 3 STUDY DESIGN AND ENDPOINTS

### 3.1 Description of the Study Design

This study will be a three-arm, single-site, open-label randomized clinical trial in children with SCA of malaria chemoprevention comparing daily Proguanil (current standard of care), monthly SP-AQ, or monthly DP. The EPiTOMISE study is powered on the incidence of clinical malaria, and include as secondary outcomes other clinical and parasitologic endpoints as well as measures of SCA morbidity.

### 3.2 Primary Endpoint

The primary endpoint is incidence of clinical malaria expressed as episodes per person-year at risk. Time at risk will begin when study participants are randomized and receive study drug (i.e. begin chemoprevention) and end when participants reach the end of the observation period. Clinical malaria will be detected by passive surveillance and will be defined as:

- Uncomplicated malaria:
  - Presence of *P. falciparum* parasitemia of any density AND
  - Temperature of ≥ 37.5C or history of objective or subjective fever in the preceding 24 hours

OR

- Case meeting the definition of severe malaria according to WHO criteria (see Section 3.3 for definition)

Those screening positive for fever or other signs or symptoms suggestive of severe malaria will be tested for malaria parasites by rapid immunochromatographic diagnostic test (RDT). The RDT will be used to guide clinical treatment. Only RDT-positive events will be recorded as primary endpoints; suspected malaria cases that are not confirmed by RDT will be recorded as secondary endpoints (see below).

### 3.3 Secondary Endpoints

Multiple secondary endpoints of malaria- and sickle-cell-related morbidity will be captured. These secondary endpoints and their definitions and ascertainment are:

1. Severe malaria, defined as a positive RDT AND one or more of the following:^52^
   ● Impaired consciousness – Glasgow coma score <3 in children

● Prostration – Generalized weakness so that the person is unable to sit, stand, or walk without assistance

● Multiple convulsions – More than two episodes within 24 hours

● Acidosis – A base deficit of >8 mEq/L, a plasma bicarbonate level of <15 mmol/L, or venous plasma lactate ≥5 mmol/L. Severe acidosis manifests clinically as respiratory distress (rapid, deep, labored breathing).

● Hypoglycemia – Blood or plasma glucose <40 mg/dL (<2.2 mmol/L)

● Severe malarial anemia – Hemoglobin concentration ≤5 g/dL or hematocrit ≤15 percent in children with parasite count >10,000/mcL

● Renal impairment – Plasma or serum creatinine >3 mg/dL (265 mcmol/L) or blood urea >20 mmol/L

● Jaundice – Plasma or serum bilirubin >50 mcmol/L (3 mg/dL) with a parasite count >100,000/mcL

● Pulmonary edema – Radiographically confirmed or oxygen saturation <92 percent on room air with respiratory rate >30/min, often with chest indrawing and crepitations on auscultation

● Significant bleeding – Including recurrent or prolonged bleeding from the nose, gums, or venipuncture sites, hematemesis, or melena

● Shock – Compensated shock is defined as capillary refill ≥3 seconds or temperature gradient on leg (mid to proximal limb) but no hypotension. Decompensated shock is defined as systolic blood pressure <70 mmHg in children, with evidence of impaired perfusion (cool peripheries or prolonged capillary refill).

● *P. falciparum* parasitemia >10 percent (>500,000/mcL)

*Ascertainment*: Signs of severe malaria will be captured during routine or acute care clinic visits.

1. Hospitalization for malaria, defined as hospitalization at HBCH or other inpatient facility with admitting diagnosis of malaria and microbiologic confirmation of *P. falciparum* infection by RDT.

*Ascertainment*: Review of HBCH pediatric ward admission logs and query of caregivers at followup visits.

1. Light microscopy (LM)-positive malaria, defined as the reported presence of *P. falciparum* parasites detected by LM irrespective of RDT or other detection results.

*Ascertainment*: Review of HBCH and other healthcare facility records.

1. Unconfirmed malaria, defined as the receipt of antimalarials for suspected malaria episodes that were not confirmed by any objective diagnostic test.

*Ascertainment*: Review of HBCH and other healthcare facility records.

1. Fatal malaria, defined as death during hospitalization for malaria, as defined above.

*Ascertainment*: Review of HBCH pediatric ward inpatient logs and query of caregivers at followup visits.

1. Asymptomatic parasitization, defined as the presence of parasites during routine followup visits as detected by PCR in patients without fever or a history of recent fever.

*Ascertainment*: Dried blood spots will be collected during routine visits from all participants and tested post-hoc for the presence of *P. falciparum* parasites using a real-time PCR assay.

1. Painful events, defined as pain lasting 2 hours or more without obvious cause (either as inpatients or outpatients).^53^

*Ascertainment*: Query using structured interview at each clinic visit.

1. Dactylitis, defined as pain or tenderness with or without swelling of the hands or feet.^53^

*Ascertainment*: Query using structured interview at each clinic visit.

1. Transfusions, defined as the receipt of red blood cells from any caregiver for any indication.

*Ascertainment*: Query using structured interview at each clinic visit.

1. Acute chest syndrome, defined as a new pulmonary infiltrate and at least 3 of the following findings: chest pain, temperature elevation > 38.5°C, tachypnea, wheezing, or cough.

*Ascertainment*: Structured history and physical exam at each scheduled and acute care visit, or review of outside records.

1. All-cause hospitalizations, defined as hospitalization at HBCH or other inpatient facility with any admitting diagnosis.

*Ascertainment*: Query using structured interview at each clinic visit.

1. All-cause deaths, defined as death by any cause.

*Ascertainment*: Query using structured interview during followup.

1. Molecular markers of parasite drug resistance, defined as the presence of single nucleotide polymorphisms (SNPs) in parasite genes that confer resistance to the study drugs.

*Ascertainment*: Parasites detected in dried blood spots from asymptomatic or symptomatic individuals will be subjected to PCR amplification of the genes of interest followed by Sanger sequencing for gene mutations.

## 4 STUDY ENROLLMENT AND WITHDRAWAL

### 4.1 Participant Inclusion Criteria

In order to be eligible to participate in this study, an individual must meet all of the following criteria:

1. Age greater than 12 months and less than 10 years at enrollment;
2. Current attendance at or willingness to attend the study SCA clinic at HBCH;
3. Residence in either Homa Bay County or the Rongo or Awendo sub-counties of Migori County;
4. Confirmed hemoglobin genotype of HbSS by electrophoresis or high-performance liquid chromatography (HPLC);
5. No immediate, apparent, or reported plans to relocate residence from Homa Bay County or the Rongo or Awendo sub-counties of Migori County in the next 2 years;
6. Ability to take oral medication and be willing to adhere to the medication regimen or caregiver willingness to give the medical regimen as prescribed;
7. Ability and willingness of parent or legally authorized representative (LAR) to give informed consent;
8. Hemoglobin concentration > 6.5 g/dL at screening;
9. Alanine aminotransferase (ALT) <= 50 U/L at screening.

### 4.2 Participant Exclusion Criteria

An individual who meets any of the following criteria will be excluded from participation in this study:

1. Taking routine antimalarial prophylaxis for another indication (including co-trimoxazole for HIV infection);
2. Known allergy or sensitivity to sulfadoxine, pyrimethamine, amodiaquine, proguanil, dihydroartemisinin, piperaquine, artemether, lumefantrine, penicillin (if under 5 years old), or derivatives of these compounds;
3. Known chronic medical condition other than SCA (i.e. malignancy, HIV) requiring frequent medical attention;
4. Currently participating in another clinical research study, or having participated in one in the prior 30 days;
5. Living in the same household as a previously-enrolled study participant;
6. Chronic use of medications known to prolong the QT interval in children (see Appendix L);
7. Fridericia’s corrected QT interval (QTcF) interval > 450msec.
8. Receipt of a transfusion of red blood cells in the 120 days prior to screening.

### 4.3 Strategies for Recruitment and Retention

Potential participants will be screened for inclusion in the existing pediatric SCA clinic at HBCH. Currently, SCA patients are cared for in two parallel clinics: 1) the pediatric medical officer staffs a weekly SCA clinic, at which approximately 20 patients are evaluated once every 3 months, and 2) the AMPATH HematoOncology outreach team staffs a monthly 2-day SCA clinic at HBCH, during which they evaluate 120-140 patients.

Potential participants will be screened for eligibility by study personnel when attending either clinic. If they meet all inclusion criteria and do not meet any exclusion criteria, they will be offered enrollment following informed consent. Children who do not meet inclusion criteria, or who meet exclusion criteria, or who do not want to participate in the trial, will receive subsequent care in the SCA clinic as determined by the standard of care at HBCH. For this study, only African children will be recruited.

Retention will be enhanced by several methods. Participants will be compensated for each routine follow-up visits and the final study visit, including follow-up visits for repeat ECGs in the children that have been selected for ECG safety monitoring while allocated to DP; participants will not be compensated for acute-care visits. Compensation will be provided to participant’s guardian. In order to improve adherence to study visit schedule, study personnel will contact all study participants or their guardians in the week prior to their scheduled routine followup visit.

### 4.4 Participant Withdrawal or Termination

### 4.4.1 Reasons for withdrawal or termination

Participants are free to withdraw from participation in the study at any time upon request. In addition, an investigator may terminate participation in the study for any of the following reasons:

1. Any clinical adverse event (AE), laboratory abnormality, or other medical condition or situation occurs such that continued participation in the study would be detrimental to the participant;
2. The participant meets an exclusion criterion (either newly developed or not previously recognized) that precludes further study participation;
3. Movement out of the study area or inability to be located for > 60 consecutive days since the preceding clinic visit;
4. Withdrawal of informed consent;
5. Demonstrated or likely inability to comply with the study schedule or procedures;
6. Participant or guardian requests to terminate participation in the study.

### 4.4.2 Handling of participant withdrawals or termination

Randomized subjects who withdraw from the study will not be replaced. If participants are withdrawn for reasons 3) or 4) above, no further study assessments will be performed. For withdrawals for other reasons above, follow-up testing will be at the discretion of the participant and their regular medical provider. All data collected prior to withdrawal will be retained and used in the study database. No further testing will be done if the participant withdraws informed consent or requests to terminate participation.

### 4.5 Premature Termination or Suspension of Study

The study may be temporarily suspended or prematurely terminated if there is sufficient reasonable cause. The decision to prematurely terminate the study will require the agreement of the DSMB. Written notification, documenting the reason for study suspension or termination, will be provided by the DCRI to the study investigators, partnering institutions, funding agency, and regulatory authorities, as applicable. If the study is prematurely terminated or suspended, the PI will promptly inform the Duke University IRB, the Kenya Pharmacy and Poisons Board (PPB), and the Moi University Institutional Research Ethics Committee (IREC), the DSMB (for suspensions) and the sponsor and will provide the reason(s) for the termination or suspension.

Circumstances that may warrant termination or suspension include, but are not limited to:

1. Determination of unexpected, significant, or unacceptable risk to participants;
2. Insufficient compliance to protocol requirements;
3. Data that are not sufficiently complete and/or evaluable;
4. Poor enrollment.

The study may resume once concerns about safety, protocol compliance, data quality are addressed to the satisfaction of the DCRI and, as applicable, overseeing IRBs and regulatory agencies.

## 5 STUDY AGENT

### 5.1 Study Agents and Control Description

Participants will be randomized to one of 3 therapies: daily Proguanil, monthly SP-AQ, or monthly DP. The study agent is NOT to be used for any purpose other than that outlined in this protocol.

1. Proguanil is the current standard of care for chemoprevention of malaria in children with SCA in Kenya. Proguanil is a dihydrofolate reductase inhibitor and therefore it interferes with the folic-folinic acid system of *P. falciparum* parasites. The active metabolite of Proguanil – cycloguanil -- binds to the enzyme dihydrofolate reductase in the malaria parasite and thereby prevents the completion of schizogony.
2. SP-AQ consists of sulfadoxine, pyrimethamine, and amodiaquine. Amodiaquine is a synthetic 4-aminoquinoline antimalarial with schizonticidal action on *P. falciparum* by destroying intraerythrocytic forms. Its mechanism of action is incompletely understood. Pyrimethamine is a diaminopyrimidine with antimalarial activity that is mediated by the inhibition of plasmodial dihydrofolate reductase and thus the indirect blocking of the synthesis of nucleic acids in the malaria parasite. Sulfadoxine is a sulfonamide, and therefore a competitive antagonist of p-aminobenzoic acid, which results in competitive inhibition of dihydropteroate synthase, the enzyme in *P. falciparum* which is responsible for the incorporation of p-aminobenzoic acid in the synthesis of folic acid.
3. DP consists of dihydroartemisinin and piperaquine. Dihydroartemisinin is a semi-synthetic derivative of artemisinin and the active metabolite of all artemisinin compounds. The exact mechanism of action of dihydroartemisinin against *P. falciparum* parasites is incompletely understood, but appears to result in part from heme-mediated activation that causes pervasive free-radical damage to parasite membranes. Piperaquine is a bisquinoline antimalarial with an incompletely understood mechanism of action against malaria parasites.

### 5.1.1 Acquisition

Study medications are commercially available in Kenya and will be procured and packaged for use by the study participants. When possible, study medications will be acquired from manufacturers who have been prequalified by WHO to produce the antimalarial as a finished pharmaceutical product.

Study medication will be provided to all consented and randomized participants free of charge.

### 5.1.2 Formulation, appearance, packaging, and labeling

All study agents are commercially-available oral medications and will not be modified in constitution from their manufactured state. Agents will be portioned to enable weight- or age-based dosing as described in Section 5.1.4. Agents will be packaged by study pharmacists at the MTRH Investigational Pharmacy in monthly allotments. These allotments will be clearly coded to prevent potential mis-distribution to study participants. The package insert will be available for review at any time during the study.

### 5.1.3 Product storage and stability

Storage requirements will be per product label. Each dose of study drug will be prepared according to the subject’s weight or age and treatment assignment by the pharmacist or other appropriately licensed and authorized personnel. Details on preparation and storage of study material will be provided in the Pharmacy Manual as a part of the study Manual of Procedures (MOP).

### 5.1.4 Dosing and administration

Administration of all study drugs will be open label. Study drugs will be administered at

home by the study participant’s parents or guardians. Although this is an efficacy trial, it will not be practical to administer the study drugs utilizing a “directly observed therapy” approach, owing to the daily dosing of Proguanil and the multiple days of administration of both SP-AQ and DP; this asymmetry in dosing would result in potential bias if the initial doses were directly observed. Parents or guardians will be given pre-packaged study drugs along with dosing instructions. Study participants will be seen in the clinic for a final study visit at 12 months, at which time parents or guardians will be given clear instructions to return to their usual medical providers for ongoing care. Plans for the monitoring of safety of study drugs are detailed in Section 7.

1. Dosing of daily proguanil will be approximately 3mg/kg/day, consistent with clinical practice and current recommendations in Kenya, rounded to the nearest 25mg increment:^18^

| **Weight (kg)** | **No. of 100mg tabs Proguanil daily** |
| --- | --- |
| ≤ 13 | ¼ |
| 13-21 | ½ |
| 21-29 | ¾ |
| > 29 | 1 |

1. Dosing of SP-AQ will be as follows:

| **Age (y)** | **Day 1** | **Day 2** | **Day 3** |
| --- | --- | --- | --- |
| 1-5 | 1 tablet 500/25mg SP  1 tablet 150mg AQ | 1 tablet 150mg AQ | 1 tablet 150mg AQ |
| 6-10 | 1.5 tablets 500/25mg SP  1.5 tablets 150mg AQ | 1.5 tablets 150mg AQ | 1.5 tablets 150mg AQ |

This is consistent with WHO recommendations for SMC with SP-AQ in West Africa.^15^

1. The weight-based dosing of tablets of 40/320mg of DP is described below:

| **Weight (kg)** | **No. of 40/320mg tabs DP daily for 3 days** |
| --- | --- |
| ≤ 5 | ¼ |
| 6-10 | ½ |
| 11-14 | ¾ |
| 15-19 | 1 |
| 20-23 | 1 ¼ |
| 24-25 | 1 ½ |
| 26-35 | 2 |
| 36-59 | 3 |

### 5.1.5 Route of administration

All medications will be administered orally.

### 5.1.6 Duration of therapy

Participants will receive study drug for 12 consecutive months.

### 5.1.7 Tracking of dose

Proper dosing of Proguanil (based on weight), SP-AQ (based on age) and DP (based on weight) will be confirmed at each monthly study visit by measurement of weight and confirmation of age. Adherence to each of the study drugs will be assessed at each monthly evaluation by structured queries. Study drugs will be kept in a dedicated study pharmacy space maintained and accessed only by authorized study personnel. Study drugs will be dispensed by pharmacy staff to study nurses, and the study nurses will dispense the study medications to the subjects at the time they are seen for their routine monthly visits (scheduled approximately every 30 days). Detailed records will be kept on the amount of study drug distributed to each study participant.

### 5.2 Study Agent Accountability Procedures

A registry of the study medication, current product labels, and Certificates of Analysis, provided by suppliers, will be maintained within the regulatory binder for the study. In addition, the study pharmacist will also maintain study drug accountability records in the pharmacy. The date received, lot number, expiration date, and date used will be recorded for each of the study medications, as well as the information pertaining to specific drug dispensed to each patient (e.g., lot number, amount dispensed). Monthly inventory of all study medications will be conducted and the information collected from those inventories will be documented in the pharmacy records.

## 6 STUDY PROCEDURES AND SCHEDULE

### 6.1 Study Procedures/Evaluations

### 6.1.1 Study specific procedures

To ensure the proper timing of routine assessments, study participants will be scheduled to come to the study clinic 30 days (+/- 5 days) following the preceding routine followup visit. If a study participant is not seen in the study clinic within this time, study staff will contact by phone the participants parent/guardian and if necessary visit the home and ask the patient’s parent/guardian to come to the clinic.

These routine assessments conducted during these monthly follow up visits will include:

1. Queries regarding medical care obtained outside of the study site and medications taken outside study medications, including a request to review records and prescriptions;
2. Assessment of adherence to study medications and to insecticide-treatment bednet (ITN) use;
3. Routine interval medical history, review of systems, and physical examination (including vital signs and weight);
4. Blood testing for routine laboratory tests:
   1. Venous blood will be collected for CBC, ALT, creatinine every 90 days and hemoglobin electrophoresis/HPLC for HbF quantitation every 180 days;
   2. Fingerprick blood will be collected at each visit for hemoglobin measurement and to be archived as a dried blood spot for post-hoc detection of malaria parasites and parasite genotyping by PCR.
5. In the first 20 children allocated to the monthly DP regimen who live in Homa Bay town, ECG’s will be conducted 4-6 hours following the 3^rd^ daily (and final) dose of monthly DP.

In addition, all participants with a temperature > 37.5C or history of objective or subjective fever in the preceding 24 hours will be tested for malaria parasites using an RDT.

### 6.1.2 Standard of care study procedures

Clinical care for conditions other than SCA or possible malaria will be provided by non-study clinicians. All efforts will be made to assist in prompt referral to additional care for participants as needed. In addition, diagnostic or therapeutic maneuvers during acute care or followup visits beyond those described below will be considered standard of care and not part of the clinical protocol.

### 6.2 Laboratory Procedures/Evaluations

### 6.2.1 Clinical laboratory evaluations

During screening, participants will have a blood sample taken for baseline measurements of CBC, ALT, and creatinine and confirmation and documentation of their HbSS disease by electrophoresis or HPLC in order to determine eligibility for enrollment.

At month 1, 2, 4, 5, 7, 8, 10, and 11, fingerprick blood will be tested for hemoglobin concentration.

At month 3, 6, 9, and 12, we will collect venipuncture blood for the following clinical laboratory tests:

1. Hematology: CBC; hemoglobin electrophoresis or HPLC for HbF quantification (only at month 6 and month 12 visits)
2. Chemistry: creatinine, ALT.

At baseline, followup, and acute-care visits through month 12 at which the participant has a temperature ≥37.5C or a history of subjective or objective fever in the preceding 24 hours, blood will be collected by fingerprick for point-of-care testing for malaria parasites using an RDT.

Hematology (exclusive of hemoglobin electrophoresis/HPLC) and chemistry tests will be performed by an accredited clinical laboratory in Western Kenya.

Hemoglobin assessment by electrophoresis or HPLC will be performed by an established academic clinical or commercial laboratory.

### 6.2.2 Other assays or procedures

Venous blood will be collected and stored in a PAXgene tube in order to enable exploratory studies of biomarkers of severity of SCA, malaria, or other infections in children with SCA. This PAXgene tube sample will be collected only from participants who separately consent for this, and will be collected during a venous blood draw at a routine visit.

At enrollment, monthly followup, and acute-care visits through month 12 (final visit), fingerprick blood will be stored as a dried blood spot (DBS) for post-hoc malaria parasite PCR detection and genotyping. This will be performed in Dr. Taylor’s research laboratory at Duke University in Durham, NC, USA. For detection, assays will be used that target *P. falciparum* gene targets.^54^ For genotyping, assays will be used to sequence parasite markers of drug resistance, vaccine-resistance, and other parasite gene markers for strain typing.

### 6.2.3 Specimen preparation, handling, and storage

For hemoglobin electrophoresis/HPLC (screening and 6-monthly routine visits), 1mL of blood will be collected by venipuncture into a vacutainer labeled with the participants screening or participant ID, transported to MTRH at room temperature in a cool box, and stored at room temperature for up to 1 week prior to testing. Procedures for hemoglobin electrophoresis or HPLC and HbF quantitation will be described in the study MOP.

PAXgene tubes will be labeled with participant ID, refrigerated for up to 5 days at HBCH, then transported and frozen at Moi University at -20C.

DBS will be stored on standard chromatography filter paper as duplicate ~50 microliter blood spots. After drying at ambient temperature, these will be stored in sealed, individual plastic bags with desiccant on site. Each DBS will be labeled with a printed label with participant study ID and date of collection. The procedures will be detailed in the study MOP.

### 6.2.4 Specimen shipment

Whole blood specimens for hemoglobin electrophoresis/HPLC will be shipped on a regular basis for testing within 48 hours from the time of collection.

DBS and PAXgene tubes will be shipped in IATA-approved containers without cooling using standard carriers (e.g. DHL) to Dr. Taylor’s lab at Duke University at the conclusion of the final followup visit for all patients. Included will be Dr. Taylor’s import permit from the US Centers for Disease Control and Prevention. The electronic inventory cataloging all shipped specimens will be transmitted electronically.

Prior to shipping, export permission will be sought from the Kenyan Ministry of Health, and import permission sought from the US Centers for Disease Control and Prevention.

### 6.3 Study Schedule

### 6.3.1 General screening procedures

Potential study participants between 9 months and 10 years of age and their parents/guardians will be identified through the HBCH SCA clinic and, if necessary owing to poor enrollment, affiliated clinics in Homa Bay County. Parents/guardians will be approached about participating in the study and will be provided an information sheet about the requirements of the study (see Appendix A – English, Appendix B – Kiswahili, Appendix C – English back-translation from Kiswahili, Appendix D – Dholuo, Appendix E – English back translation from Dholuo). If the parents or guardians are agreeable to screening for participation in the study the child and parents will either be escorted to the study clinic or made an appointment to return at a later date. During the pre-screening process, study physicians will assess for initial eligibility criteria through conversation with the parent/guardian (full Inclusion and Exclusion Criteria listed in Sections 4.2 and 4.3). All participants who pass pre-screening will be assigned a screening ID study number.

Study personnel will conduct the informed consent discussion in the study clinic with the subjects’ parent(s) or guardian(s). Informed consent will be conducted in either English, Kiswahili, or Dholuo as appropriate, the consent forms will be translated into Kiswahili and Dholuo and back-translated into English to check for any loss or change of meaning. Following the informed consent discussion, parents or guardians will be asked by the study personnel to sign a written consent form approved by the Moi University Institutional Research Ethics Committee (IREC) for their child to participate in the research study (Appendix F – English, Appendix G – Kiswahili, Appendix H – English back-translation from Kiswahili, Appendix J – Dholuo, Appendix K – English back-translation from Dholuo). If the parent or guardian is unable to read or write, their fingerprint will substitute for a signature, and a signature from a witness to the informed consent discussion will be obtained.

#### 6.3.1.1 Screened children less than 12 months of age

Potential study participants who pass pre-screening but are not yet 12 months of age will be given an appointment to the study clinic for a time after their first birthday for thorough assessment of study eligibility, informed consent, and potential enrollment.

#### 6.3.1.2 Screening procedures after informed consent

Participants will first have eligibility assess by review of medical information and history. Those who meet criteria based on demographics and medical history will have an ECG performed to assess for baseline QTc interval length using Fridericia’s heart rate correction method (QTcF). Any QTcF interval > 450 msec will be confirmed by repeat ECG. Potential participants with QTcF intervals > 450 msec on this repeated ECG will be excluded from enrollment and therefore further screening procedures will be halted.

Those with QTcF ≤ 450msec will have venipuncture blood drawn for CBC, ALT, creatinine, and hemoglobin electrophoresis or HPLC (see Section 6.2.1).

#### 6.3.1.3 Screened children with recent transfusion of red blood cells

Potential study participants who are excluded from screening owing to the receipt of a transfusion of red blood cells in the 120 days prior to screening will be eligible to be re-screened at a subsequent visit.

### 6.3.2 Enrollment

On the day of enrollment, all eligibility criteria will be confirmed.

Participants will be randomized 1:1:1 by block allocation to either daily Proguanil, monthly SP-AQ, or monthly DP. Randomization will be achieved using permuted blocks of undisclosed size using a random number generator in SAS version 9.4 or higher (SAS Institute, Cary, NC).

Treatment assignments will be generated by computer as each participant is enrolled. The trial is open-label.

At the enrollment visit, all children will receive:

1. Meningococcal immunization with quadrivalent vaccine (if > 2y and no prior receipt);
2. Enrollment in the Kenyan NHIF;
3. If < 5 years old, a 30-day supply of daily penicillin prophylaxis, dosed as follows:
   1. 1-3y: Penicillin V 125mg PO BID;
   2. >3y-5y: Penicillin V 250mg PO BID.
4. Allocated study medications, at dosages as listed in Section 5.1.4 above.

At enrollment, the following will be collected from all children:

1. Structured demographic and clinical questionnaire;
2. Structured physical examination including vital signs;
3. Fingerprick blood stored as DBS.

### 6.3.3 Routine follow-up visits

Participants will be scheduled for routine followup assessments at HBCH through month 12. During these routine visits, data will be collected, as below.

Routine follow-up visit (every 30 days, +/- 5 days):

1. Performance of structured medical history, including:
   1. Adverse events potentially related to study medications, principally Suspected Unexpected Serious Adverse Reactions (SUSARs; see Section 7.4.1).
   2. Adherence to study medications;
   3. Study outcomes, including those recorded at an outside medical facility:
      1. Microbiologically-confirmed malaria since last visit;
      2. Acute malaria treatments since last visit;
      3. Painful events, transfusions, dactylitis, acute chest syndrome;
      4. Hospitalization;
2. Performance of structured physical examination, including:
   1. Vital signs with weight;
   2. Anthropomorphic measurements;
   3. General physical survey.
3. Collection of blood for:
   1. Dried blood spot for PCR parasite detection (by fingerprick);
   2. Hemoglobin testing in month 1, 2, 4, 5, 7, 8, 10, and 11 (by fingerprick);
   3. CBC, creatinine, ALT in month 3, 6, 9, and 12 (by venous collection,);
   4. HbF quantitation by hemoglobin electrophoresis or HPLC in month 6 and 12 (by venous collection,);
   5. PAXgene tube storage (for those who have separately consented for future unspecified research) in month 3, 6, 9, or 12 (by venous collection);
   6. RDT (by fingerprick, only in those with temperature ≥37.5C or a history of subjective or objective fever in the preceding 24 hours).
4. Dispensing of 30-day supplies of:
   1. Study antimalarial prophylactic medication;
   2. Daily penicillin prophylaxis (for those < 5 years old).
5. ECG assessment (only in the first 20 residents of Homa Bay town allocated to monthly DP): 4-6 hours following the 3^rd^ (and final) dose of monthly DP (upon return 2 days later).

### 6.3.4 Final study visit

The final study visit procedures will be identical to those for routine study visits, except that prophylactic study agents will not be dispensed. The final study visit will be done at the month 12 visit or earlier if the participant is withdrawn earlier.

### 6.3.5 Early termination visit

If early termination occurs and the participant is willing, procedures will be identical to those for the final study visit. Participants who terminate the protocol early will not be replaced.

### 6.3.6 Acute-care visits through month 12

Unscheduled or acute-care visits will be arranged to be seen in the study clinic at HBCH within 1 working day of contact by the participant. These will be documented as acute-care visits in the study database. Study clinicians will evaluate participants and complete the following procedures:

1. Performance of structured medical history, including:
   1. Elicitation of chief complaint;
   2. Review of systems;
   3. Intake of non-study medications;
2. Performance of structured physical examination, including:
   1. Vital signs with weight;
   2. Anthropomorphic measurements;
   3. General physical survey.

Participants with temperature ≥37.5C, a history of subjective or objective fever in the preceding 24 hours, or other signs or symptoms potentially consistent with malaria will be tested for malaria parasites with an RDT. Those who test positive will be treated with either AL for uncomplicated malaria or referred for urgent evaluation to HBCH or another appropriate health care facility for potential severe malaria if adjudged as a concern by the study clinician.

### 6.3.7 Schedule of events table through month 12

| Procedures | Screening | Enrollment | Routine follow-up visits | Acute-care visits | Final visit |
| --- | --- | --- | --- | --- | --- |
| Hemoglobin electrophoresis/HPLC | X |  | X^1^ |  | X |
| Parent or LAR Consent | X |  |  |  |  |
| Medical History/Review of Systems |  | X | X | X | X |
| Physical Exam |  | X | X | X | X |
| Meningococcal Immunization (if needed) |  | X |  |  |  |
| Enrollment/maintenance in Kenyan NHIF |  | X | X |  |  |
| Dispense study medications |  | X | X |  |  |
| Study medication adherence assessment |  |  | X |  | X |
| AE/SUSAR Assessment |  |  | X |  | X |
| Study outcome assessment |  |  | X | X | X |
| Fingerprick hemoglobin concentration |  |  | X^5^ |  |  |
| Dried blood spot |  | X | X | X | X |
| CBC, creatinine, ALT | X |  | X^2^ |  | X |
| PAXgene venous blood |  |  | X^6^ |  |  |
| Malaria parasite RDT testing^3^ |  | X | X | X | X |
| ECG | X |  |  |  |  |
| ECG post-DP^4^ |  | X | X |  |  |

^1^ At the month-6 visit.

^2^ At the month-3, month-6, and month-9 visits.

^3^ If temperature ≥37.5C or a history of subjective or objective fever in the preceding 24 hours.

^4^ In the first 20 residents of Homa Bay town allocated to monthly DP only.

^5^ At the month 1, 2, 4, 5, 7, 8, 10, 11 visits.

^6^ Collected at only one routine follow up visit for participants who have consented separately for future unspecified research.

### 6.4 Concomitant Medications, Treatments, and Procedures

All children aged between 1 and 5 years, irrespective of study agent allocation, will receive daily penicillin prophylaxis with oral Penicillin V. The dosing is as follows:

| **Age (y)** | **No. of 125mg tabs BID** |
| --- | --- |
| 1-3 | 1 |
| >3-5 | 2 |

Other concomitant medications taken during study participation will be recorded on the case report forms (CRFs). For this protocol, a medication is defined as a manufactured pharmaceutical product that is obtained from a dispensary/pharmacy with or without a prescription from a medical provider. Therefore, medications to be reported in the CRF are concomitant prescription medications, over-the-counter medications, and non-prescription medications.

### 6.4.1 Precautionary medications, treatments, and procedures

Antifolate antibiotics that are routinely used to empirically treat suspected bacterial infections (such as co-trimoxazole) are similar to both Proguanil and to SP. Participants will be cautioned against the use of these medications during the study.

### 6.5 Prohibited Medications, Treatments, and Procedures

Prohibited medications include:

1. Antimalarial drugs not prescribed within the trial protocol;
2. Other drugs with antimalarial properties including co-trimoxazole; and
3. Medications known to prolong the QT interval (see Appendix L).

Participants who take prohibited medications will remain in the trial and will be included in the primary, intention-to-treat analysis, but excluded from the per-protocol analysis.

### 6.6 Rescue Medications, Treatments, and Procedures

Participants who are diagnosed with malaria by RDT will be treated with first-line treatment as recommended by the Kenya Ministry of Public Health and Sanitation National Guidelines for the Diagnosis, Treatment, and Prevention of Malaria.^18^ Artemether-lumefantrine (AL) is first-line treatment in Kenya for all age groups for uncomplicated falciparum malaria, ^18^ and will be used as treatment for malaria owing to any species of *Plasmodium* common in Kenya. The dosing of AL will be as follows:

| **Weight (kg)** | **No. of 20/120mg tabs BID for 3 days** |
| --- | --- |
| 5-14 | 1 |
| 15-24 | 2 |
| 25-34 | 3 |
| >34 | 4 |

Participants diagnosed with or suspected of severe malaria will be referred for admission to HBCH, and treated at the discretion of the treating provider.

### 6.7 Participant Access to Study Agent at Study Closure

Access to these medications are widely available and can be obtained by prescription from physicians providing care after the conclusion of the study, but will no longer be provided directly through the study protocol or by the study investigators.

## 7 ASSESSMENT OF SAFETY

### 7.1 Specification of Safety Parameters

Participants will have hemoglobin concentration measurements performed at screening and all followup visits. At screening, and month 3, 6, 9, and 12 visits, venous blood will be collected for testing by CBC. At month 1, 2, 4, 5, 7, 8, 10, and 11, fingerprick blood will be collected for point-of-care hemoglobin concentration measurement.

For all study participants, venous blood drawn at screening and month 3, 6, 9, and 12 visits, will also be used for creatinine and ALT measurements.

A subset of patients randomized to DP (20 children) will have serial electrocardiograms performed each month 4-6 hours following their 3rd dose of monthly DP.

Owing to the wide use of these antimalarials and their wide availability in Kenya, safety monitoring will focus on collection and reporting of SUSARS.

### 7.1.1 Definition of Suspected Unexpected Serious Adverse Reaction (SUSAR)

The definition of a SUSAR, as defined in ICH GCP, is: An adverse reaction, the nature or severity of which is not consistent with the applicable product information (e.g., for applicable product information include Investigator's Brochure for an unapproved investigational product or package insert/summary of product characteristics for an approved product).

### 7.1.2 Ascertainment of SUSARs

All SUSARs obtained via direct subject reporting and/or clinical staff observation will be captured within the SAE CRF and will be followed until resolution.

The study clinician will complete a SAE CRF. All SAEs will be reviewed by a Medical Safety Monitor (MSM) who is independent of the study team. The MSM will review the SAE CRF and gather any necessary further primary information from the study team during their review of the event. The MSM will classify the SAE as either a SUSAR or simply an SAE. The MSM will participate in an SAE adjudication committee with the PI and the Site Coordinator. This committee will convene on a recurring basis to review SAEs.

### 7.1.3 Time Period and Frequency for Event Assessment and Follow-Up

The occurrence of a SUSAR may come to the attention of study personnel during study visits and interviews of a study participant presenting for medical care, or upon review by a study monitor. Information regarding the potential SUSAR that will be collected includes SUSAR description, time of onset, clinician’s assessment of severity, relationship to study product (assessed only by those with the training and authority to make a diagnosis), and time of resolution/stabilization of the reaction. All SUSARs will be followed to adequate resolution.

### 7.2 Reporting Procedures

### 7.2.1 Reporting of SUSARs

Confirmed SUSARs (see Section 7.1.2) will be reported to DCRI, the DSMB, NHLBI, the Moi University IREC, the Duke University IRB, and the Kenya PPB as soon as possible and no later than 7 calendar days. This initial report will be updated with further information on final disposition of the SUSAR.

### 7.2.2 Reporting of Participant Deaths

All participant deaths will be reported to the DCRI, the DSMB, NHLBI, the Moi University IREC, the Duke University IRB, and the Kenya PPB irrespective of the SUSAR classification. Participant deaths will be reported to NHLBI within 7 calendar days.

### 7.2.3 Reporting of Unanticipated Problems

Unanticipated Problems (UPs), which are also known as Unanticipated Problems Involving Risks to Subjects or Others (UPIRTSO), may occur during the course of the study. When an event or condition has occurred that is considered a UP or UPIRTSO by the IRB or IREC, the event will be reported to NHLBI within 14 calendar days.

### 7.2.4 Summary of Event Reporting

| **Organization** | **SUSARs** | **Deaths**  **(non-SUSAR)** | **UP/UPIRTSO** |
| --- | --- | --- | --- |
| DCRI | X (7d) | X |  |
| Moi IREC | X (7d) | X |  |
| Duke IRB | X (7d) | X |  |
| DSMB | X (7d) | X |  |
| Kenya PPB | X (7d) | X |  |
| NHLBI | X (15d) | X (7d) | X (14d) |

### 7.3 Study Halting Rules

All SUSARs and non-SUSAR participant deaths will be reported promptly to the DSMB (see Section 7.2.4). Study stopping guidelines are are designed to detect an imbalance of participant deaths in either intervention arm (monthly SP-AQ or monthly DP) compared to the standard of care (SOC) arm (daily Proguanil). For this purpose, sequential boundaries will be used to continuously monitor the rate of participant deaths in each treatment arm (monthly SP-AQ or monthly DP) compared to the standard-of-care SOC arm (daily Proguanil). Accrual into a treatment arm will be considered for stopping if an excessive number of deaths is seen in that treatment arm compared to the SOC arm, that is, if the difference in the number of deaths between that treatment arm and SOC arm is equal to or exceeds *b_n_* out of *n* enrolled participants in the treatment arm (see table below). This is based on a Pocock-type stopping boundary that yields the probability of crossing the boundary when the difference in the rate of death is equal to or exceeds 5%.

| ***n*** | 2-5 | 6-14 | 15-24 | 25-36 | 37-48 | 49-61 | 62-74 | 75-82 |
| --- | --- | --- | --- | --- | --- | --- | --- | --- |
| ***b_n_*** | 2 | 3 | 4 | 5 | 6 | 7 | 8 | 9 |

Assuming a maximum planned sample size of 82 in each of the three study arms and a desired probability of stopping owing to chance of 0.10, a treatment arm (SP-AQ or DP) will be considered for stopping if the difference in the number of deaths between the treatment arm and the SOC arm is equal to or exceeds *b_n_* out of *n* enrolled participants in each of the two arms.^55^

The stopping rules assume an equal number of participants in the treatment and SOC arms being compared when a death occurs. If the numbers within the arms are imbalanced, the confidence interval should be verified by the study statistician prior to the final decision to stop a treatment arm. We should further consider the timing of events through Kaplan-Meier plots before the final decision is made.

In addition, the DSMB will review in its regular meetings these data and all SAEs. Using these collective data or other concerns, the DSMB has the authority to recommend halting the study. If such concerns arise, the DSMB will convene an ad hoc meeting by teleconference or in writing as soon as possible. As a result of this ad hoc meeting, the DSMB will provide recommendations for proceeding with the study or halting the study to the NHLBI, who can follow or not follow the recommendations.

### 7.4 Safety Oversight

Safety oversight will be under the direction of a DSMB organized by NHLBI composed of individuals with the appropriate expertise, including a 1) pediatric hematologist, 2) statistician, 3) bioethicist, and at least 1 other. At least one member will have experience with clinical care or research in Kenya. The DSMB will meet at least semiannually to assess safety and efficacy data on each arm of the study. The DSMB will operate under the rules of an approved charter that will be written and reviewed at the organizational meeting of the DSMB. At that time, each data element that the DSMB needs to assess will be clearly defined.

## 8 CLINICAL MONITORING

GCP guidelines will be followed when conducting routine monitoring for this study, to ensure protocol compliance, patient safety, and data integrity. Experienced clinical research associates will be assigned to conduct these visits. Specific details related to the monitoring can be found in the study’s monitoring plan described in the study MOP. Key areas that will be reviewed include informed consent, eligibility criteria, endpoint components, SUSARs, and drug accountability.

## 9 STATISTICAL CONSIDERATIONS

### 9.1 Statistical and Analytical Plans

This section provides a general description of the statistical methods to be used in analyzing both effectiveness and safety data. The key statistical issues or considerations will be addressed. A more detailed statistical analysis plan (SAP) will be provided in a separate document to be included in the study MOP.

Unless otherwise specified, all statistical tests will be two-sided with a significance level of 0.05. Summary statistics will be provided for all study variables with descriptive statistics (number of observations, mean, standard deviation (SD), median, 25^th^ percentile, 50^th^ percentile, minimum, and maximum) for numerical (or continuous) variables and with frequency and percentage for non-missing categorical variables.

All statistical analysis will be conducted using SAS version 9.4 or higher (SAS Institute Inc., Cary, NC, USA).

### 9.2 Statistical Hypotheses

The primary outcome of the study is clinical malaria, which will be expressed as an incidence rate (IR; the number of episodes per patient-year at risk of malaria) and will be evaluated through the 12-month follow-up visit for effectiveness.

The null hypothesis for the primary endpoint is that there is no treatment difference between each of the experimental arms (monthly SP-AQ and monthly DP) compared to the standard-of-care (SOC) arm (daily Proguanil) from randomization to 12 months. The alternative hypothesis is that each of the experimental arms has a better treatment response (i.e., lower IR of malaria) compared to the SOC arm through 12 months of treatment and follow-up.

Let IR**_SP_** and IR**_DP_** be the expected number of episodes per patient-year at risk (incidence rate) through 12-months for SP-AQ and DP arms, respectively, and IR**_C_** the rate for the SOC arm. The null and alternative hypotheses are as follows:

H_O_: IR**_SP_** = IR**_C_**  Vs. H_A_: IR**_SP_** ≠ IR**_C_,** and

H_O_: IR**_DP_** = IR**_C_**  Vs. H_A_: IR**_DP_** ≠ IR**_C_**.

### 9.3 Analysis Datasets

We will define three populations, including an as-treated (AT) population of all treated participants according to the treatment administered, an intention-to-treat (ITT) population of all randomized patients irrespective of confirmed receipt or administration of study drugs, and an according-to-protocol (ATP) population, consisting of those who completed 12 months of follow-up and confirmed receipt of study drugs at routine follow-up visits. The AT population will be used for the primary analysis.

### 9.4 Description of Statistical Methods

### 9.4.1 General approach

Incidence rates for each experimental arm in the AT population will be compared to the control group using a **generalized** regression model that allows for interdependence between **multiple** episodes within the same participant that is unadjusted for potential covariates. **Models considered for the count data will include the Poisson, negative-binomial and zero-inflated models with the final model determined by the smaller residual** **deviance.** Similar analyses for the ITT and ATP population will also be computed, as well as adjusted models which incorporate potential covariates, including age, baseline risk and bednet use. Each regression model will provide incidence rate ratios with 95% confidence intervals for each experimental treatment group compared to the control arm. A two-sided p-value < 0.05 will be considered statistically significant unless otherwise stated.

### 9.4.2 Analysis of the primary efficacy endpoint

The primary outcome of malaria will be expressed as an IR, or the number of episodes per patient-year at risk through 12-months. IRs will be computed after subtracting two weeks from the time at risk for each episode, in order to avoid counting an episode more than once.

IRs of malaria will be primarily compared between each experimental treatment group and the control arm using a **generalized** regression model. The model will be unadjusted and will utilize the AT population. The regression model will allow interdependence between episodes within the same individual (i.e., clustering by subject). This will create robust standard errors for the parameter estimates, and also allow the use of actual follow-up time as the offset variable

The treatment difference for each hypothesis test will be tested using the contrast between treatment groups under the regression model. These differences will be presented as incidence rate ratios per patient-year at risk with 95% confidence intervals for each experimental treatment group compared to the control arm. In order to control the nominal type-I error rate (α=0.05), each test and corresponding confidence interval will use an α=0.0269 per Dunnett’s test for multiple comparisons to the same control arm.^56^

#### 9.4.2.1 Special data considerations

Subjects will be followed for up to 12 months for both the primary and secondary endpoints. The sample size allows up to 20% of the subjects to be lost to follow-up and still maintain adequate statistical power. Methods to reduce loss to follow-up will be employed by the study team, including telephone calls and, if necessary, home visits; however, not all subjects will be followed for the entire study period. Subjects may have missing data during some of the follow-up time points; however, the generalized linear models used for the longitudinal primary and secondary endpoints will allow for any data collected to be utilized for analysis. Thus, no imputation of endpoint data will be done.

### 9.4.3 Analysis of the secondary endpoints

Secondary endpoints (defined in Section 3.3) will also be compared between arms and are:

1. Severe malaria;
2. Hospitalization for malaria;
3. LM-positive malaria (unconfirmed by RDT);
4. Unconfirmed malaria (receipt of antimalarials unconfirmed by any diagnostic test);
5. Fatal malaria;
6. Asymptomatic parasitization;
7. Painful events;
8. Dactylitis;
9. Transfusions;
10. Acute chest syndrome;
11. All-cause hospitalization;
12. All-cause deaths;
13. Molecular markers of malaria parasite drug resistance.

The secondary outcomes will **each be expressed as a dichotomous response (yes/no) during follow-up and compared between treatment arms using a logistic regression model to estimate the unadjusted relative risk. Additionally, for outcomes in which more than one event is possible, event rates *may* be compared using a generalized regression model similar to that used for the primary outcome. Since the event rates are likely to be very small, the following composite outcomes will also be compared:** (1) dactylitis or painful events; (2) receipt of blood products, ACS, hospitalization, or death; and (3) any secondary outcome.

### 9.4.4 Data and safety analyses

The DSMB is an independent committee that oversees the safety of research subjects. It is anticipated that the DSMB will meet every 6 months to review the accumulating data. Prior to each meeting, the DCRI will conduct any requested statistical analyses and prepare a summary report along with the following information: patient enrollment reports, **study status, demographics, serious adverse events, laboratory results, and counts of primary and secondary outcomes.** The extracted data files and analysis programs for each DSMB report will be archived and maintained at the DCRI for the life of the study.

The DSMB will weigh any trade-offs between short-term versus long-term results. The DSMB will play a valuable role in advising the study leadership on the relevance of advances in the diagnosis and treatment of patients with SCA and malaria. The DSMB would be asked to offer proper perspective on any therapeutic or diagnostic testing advances that may occur during the course of the trial. If protocol modifications are warranted, close consultation among the DSMB, the NHLBI staff and the study leadership will be required. A separate DSMB charter will outline the operating guidelines for the committee, and the protocol for evaluation of data. The charter will be created prior to patient randomization and agreed upon during the initial meeting of the DSMB.

Minutes of all DSMB meetings will be prepared and distributed to committee members. DSMB reports will be distributed by the study sponsor to study investigators, who will then report these to the overseeing ethical review committees, including Moi University IREC and the Duke University IRB.

### 9.4.5 Adherence and retention analyses

Adherence to study medications will be assessed at each routine follow-up visit using a structured query. Adherence assessments will collect participant/guardian-reported adherence as both continuous and binary estimates of adherence, and include qualitative queries to address underlying reasons for suboptimal adherence.

### 9.4.6 Baseline descriptive statistics

At the time of randomization, we will compare between allocation arms:

- Age (years, continuous);
- Hemoglobin (g/dL, continuous) and other CBC red cell indices;
- Anthropomorphic measurements (continuous);
- Current use of malaria prophylaxis (dichotomous);
- Current use of penicillin prophylaxis (dichotomous);
- Current use of hydroxyurea (dichotomous);
- History of transfusions (dichotomous);
- HbF quantitation (continuous);
- All-cause hospitalizations in the prior 12 months

### 9.4.7 Planned interim analyses

For ethical reasons, an interim examination of key safety data will be performed when the study has accrued approximately ½ of the estimated total person-time. The primary objective of this analysis will be to evaluate the accumulated data for high frequency of negative clinical outcomes in either of the three randomized arms. In addition, the periodic monitoring will review the standard-of-care arm event rates, patient recruitment, compliance with the study protocol, status of data collection, and other factors that reflect the overall progress, data quality, and integrity of the study. The results of the interim analysis and monitoring reports will be carefully and confidentially reviewed by the DSMB. The DSMB will then give their recommendations for continuing or stopping the study to the PIs.

We have no plans for a formal analysis for either efficacy or futility, owing to both the short 12 month duration of the study as well as the provision of malaria chemoprevention to all participants.

### 9.4.8 Additional sub-group analyses

Malaria incidence rates, painful crises and dactylitis will also be analyzed by including baseline covariates into the regression models including, age, bednet use and their receipt of hydroxyurea at baseline. Both unadjusted and adjusted comparisons between the two experimental arms will be done.

### 9.4.9 Tabulation of individual response data

Individual participant data will be listed by measure and time point.

### 9.4.10 Exploratory analyses

We will explore the relationship between HbF quantitation and neutrophil counts and the incidence of parasitological and hematological outcomes.

### 9.5 Sample Size

Expected reductions in malaria incidence are imprecise of intermittent chemoprevention with SP-AQ or DP. In three West African studies, monthly SP-AQ in children aged up to 5 years reduced the incidence of malaria (compared to placebo) by 70-83%.^12,13,34^ Therefore, we anticipate more modest reductions in malaria incidence with SP-AQ owing to resistance to SP AQ in East Africa, the use of daily Proguanil as the comparator, and the inclusion of children up to 10 years. For DP, reductions relative to placebo of 45% (relative to placebo) in Ugandan children^35^ and 77% in Burkinabé children have been reported.^34^ Given the use of an active comparator (daily Proguanil), the higher prevalence of SP resistance in Kenya (compared with Burkina), and the inclusion of children aged up to 10 years, we anticipate more modest reductions with either study treatment, and a protective efficacy of SP-AQ that is approximately half of that of DP.

To estimate sample sizes, we used as baseline incidence data the number of episodes of malaria in calendar year of 2011 by children enrolled in the RTS,S/AS01 vaccine trial in Siaya, Kenya.^57^ Siaya is located on the opposite shore of Lake Victoria from Homa Bay, and they have similar degrees of malaria risk.^24^ The Siaya cohort comprised 1,276 children aged up to 17m who received either three doses of RTS,S/AS01 or a comparator vaccine; follow-up was passive and clinical malaria was defined as an illness with temperature ≥37.5C and *P. falciparum* parasites at a density >5,000 parasites/μL. In Siaya, the overall annual incidence was 3.7 episodes per child, and the overall vaccine efficacy in children was 43%. See **Figure 2A** for the distribution of the number of clinical malaria episodes for all Siaya children in the year 2011.

To compute sample sizes for the EPiTOMISE trial, we assumed a similar expected distribution of episodes for the standard-of-care arm (daily Proguanil); that the monthly SP-AQ and monthly DP arms would also have similar distributions of episodes, but shifted left by effect factors; and that one treatment arm (DP) would have an effect size of twice that of the other (SP-AQ). We assumed that the test for each experimental arm would be made at the 0.0269 level in order to preserve a nominal alpha level of 0.05.^56^ Assuming 65 patients enrolled in each of three arms and followed for 12 months, we computed the power of each arm as a function of the expected reductions in episodes (**Figure 2B**). Power calculations were made using Compaq Visual Fortran,^58^ and were based on 100,000 simulations per point on the curve. By enrolling 65 patients in each arm, we expect to have:

**Figure 2**. A) Distribution of clinical malaria events in 1,276 children enrolled in the RTS,S/AS01 vaccine trial in Siaya, Kenya, during the 2011 calendar year. B) Estimated power (y-axis) to detect reductions in malaria incidence at either the full (dotted) or half (dashed) listed degrees on the x-axis, assuming 65 patients enrolled in each of 3 arms.

1. >90% power to detect a reduction of 40% in the DP arm;
2. ~40% power to detect a reduction of 20% in the Proguanil arm; and

| **Counts of Painful Events** | **Observed**  **Counts** | **Observed Fraction** | **Predicted Fraction** |
| --- | --- | --- | --- |
| 0 | 22 | 0.227 | 0.204 |
| 1 | 15 | 0.155 | 0.169 |
| 2 or 3 | 19 | 0.196 | 0.243 |
| 4+ | 41 | 0.423 | 0.384 |

1. >90% power to detect a reduction of 40% in either arm.

Given the reported efficacies of DP and SP-AQ in non-SCA children cited above, these expected reductions are reasonable when compared with an active comparator (Proguanil). Allowing for a drop-out rate of 20%, we propose to enroll 246 children and randomize 1:1:1 to the treatment arms.

**Table 2**. Number of observed and predicted painful events. Observed counts are from BABY-HUG study, and predicted counts computed as described in Section 9.5. The chi-square goodness of fit is 2.22 for 2 degrees of freedom, p = 0.330.

The main secondary outcome is the incidence of painful events. The sample size for this aim is fixed at 246 overall, or 65 participants in each arm allowing for 20% participant loss. Therefore, using this pre-specified size, we estimated the power of the EPiTOMISE study to detect various percent reductions in painful events. To do so, we used as baseline data the distribution of painful events (by the same definition proposed here) in the BABY-HUG study, a phase 3 multicenter clinical trial of hydroxyurea in infants with SCA in the USA (**Table 2**).^53,59^ For this endpoint we did not have the complete distribution as we did for the primary endpoint (**Figure 2A**). However, we found that a negative binomial distribution as described by Bartko*^60^* provided an adequate fit:

$$P[x=j] = \left(\begin{matrix} N + j - 1 \\ N - 1 \end{matrix}\right) p^{j} (1 + p)^{N + j} .$$

Table 3 shows the fit to the observed data. We then used the predicted distribution based on the negative binomial as the expected distribution for the placebo arm and computed power estimates as above in order to estimate study power as a function of possible percent reductions in painful events in either treatment arm (**Figure 3**). Using this approach, we will have 80% power to detect a reduction of 35% in either treatment arm relative to the Proguanil arm.

**Figure 3**. Estimated power as a function of possible percent reductions in painful events. Distribution of painful events extracted from participants receiving placebo in the BABY-HUG study. Power estimates assume 65 patients in each treatment arm (Section 9.5).

### 9.6 Measures to Minimize Bias

### 9.6.1 Enrollment/Randomization

At enrollment, participants will be randomized in a 1:1:1 ratio to one of the study arms: 1) daily Proguanil (standard-of-care), 2) monthly SP-AQ, or 3) monthly DP. After screening and informed consent is obtained, the site-investigator or research assistant will electronically randomize a patient to a study group intervention using the REDCap database (web-based secure database application maintained by the Duke Investigators).  The Duke Statistical Investigator (Department of Biostatistics and Bioinformatics, DUMC, Durham, NC) will provide the randomization scheme that will be used within the REDCap database to randomly assign patients to one of the three treatment groups.  A block randomization scheme will be used in order to preserve treatment group balance within the site.  The block size will be undisclosed.  Each patient who qualifies for entry into the study will be assigned a unique study number in chronological order within the participating site (i.e., site ID + patient ID).

This study will be open-label, and therefore there will be no masking of study personnel or participants to the treatment allocation. In order to reduce bias in the recording of the primary outcome, objective measurement of malaria parasitemia by commercial RDT will be required as an endpoint criterion.

## 10 SOURCE DOCUMENTS AND ACCESS TO SOURCE DATA/DOCUMENTS

Source documents include all recordings of observations or notations of clinical activities and all reports and records necessary for the evaluation and reconstruction of the clinical study.

Whenever possible, the original recording of an observation will be retained as the source document; however, a photocopy is acceptable provided that it is a clear, legible, and exact duplication of the original document.

Investigators will maintain information that corroborate data collected on the electronic CRF (eCRF). The following information will be maintained and made available as required by study monitors and/or inspectors:

1. Medical history/physical condition of the study subject before involvement in the study sufficient to verify protocol entry criteria.
2. Documentation that consent was obtained for the subject’s participation in the study.
3. Dated and signed notes for each subject visit, including results of examinations.
4. Notations on abnormal laboratory results and their resolution.
5. Dated printouts or reports of special assessments (e.g., ECG reports).
6. Description of SUSARS and follow-up (minimally: event description, severity, onset date, duration, relation to study drug/device, outcome, and treatment for SUSAR).
7. Notes regarding concomitant medications taken during the study (including start and stop dates).
8. Subject’s condition upon completion of or withdrawal from the study.

## 11 QUALITY ASSURANCE AND QUALITY CONTROL

Quality assurance personnel may conduct audits at the study sites. Audits will include, but not be limited to, examination of the audit trail of data handling and processes, standard operating procedures, drug supply, presence of required documents, the informed consent process, and comparison of eCRFs with source documents. The investigators agree to accommodate and participate in audits conducted at a reasonable time and in a reasonable manner.

Regulatory authorities from Kenya or elsewhere may also inspect the study site during or after the study. The study site should contact the study leadership immediately if this occurs and must fully cooperate with governmental audits conducted at a reasonable time and in a reasonable manner.

The study leadership and study site will provide access to all trial related sites, source data/documents, and reports for the purpose of monitoring and auditing by the sponsor, and inspection by local and regulatory authorities.

## 12 ETHICS/PROTECTION OF HUMAN SUBJECTS

### 12.1 Ethical Standard

By signing this protocol, the PIs agree to conduct the study in compliance with the protocol; instructions given by the DCRI; International Council on Harmonisation guidelines on Good Clinical Practice (ICH E6, the principles of which have their origin in the Declaration of Helsinki); the Guidelines for Conduct of Clinical Trials In Kenya, Pharmacy and Poisons Board, September 2016; and all other applicable laws, rules, and regulations relating to the conduct of the clinical study.

### 12.2 Institutional Review Board

Before implementing this study, the protocol, the proposed informed consent form and other information to subjects, must be reviewed by the Moi University IREC and the Duke University IRB. A signed and dated statement that the protocol and informed consent have been approved by these ethical review boards will be given to the study investigators before study initiation. Any amendments to the protocol, other than administrative ones, must be approved by the Moi IREC and Duke IRB according to their procedures. All changes to consent form will be IRB-approved; a determination will be made subsequent to this regarding whether previously consented participants need to be re-consented.

### 12.3 Informed Consent Process

### 12.3.1 Consent and other informational documents provided to participants

The PIs have both ethical and legal responsibility to ensure that each subject being considered for inclusion in this study is given a full explanation of the study. Written informed consent will be obtained from all subjects (or their guardian or legally authorized representative [LAR]) before any study-related procedures (including testing for the purposes of determining final study eligibility during screening) are performed or given.

Written informed consent will be documented on an informed consent form (ICF) approved by the same IRBs responsible for approval of this protocol. The ICF will conform to FDA regulations in 21 CFR Part 50 and to the institutional requirements for informed consent and applicable regulations.

The ICF will be reviewed with the prospective study subject and his or her LAR, and the investigator or qualified designee will be available to answer questions regarding procedures, risks, and alternatives.

Specific consent is required for blood samples to be stored for future studies in SCA, malaria, and other infections in children with SCA. Subjects who do not consent to such storage of samples will be asked to sign the ICF separately to document their refusal of consent for future use of these samples for this purpose. Participation in the study is not contingent upon a subject’s agreement to provide these blood samples.

Once the appropriate essential information has been provided to the subject and fully explained by the investigators or qualified designee, and it is felt that the subject understands the implications of participating, the subject and the investigator or designee will sign and date the IRB-approved written ICF. The subject will receive a copy of the signed ICF. The original signed and dated ICF will be kept in the site’s regulatory file. Documentation of the subject’s informed consent for and participation in this trial will be noted in the subject’s medical record.

If the parent or guardian is unable to read or write, their fingerprint will substitute for a signature, and a signature from a witness to the informed consent discussion will be obtained.

The subject or his or her LAR will be informed in a timely manner if new information becomes available that may be relevant to the subject’s willingness to continue participation in the trial. The communication of this information to the subject will be documented.

### 12.3.2 Consent procedures and documentation

Informed consent will be conducted in either English or Kiswahili or Dholuo, as appropriate, and a translator will be used if necessary. The informed consent form will describe the purpose of the study, all the procedures involved, and the risks and benefits of participation. Study physicians will ask parents/guardians of study participants to summarize the study and explain the reasons why they want to participate. The consent forms will be translated into Kiswahili and Dholuo and back-translated into English to check for any loss or change of meaning. Either a signature or a thumbprint (for parents/guardians who cannot read) will be acceptable to confirm informed consent for participation in the study.

The participant will sign the informed consent document prior to any procedures being done specifically for the study. The participants may withdraw consent at any time throughout the course of the trial. A copy of the informed consent document will be given to the participants for their records. The rights and welfare of the participants will be protected by emphasizing to them that the quality of their medical care will not be adversely affected if they decline to participate in this study.

### 12.4 Participant and Data Confidentiality

Participant confidentiality is strictly held in trust by the participating investigators, their staff, and the sponsor(s) and their agents. This confidentiality is extended to cover testing of biological samples and genetic tests in addition to the clinical information relating to participants. Therefore, the study protocol, documentation, data, and all other information generated will be held in strict confidence. No information concerning the study or the data will be released to any unauthorized third party without prior written approval of the sponsor.

The study monitor, other authorized representatives of the sponsor, representatives of the IRB may inspect all documents and records required to be maintained by the investigator, including but not limited to, medical records (office, clinic, or hospital) and pharmacy records for the participants in this study. The clinical study site will permit access to such records.

The study participant’s contact information will be securely stored at each clinical site for internal use during the study. At the end of the study, all records will continue to be kept in a secure location for at least 10 years from the conclusion of the study, in accordance with guidelines from the Kenyan Pharmacy and Poisons Board.

Study participant research data, which is for purposes of statistical analysis and scientific reporting, will be transmitted to and stored at the DCRI. This will not include the participant’s contact or other identifying information. Rather, individual participants and their research data will be identified by a unique study identification number. The study data entry and study management systems used by clinical sites and by DCRI research staff will be secured and password protected. At the end of the study, all study databases will be de-identified and archived at the DCRI.

### 12.4.1 Research use of stored human samples, specimens or data

*Intended use*. Samples and data collected under this protocol may be used to study SCA, malaria, or co-existing infectious diseases in children. No human genetic testing will be performed except to test for mediators of SCA or malaria severity.

*Storage*. Access to stored samples will be limited using a locked laboratory. Samples and data will be stored using codes assigned by the investigators. Data will be kept in password-protected computers. Only investigators will have access to the samples and data.

*Tracking*. Data will be tracked using a clinical trial informatics system.

*Disposition at the completion of the study*. The stored PAXgene tubes and DBS will be maintained in a locked laboratory at Duke University. Study participants who request destruction of samples will be notified of compliance with such request and all supporting details will be maintained for tracking.

### 12.5 Potential Future Use of Stored Specimens

Data collected for this study will be analyzed and stored at the DCRI, in collaboration with co-investigators from Moi University. After the study is completed, the de-identified, archived data will be transmitted to and stored at the DCRI, under the supervision of Dr. Taylor, for potential use by other researchers, including those outside of the study. Permission to transmit data to the DCRI and allow use for future research will be included in the informed consent.

With the participant’s approval and as approved by local IRBs, de-identified DBS and PAXgene tubes will be stored at the Dr. Taylor’s laboratory at Duke University with the same goal as the sharing of data with the DCRI. These samples could be used for research into the causes of SCA or malaria, its complications and other conditions for which individuals with SCA are at increased risk, and to improve treatment. These samples may be shared by request with investigators outside of Duke or Moi Universities, but only for the same purposes as those described above. The DCRI will also be provided with a code-link that will allow linking the biological specimens with the phenotypic data from each participant, maintaining the masking of the identity of the participant.

During the conduct of the study, an individual participant can choose to withdraw consent to have biological specimens stored for future research. When the study is completed, access to study data and/or samples will be provided through the DCRI.

## 13 DATA HANDLING AND RECORD KEEPING

### 13.1 Data Collection and Management Responsibilities

Study sites will transcribe subject source data into eCRFs using a computerized electronic data capture (EDC) system. The EDC system has controls that enable compliance with all relevant aspects of GCP. Edit checks, electronic queries, and audit trails are built into the system to ensure accurate and complete data collection. Data will be transmitted via the Internet from the study site to servers hosted by DCRI. The EDC employs sophisticated encryption mechanisms to ensure security and confidentiality, including password protection that will prevent unauthorized access during transmission.

Subject data will be entered in the eCRF at the time of or as soon as possible after the subject’s visit or the availability of test results. All completed eCRFs will be transmitted in real-time as the study proceeds, or uploaded in batch fashion in the event of network interruptions.

### 13.2 Study Records Retention

The site investigator will maintain the records of drug disposition, copies of final eCRFs, worksheets, and all other study-specific documentation (e.g., study file notebooks or documentation worksheet) for at least 10 years from the end of the study, in accord with the Guidelines for Conduct of Clinical Trials In Kenya, PPB, September 2016. In accord with these guidelines, these records will be archived in Kenya. To avoid error, the investigator will contact the sponsor or a sponsor designee, as well as the Kenya Pharmacy and Poisons Board, before the destruction of any records pertaining to the study to ensure they no longer need to be retained. In addition, if the site investigator plans to leave the institution, the investigator agrees to contact the sponsor or a sponsor designee so that arrangements can be made for the transfer of records.

### 13.3 Protocol Deviations

A protocol deviation is any noncompliance with the clinical trial protocol, GCP, or MOP requirements. The noncompliance may be either on the part of the participant, the investigator, or the study site staff. As a result of deviations, corrective actions are to be developed by the site and implemented promptly.

It is the responsibility of the site to use continuous vigilance to identify and report deviations within 5 working days of identification of the protocol deviation, or within 5 working days of the scheduled protocol-required activity. All deviations must be addressed in study source documents and reported to DCRI. Protocol deviations must be sent to the Moi University IREC and the Duke University IRB per their guidelines. The site PI/study staff is responsible for knowing and adhering to their IRB requirements

### 13.4 Publication and Data Sharing Policy

This study will comply with the NIH Public Access Policy, which ensures that the public has access to the published results of NIH-funded research. It requires scientists to submit final peer-reviewed journal manuscripts that arise from NIH funds to the digital archive PubMed Central upon acceptance for publication.

The International Committee of Medical Journal Editors (ICMJE) member journals have adopted a clinical trials registration policy as a condition for publication. Therefore this study will be registered at ClinicalTrials.gov, which is sponsored by the National Library of Medicine, and the Pan African Clinical Trial Registry ([www.pactr.org)](http://www.pactr.org)).

Parasite gene sequences generated for drug resistance genotyping using next-generation sequencing platforms will be deposited in the National Library of Medicine’s Short Read Archive at the time of publication of genotyping results or within 6 months of preliminary analysis of these data, whichever occurs first.

### 13.5 Data and Safety Monitoring Plan

Individual elements within this protocol collectively constitute the Data and Safety Monitoring Plan (DSMP) for the EPiTOMISE study. This DSMP comprises:

1. Description of study monitoring (Section 8);
2. Collection, reporting, and management of safety events (Section 7);
3. Risks and benefits to participants (Section 1.3);
4. Conflicts of interest among study personnel (Section 15);
5. Data collection and analysis (Sections 9 and 13);
6. Interim analyses (Section 9.4.7);
7. Study halting rules (Section 7.4); and
8. DSMB plan (Section 9.4.4).

## 14 STUDY ADMINISTRATION

### 14.1 Coordinating Center

The Coordinating Center (CC) will be at the DCRI. The CC functions as a clinical trial center and is responsible for all aspects of conducting this trial, including: clinical operations; oversight of all committees and working groups; development of the protocol and all amendments; quality control; site reimbursement; monitoring of study progress; and leadership in data analysis, presentations, and publications. Clinical Operations is the critical functional component of the CC, and will provide project management; development and preparation of study materials; site management; education of all site-based personnel on the rationale, design, and execution of EPiTOMISE, and assistance with preparation of manuscripts and publications.

CC staff will develop and implement educational and training plans, communication initiatives including phone and email contact, and conference calls. The CC staff will collaborate with the study site personnel to ensure their understanding of the protocol, the operationalization of the protocol, and the successful identification of eligible patients for screening and enrollment.

## 15 CONFLICT OF INTEREST POLICY

The independence of this study from any actual or perceived influence, such as by the pharmaceutical industry, is critical. Therefore any actual conflict of interest of persons who have a role in the design, conduct, analysis, publication, or any aspect of this trial will be disclosed and managed. Furthermore, persons who have a perceived conflict of interest will be required to have such conflicts managed in a way that is appropriate to their participation in the trial. The DCRI in conjunction with the NHLBI has established policies and procedures for all study group members to disclose all conflicts of interest and will establish a mechanism for the management of all reported dualities of interest.

## 16 LITERATURE REFERENCES

1 Piel, F. B. *et al.* Global epidemiology of sickle haemoglobin in neonates: a contemporary geostatistical model-based map and population estimates. *Lancet* **381**, 142-151, doi:10.1016/S0140-6736(12)61229-X (2013).

2 Piel, F. B., Hay, S. I., Gupta, S., Weatherall, D. J. & Williams, T. N. Global burden of sickle cell anaemia in children under five, 2010-2050: modelling based on demographics, excess mortality, and interventions. *PLoS medicine* **10**, e1001484, doi:10.1371/journal.pmed.1001484 (2013).

3 Aidoo, M. *et al.* Protective effects of the sickle cell gene against malaria morbidity and mortality. *Lancet* **359**, 1311-1312, doi:10.1016/S0140-6736(02)08273-9 (2002).

4 WHO. *World Malaria Report 2014*, <<http://www.who.int/malaria/publications/world_malaria_report_2014/en/>> (2014).

5 McAuley, C. F. *et al.* High mortality from Plasmodium falciparum malaria in children living with sickle cell anemia on the coast of Kenya. *Blood* **116**, 1663-1668, doi:10.1182/blood-2010-01-265249 (2010).

6 Makani, J. *et al.* Malaria in patients with sickle cell anemia: burden, risk factors, and outcome at the outpatient clinic and during hospitalization. *Blood* **115**, 215-220, doi:10.1182/blood-2009-07-233528 (2010).

7 Konotey-Ahulu, F. I. Malaria and sickle-cell disease. *British medical journal* **2**, 710-711 (1971).

8 Fleming, A. F. The presentation, management and prevention of crisis in sickle cell disease in Africa. *Blood reviews* **3**, 18-28 (1989).

9 Komba, A. N. *et al.* Malaria as a cause of morbidity and mortality in children with homozygous sickle cell disease on the coast of Kenya. *Clinical infectious diseases : an official publication of the Infectious Diseases Society of America* **49**, 216-222, doi:10.1086/599834 (2009).

10 Aneni, E. C., Hamer, D. H. & Gill, C. J. Systematic review of current and emerging strategies for reducing morbidity from malaria in sickle cell disease. *Tropical medicine & international health : TM & IH* **18**, 313-327, doi:10.1111/tmi.12056 (2013).

11 Aponte, J. J. *et al.* Efficacy and safety of intermittent preventive treatment with sulfadoxine-pyrimethamine for malaria in African infants: a pooled analysis of six randomised, placebo-controlled trials. *Lancet* **374**, 1533-1542, doi:10.1016/S0140-6736(09)61258-7 (2009).

12 Konate, A. T. *et al.* Intermittent preventive treatment of malaria provides substantial protection against malaria in children already protected by an insecticide-treated bednet in Burkina Faso: a randomised, double-blind, placebo-controlled trial. *PLoS medicine* **8**, e1000408, doi:10.1371/journal.pmed.1000408 (2011).

13 Dicko, A. *et al.* Intermittent preventive treatment of malaria provides substantial protection against malaria in children already protected by an insecticide-treated bednet in Mali: a randomised, double-blind, placebo-controlled trial. *PLoS medicine* **8**, e1000407, doi:10.1371/journal.pmed.1000407 (2011).

14 Organization, W. H. (2010).

15 Organization, W. H. WHO Policy Recommendation: Seasonal Malaria Chemoprevention (SMC) for *Plasmodium falciparum* malaria control in highly seasonal transmission areas of the Sahel sub-region in Africa. (2012).

16 Nwokolo, C. *et al.* Mefloquine versus proguanil in short-term malaria chemoprophylaxis in sickle cell anaemia. *Clin Drug Invest* **21**, 537-544, doi:Doi 10.2165/00044011-200121080-00002 (2001).

17 Eke, F. U. & Anochie, I. Effects of pyrimethamine versus proguanil in malarial chemoprophylaxis in children with sickle cell disease: a randomized, placebo-controlled, open-label study. *Current Therapeutic Research* **64**, 616 (2003).

18 Vol. 3 (ed Ministry of Public Health and Sanitation & Ministry of Medical Services) (2010).

19 Nakibuuka, V., Ndeezi, G., Nakiboneka, D., Ndugwa, C. M. & Tumwine, J. K. Presumptive treatment with sulphadoxine-pyrimethamine versus weekly chloroquine for malaria prophylaxis in children with sickle cell anaemia in Uganda: a randomized controlled trial. *Malaria journal* **8**, 237, doi:10.1186/1475-2875-8-237 (2009).

20 Diop, S. *et al.* Sickle-cell disease and malaria: evaluation of seasonal intermittent preventive treatment with sulfadoxine-pyrimethamine in Senegalese patients-a randomized placebo-controlled trial. *Annals of hematology* **90**, 23-27, doi:10.1007/s00277-010-1040-z (2011).

21 Olaosebikan, R. *et al.* A randomized trial to compare the safety, tolerability, and effectiveness of three antimalarial regimens for the prevention of malaria in Nigerian patients with sickle-cell disease. *in preparation* (2014).

22 Warley, M. A. *et al.* Chemoprophylaxis of Homozygous Sicklers with Antimalarials and Long-Acting Penicillin. *British medical journal* **2**, 86-88 (1965).

23 Project, M. A. *Malaria Atlas Project*, <<http://www.map.ox.ac.uk/browse-resources/>> (2014).

24 Okiro, E. A. *et al.* Malaria paediatric hospitalization between 1999 and 2008 across Kenya. *BMC medicine* **7**, 75, doi:10.1186/1741-7015-7-75 (2009).

25 Onchiri, F. M. *et al.* Frequency and correlates of malaria over-treatment in areas of differing malaria transmission: a cross-sectional study in rural Western Kenya. *Malaria journal* **14**, 97, doi:10.1186/s12936-015-0613-7 (2015).

26 Peterson, D. S., Milhous, W. K. & Wellems, T. E. Molecular basis of differential resistance to cycloguanil and pyrimethamine in Plasmodium falciparum malaria. *Proceedings of the National Academy of Sciences of the United States of America* **87**, 3018-3022 (1990).

27 Foote, S. J., Galatis, D. & Cowman, A. F. Amino acids in the dihydrofolate reductase-thymidylate synthase gene of Plasmodium falciparum involved in cycloguanil resistance differ from those involved in pyrimethamine resistance. *Proceedings of the National Academy of Sciences of the United States of America* **87**, 3014-3017 (1990).

28 Sridaran, S. *et al.* Anti-folate drug resistance in Africa: meta-analysis of reported dihydrofolate reductase (dhfr) and dihydropteroate synthase (dhps) mutant genotype frequencies in African Plasmodium falciparum parasite populations. *Malaria journal* **9**, 247, doi:10.1186/1475-2875-9-247 (2010).

29 Bousema, J. T. *et al.* Moderate effect of artemisinin-based combination therapy on transmission of Plasmodium falciparum. *The Journal of infectious diseases* **193**, 1151-1159, doi:10.1086/503051 (2006).

30 Thwing, J. I. *et al.* In-vivo efficacy of amodiaquine-artesunate in children with uncomplicated Plasmodium falciparum malaria in western Kenya. *Tropical medicine & international health : TM & IH* **14**, 294-300, doi:10.1111/j.1365-3156.2009.02222.x (2009).

31 Clarke, S. E. *et al.* Effect of intermittent preventive treatment of malaria on health and education in schoolchildren: a cluster-randomised, double-blind, placebo-controlled trial. *Lancet* **372**, 127-138, doi:10.1016/S0140-6736(08)61034-X (2008).

32 Bassat, Q. *et al.* Dihydroartemisinin-piperaquine and artemether-lumefantrine for treating uncomplicated malaria in African children: a randomised, non-inferiority trial. *PloS one* **4**, e7871, doi:10.1371/journal.pone.0007871 (2009).

33 Zani, B., Gathu, M., Donegan, S., Olliaro, P. L. & Sinclair, D. Dihydroartemisinin-piperaquine for treating uncomplicated Plasmodium falciparum malaria. *Cochrane Database Syst Rev* **1**, CD010927, doi:10.1002/14651858.CD010927 (2014).

34 Zongo, I. *et al.* Randomized Noninferiority Trial of Dihydroartemisinin-Piperaquine Compared with Sulfadoxine-Pyrimethamine plus Amodiaquine for Seasonal Malaria Chemoprevention in Burkina Faso. *Antimicrobial agents and chemotherapy* **59**, 4387-4396, doi:10.1128/AAC.04923-14 (2015).

35 Bigira, V. *et al.* Protective efficacy and safety of three antimalarial regimens for the prevention of malaria in young ugandan children: a randomized controlled trial. *PLoS medicine* **11**, e1001689, doi:10.1371/journal.pmed.1001689 (2014).

36 Hatton, C. S. *et al.* Frequency of severe neutropenia associated with amodiaquine prophylaxis against malaria. *Lancet* **1**, 411-414 (1986).

37 Wilson, A. L. A systematic review and meta-analysis of the efficacy and safety of intermittent preventive treatment of malaria in children (IPTc). *PloS one* **6**, e16976, doi:10.1371/journal.pone.0016976 (2011).

38 Bojang, K. *et al.* A randomised trial to compare the safety, tolerability and efficacy of three drug combinations for intermittent preventive treatment in children. *PloS one* **5**, e11225, doi:10.1371/journal.pone.0011225 (2010).

39 Nakato, H., Vivancos, R. & Hunter, P. R. A systematic review and meta-analysis of the effectiveness and safety of atovaquone proguanil (Malarone) for chemoprophylaxis against malaria. *The Journal of antimicrobial chemotherapy* **60**, 929-936, doi:10.1093/jac/dkm337 (2007).

40 Faucher, J. F. *et al.* Efficacy of atovaquone/proguanil for malaria prophylaxis in children and its effect on the immunogenicity of live oral typhoid and cholera vaccines. *Clinical infectious diseases : an official publication of the Infectious Diseases Society of America* **35**, 1147-1154, doi:10.1086/342908 (2002).

41 Darpo, B. *et al.* Evaluation of the QT effect of a combination of piperaquine and a novel anti-malarial drug candidate OZ439, for the treatment of uncomplicated malaria. *British journal of clinical pharmacology*, doi:10.1111/bcp.12680 (2015).

42 Manning, J. *et al.* Randomized, double-blind, placebo-controlled clinical trial of a two-day regimen of dihydroartemisinin-piperaquine for malaria prevention halted for concern over prolonged corrected QT interval. *Antimicrobial agents and chemotherapy* **58**, 6056-6067, doi:10.1128/AAC.02667-14 (2014).

43 Lwin, K. M. *et al.* Randomized, double-blind, placebo-controlled trial of monthly versus bimonthly dihydroartemisinin-piperaquine chemoprevention in adults at high risk of malaria. *Antimicrobial agents and chemotherapy* **56**, 1571-1577, doi:10.1128/AAC.05877-11 (2012).

44 Nankabirwa, J. I. *et al.* Impact of intermittent preventive treatment with dihydroartemisinin-piperaquine on malaria in Ugandan schoolchildren: a randomized, placebo-controlled trial. *Clinical infectious diseases : an official publication of the Infectious Diseases Society of America* **58**, 1404-1412, doi:10.1093/cid/ciu150 (2014).

45 Cisse, B. *et al.* Randomized trial of piperaquine with sulfadoxine-pyrimethamine or dihydroartemisinin for malaria intermittent preventive treatment in children. *PloS one* **4**, e7164, doi:10.1371/journal.pone.0007164 (2009).

46 Mueller, B. U., Martin, K. J., Dreyer, W., Bezold, L. I. & Mahoney, D. H. Prolonged QT interval in pediatric sickle cell disease. *Pediatric blood & cancer* **47**, 831-833, doi:10.1002/pbc.20539 (2006).

47 Liem, R. I., Young, L. T. & Thompson, A. A. Prolonged QTc interval in children and young adults with sickle cell disease at steady state. *Pediatric blood & cancer* **52**, 842-846, doi:10.1002/pbc.21973 (2009).

48 Akgul, F. *et al.* Increased QT dispersion in sickle cell disease: effect of pulmonary hypertension. *Acta haematologica* **118**, 1-6, doi:10.1159/000100929 (2007).

49 *Proguanil - Summary of Product Characteristics*, <<https://www.medicines.org.uk/emc/medicine/2293>> (

50 *SP-AQ - Summary of Product Characteristics*, <<http://apps.who.int/prequal/WHOPAR/WHOPARPRODUCTS/MA098part4v1.pdf>> (

51 *DP- Summary of Product Characteristics*, <<http://www.ema.europa.eu/docs/en_GB/document_library/EPAR_-_Product_Information/human/001199/WC500118113.pdf>> (

52 Severe falciparum malaria. World Health Organization, Communicable Diseases Cluster. *Transactions of the Royal Society of Tropical Medicine and Hygiene* **94 Suppl 1**, S1-90 (2000).

53 Thornburg, C. D. *et al.* Impact of hydroxyurea on clinical events in the BABY HUG trial. *Blood* **120**, 4304-4310; quiz 4448, doi:10.1182/blood-2012-03-419879 (2012).

54 Rantala, A. M. *et al.* Comparison of real-time PCR and microscopy for malaria parasite detection in Malawian pregnant women. *Malaria journal* **9**, 269, doi:10.1186/1475-2875-9-269 (2010).

55 Ivanova, A., Qaqish, B. F. & Schell, M. J. Continuous toxicity monitoring in phase II trials in oncology. *Biometrics* **61**, 540-545, doi:10.1111/j.1541-0420.2005.00311.x (2005).

56 Hasselblad, V. & Allen, A. S. Power calculations for large multi-arm placebo-controlled studies of dichotomous outcomes. *Statistics in medicine* **22**, 1943-1954, doi:10.1002/sim.1427 (2003).

57 Efficacy and safety of the RTS,S/AS01 malaria vaccine during 18 months after vaccination: a phase 3 randomized, controlled trial in children and young infants at 11 African sites. *PLoS medicine* **11**, e1001685, doi:10.1371/journal.pmed.1001685 (2014).

58 Corportation, C. C. *Compaq Fortran Language Reference Manual*. (Digital Equipment Corporation, 1999).

59 Wang, W. C. *et al.* Hydroxycarbamide in very young children with sickle-cell anaemia: a multicentre, randomised, controlled trial (BABY HUG). *Lancet* **377**, 1663-1672, doi:10.1016/S0140-6736(11)60355-3 (2011).

60 Bartko, J. J. The negative binomial distribution: a review of properties and applications. *Virginia journal of science* **12**, 18-37 (1961).

## APPENDIX A: STUDY INFORMATION SHEET (ENGLISH)

## APPENDIX B: STUDY INFORMATION SHEET (KISWAHILI)

## APPENDIX C: STUDY INFORMATION SHEET (KISWAHILI TO ENGLISH)

## APPENDIX D: STUDY INFORMATION SHEET (DHOLUO)

## APPENDIX E: STUDY INFORMATION SHEET (DHOLUO TO ENGLISH)

## APPENDIX F: INFORMED CONSENT (ENGLISH)

Enrollment Consent to Participate in a Research Study

Title of Study: Enhancing Preventive Therapy of Malaria in Children with Sickle Cell Anemia in East Africa (EPiTOMISE).

**Investigator:** Dr. Festus Njuguna

You are being asked to allow your child to take part in this research study because your child has sickle cell anemia (SCA).

WHAT ARE SOME GENERAL THINGS TO KNOW ABOUT RESEARCH STUDIES?

You are being asked to allow your child to participate in a clinical research study. Before agreeing to allow your child to participate in this clinical research study, it is important that you understand that studies like these only include people who choose to take part. Your child’s participation is voluntary. Please read this consent form carefully and take your time making your decision. Your child’s study doctor or study staff will talk about this consent form with you. Please ask about any words or information that you do not clearly understand.

This consent form describes what will happen in this study. This form describes the risks, inconveniences, discomforts, and other important information. It is important that you ask questions and understand what is required for participation in this research study. We encourage you to talk with your family and friends before you decide to allow your child to take part in this research study. You may take home a copy of the form to review, while you decide if you would like your child to participate in the study. If you agree to allow your child to participate and sign this form, you will be given a signed and dated copy of this form to keep.

Please tell the study doctor or study staff if your child is taking part in another research study.

WHO WILL BE MY STUDY DOCTOR?

If you decide to participate, Dr. Njuguna will be your doctor for the study and will be in contact with your regular health care provider throughout the time that you are in the study and afterwards, if needed.

A grant from the United States National Institutes of Health (NIH) will sponsor this study. Portions of Dr. Njuguna and his research team’s salaries will be paid by this grant.

WHY IS THIS STUDY BEING DONE?

You are being asked to allow your child to take part in this study because he/she is between the ages of 1 and 10 years of age, has SCA, and lives in an area with a lot of malaria.

We want to find better ways to prevent malaria in children with SCA. This study will help us identify the best medications that can be used to prevent malaria in children with SCA in parts of Africa that have malaria.

In this study, we are going to compare three malaria drugs that are already available in your local pharmacy in order to see which of the medications, if any, is better at reducing the number of times your child has malaria.

The study medications included in this research study are listed below. In this study, your child will take one of the following three medications by mouth as malaria prevention:

- Proguanil (also known as Paludrine) is the current standard of care for prevention of malaria in children with SCA in Kenya. Your child may already be taking this medication to prevent malaria. This medication is taken daily.

OR

- Sulfadoxine/Pyrimethamine-Amodiaquine (SP-AQ) is a combination of medications comprised of sulfadoxine and pyrimethamine taken at the same time as amodiaquine. This medication is taken monthly.

OR

- Dihydroartemisinin-Piperaquine (DP) is a combination medicine of dihydroartemisinin and piperaquine. This medication is taken monthly.

In addition, if your child does get malaria, he/she will be treated by mouth with a medication called Artemether-Lumefantrine (AL). All children under 5 years of age will also receive daily penicillin by mouth, which is intended to prevent bacterial infections and is standard of care for children with SCA.

HOW MANY CHILDREN WILL TAKE PART IN THIS STUDY?

About 246 children will complete this research study. This study will be done at Homa Bay County Hospital in Homa Bay, Kenya.

WHAT IS INVOLVED IN THE STUDY?

If you agree to allow your child to be in this study, you will be asked to sign this consent form. Your child will have the following procedures to make sure that your child is eligible for the study:

Screening Visit

Before participating in the study, the study personnel need to determine if your child is eligible to participate. This helps to improve the safety of participating in the study.

During the screening visit, the following procedures will be conducted and information recorded:

- We will review your child’s medical history
- We will review medications that your child is taking
- We will perform an electrocardiogram (ECG), in which the electrical activity of your child’s heart is measured through his/her skin
- We will collect approximately 3 ½ milliliters (mL) of blood to test your child’s red blood cells for the proteins that cause SCA and to perform routine blood tests to determine if it is safe to enroll in the study.

Enrollment Visit

During the enrollment visit, the following procedures will be conducted and information recorded:

- We will review and record your child’s medical history, including any medical events that have occurred since the previous visit
- We will record medicines your child is taking
- We will conduct a physical examination, including height and weight
- We will collect a fingerprick blood sample of less than ½ mL
- If necessary, we will administer to your child a Meningococcal vaccine.  This vaccine will help protect your child against bacteria that causes meningitis. This may not be necessary if your child has already received this vaccine, which is routinely recommended in Kenya.
- We will provide you with a 30-day supply of daily penicillin (if your child is less than 5 years old), which is a standard medication used to prevent bacterial infections in children with SCA
- We will enroll your child into the Kenyan National Hospital Insurance Fund (NHIF);
- We will provide you with your child’s malaria prevention study medication and instructions on how to take it.

Also at this enrollment visit we will determine which malaria prevention study medication your child will receive. To do so, your child will be randomly assigned (like drawing numbers from a hat) to receive either daily Proguanil, monthly SP-AQ, or monthly DP. Your child has a 1 in 3 chance of receiving a particular study drug. Whichever drug that your child is assigned to receive through this process will be the malaria prevention drug that they receive throughout the study. All children will receive some type of malaria prevention medication.

Routine Follow up Visits (monthly)

At each monthly visit, the following procedures will be conducted:

- We will review your child’s medical history, including medical events since the last visit
- We will perform a physical exam, including height and weight
- We will collect a fingerprick blood sample of less than ½ mL that will be used to test your child’s blood for anemia levels and for malaria parasites.
- If your child has a fever or has had a fever within the last 24 hours, he/she will have an immediate test for malaria parasites; if your child has not had fever, then the blood will be saved for testing at the end of the study.
- Every third monthly visit, we will collect approximately 2½ mL of blood for routine testing
- At the 6-month visit and final 12-month visit, we will collect an additional 1 mL of blood to test for sickle-cell red blood cells
- If you sign the separate consent form agreeing to an additional blood collection, at either the 3, 6, 9, or 12 month visit we will collect an additional 2½ mL of blood for future research studies.
- If necessary, we will perform an ECG;*
- We will provide you with the study medication and instructions on how to take it;
- We will provide you with a 30 day supply of daily penicillin (if less than 5 years old).

*Approximately 20 children will be asked to return to clinic 2 days later to have repeat ECG done each month during the study; this will be to track the results of the ECG on an ongoing basis throughout the entire period. This will only be some of the children assigned to the DP arm of the study.

If your child has a positive blood test for malaria, he/she will be treated at that time with AL.

Acute-Care Visits (as needed)

For the study, if your child at home has a fever, either because they feel hot or because their temperature is measured above 37.5 degrees Celsius, we are asking that you call the study personnel in order to arrange to be evaluated by one of the study clinicians. At this visit, the following procedures will be conducted:

- We will review your child’s medical history, including medical events since the last visit
- We will perform a physical exam, including height and weight
- We will collect a fingerprick blood sample of less than ½ mL.
- If your child has a fever or has had a fever within the last 24 hours, he/she will have an immediate test for malaria parasites; if your child has not had fever, then the blood will be saved for testing at the end of the study.

If your child has a positive blood test for malaria, he/she will be treated at that time with AL.

HOW LONG WILL MY CHILD BE IN THIS STUDY?

The expected duration of study participation is monthly for 12 months. Your child’s study participation will end after the month 12 final visit.

You can choose to stop participating at any time without penalty. In that case, you will no longer receive medical care or benefits that are provided by the study, and your child‘s care would be transferred to clinicians who are not involved in the study, such as your pediatrician, hospital or clinic. However, if you decide to stop participating in the study, we encourage you to talk to your doctor first.

WHAT ARE THE RISKS OF THE STUDY?

As a result of your participation in this study, your child is at risk for the following side effects. If you choose to participate, you should discuss these with the study doctor and your regular health care provider.

It is possible for any drug to cause unwanted side effects. You need to know about side effects that could occur in this study before you decide whether your child should participate. There may be risks, discomforts, drug interactions or side effects that are not yet known. These risks include possible worsening of the complications of sickle cell anemia. If these complications are serious enough, they can be fatal.

Proguanil may cause some, all or none of the side effects listed below.

More likely:

- Nausea Anemia
- Vomiting Allergic reactions
- Abdominal pain Depression
- Abnormal dreams Cough
- Headache Insomnia
- Diarrhea Rash
- Weakness Fever
- Loss of appetite Dizziness
- Itching

Less Likely:

- Nervousness
- Rapid, strong, or irregular heartbeat
- Mouth ulcers
- Hair loss
- Severe rash

SP-AQ may cause some, all or none of the side effects listed below.

More likely:

- Headache Cough
- Vomiting Nausea
- Loss of appetite Abdominal Pain
- Diarrhea Weakness
- Fever Tiredness
- Difficulty sleeping Rash/skin eruptions
- Sleepiness Itching

Less Likely

- Eye Problems/Retinopathy
- Liver Problems/Hepatitis
- Anemia

DP may cause some, all or none of the side effects listed below.

More likely:

- Headache
- Fever
- Cough
- Influenza
- Diarrhea

Less Likely:

- Dizziness
- Vomiting
- Nausea
- Loss of appetite

OTHER RISKS

As a result of drawing blood from your child’s arm or from a finger prick, your child may experience momentary discomfort and/or bruising. Infection, excess bleeding, clotting, or fainting are also possible, although unlikely. To reduce the chances of these risks, we will hire only trained study staff and appropriate supplies for blood draws.

In addition, your child may experience unforeseeable risks and inconveniences associated with the use of the study drug. These may include possible worsening of the complications of sickle cell anemia, which, when serious, can be fatal. You should report any problems you have to the study team.

ARE THERE BENEFITS TO TAKING PART IN THE STUDY?

If you agree to allow your child to take part in this study, there may be direct medical benefit to your child. Child participants will receive free study medications to prevent malaria and bacterial infections, monthly comprehensive assessments, free testing for malaria parasites when they have a fever and enrollment in the Kenyan National Hospital Insurance Fund. These services have the potential to reduce medical problems from both malaria and SCA.

In the long-term, we hope that in the future the information learned from this study will benefit children in Africa with SCA, by finding better medications to prevent malaria.

WHAT ALTERNATIVES ARE THERE TO PARTICIPATION IN THIS STUDY?

Your child does not have to participate in this study. Even if you do not allow your child to participate, he/she can still receive regular medical treatment from their regular medical providers.

WILL MY CHILD’S INFORMATION BE KEPT CONFIDENTIAL?

Your child will not be identified by name, or any other facts that may point to him or her in study records shared outside of Homa Bay County Hospital. For records disclosed outside of Homa Bay County Hospital, your child will be assigned a unique code number. The log or list which includes the code number and your child’s name and/or other personal identifier is confidential and will be kept secure by the research team.

Every effort will be made to maintain the confidentiality of your child’s medical records. However, this cannot be guaranteed. There is the potential risk of loss of confidentiality. Certain offices and people other than the researchers may look at your child’s medical charts and study records. These include people from:

- The study teams at Moi University, Duke University, and the Duke Clinical Research Institute;
- The Moi University Institutional Research & Ethics Committee and the Duke University Health System Institutional Review Board;
- Representatives from the United States NIH.

Study records will be retained for ten years after the end of the study per local regulations. Study information in your child’s medical records will be kept forever.

During the course of this study, your doctor and the research team may share study information with certain individuals. These may include:

- Study monitors, who verify the accuracy of the information;
- Individuals with medical backgrounds who determine the effect that the drugs have on your child’s disease; and/or
- Individuals who put all the study information together in report form.

While your child is in this research study, research information collected about him/her might be put in his/her medical record.

You have the right to stop participating in this study at any time. To stop participating, you should let your study doctor know that you want to stop it. If you want your child to participate in this research study, you must sign this consent form to give the study team permission to look at your child’s health information. If you refuse to allow us to review your child’s health information, your child will not be able to be in the research study. However, not signing this form will not affect you or your child’s access to medical care.

While the information and data resulting from this study may be presented at scientific meetings or published in a scientific journal, your child’s identity will not be revealed and your child will not be mentioned by name. A description of this clinical trial will be available on <http://www.ClinicalTrials.gov> and the Pan African Clinical Trials Registry (http://www.pactr.org). These web sites will not include information that can identify you. At most, the web sites will include a summary of the results. You can search these web sites at any time.

Your child’s records will not be used for any other purpose or shared with anyone else without your permission.

WHAT ARE THE COSTS?

There will be no additional costs to you or your child as a result of being in this study. Any study procedures, study drugs, or study tests will be provided to your child free of charge. In addition, if your child participates in the study, we will enroll your child in the Kenya NHIF while they participate in the study. Some types of medical care may be paid by the NHIF.

WHAT ABOUT COMPENSATION?

There will be no money or compensation for your child’s participation in this study. However, you will receive KSh/500 for help with transportation to your scheduled follow up visits. This may not cover the full costs of transportation, but will help make it easier to fully participate in the study.

WHAT IF MY CHILD IS INJURED?

Immediate necessary medical care is available at Homa Bay County Hospital in the event that your child is injured as a result of participation in this research study. There is no commitment by Moi University, Duke University through its Duke Clinical Research Institute, Homa Bay County Hospital, or the NIH to provide any reimbursement or payment for any study-related injury costs, money or free medical care.

For questions about the study or research-related injury, contact Dr. Njuguna at +XXXXXXXXX during regular business hours and at +XXXXXXXXX after hours and on weekends and holidays.

WHAT ABOUT MY RIGHTS TO DECLINE MY CHILD’S PARTICIPATION OR WITHDRAW FROM THE STUDY?

You may choose to not allow your child to be in the study. You may also choose to allow your child to be in the study and you may withdraw him/her from the study at any time. If you withdraw your child from the study, no new data about your child will be collected for study purposes unless your child has a side effect related to the study drug. If a side effect like this occurs, we may need to review your child’s entire medical record. All information collected for the study will be sent to the study sponsor, which is the United States’ NIH.

Your decision not to allow your child to participate or to withdraw from the study will not involve any penalty, although you will no longer receive medical care or benefits that are provided by the study and your child‘s care would be transferred to clinicians who are not involved in the study, such as your pediatrician, hospital or clinic. This will not affect you or your child’s access to health care at through their regular medical providers.

INVOLUNTARY REMOVAL FROM THE STUDY

The study doctors may decide to take your child out of this study if:

- your child’s condition gets worse, or
- your child has a serious side effect, or
- needs treatment not allowed in the study, or
- the study doctors decide it is no longer in your child’s best interest to continue.

The NIH, Dr. Njuguna or other study doctors, or regulatory agencies may stop this study at any time without your consent. If this occurs, you will be told and your study doctor will talk to you about other options.

WHAT IF THERE IS NEW INFORMATION?

You will be given any new information learned during the course of the study that might affect you whether you agree to continue your child’s participation in the study.

WHOM DO I CALL IF I HAVE QUESTIONS OR PROBLEMS?

For questions about the study or a research-related injury or if you have problems, concerns, or suggestions about the research, contact Dr. Njuguna at +XXXXXXXXX during regular business hours, after hours, and on weekends and holidays. For questions about your child’s rights as a research participant, or to talk about problems, concerns, or suggestions related to the research, or to obtain information or offer input about the research, contact the Moi’s Institutional Research Ethics Committee (IREC) at +2540787723677. The Duke University Health System Institutional Review Board has also reviewed this study, if you have any questions that those members can assist with please direct them to +1(919) 668-5111.

STATEMENT OF CONSENT

The purpose of this study, procedures to be followed, risks, and benefits have been explained to my child and me. I have been allowed to ask questions, and my questions have been answered to my satisfaction. I have been told whom to contact if I have questions, to talk about problems, concerns, or suggestions related to this research study. I have read this consent form and agree for my child to be in this study, with the understanding that I may withdraw my child at any time. I have been told that I will be given a signed and dated copy of this consent form.

___________________________________

Printed Name of Parent or Legal Guardian

____________________________________

Signature of Parent or Legal Guardian Date Time: ___: ____

____________________________________ ______________________________

Signature of Witness (if applicable) Date Time

I have fully explained the research study described by this form. I have answered the parent/guardian’s questions and will answer any future questions to the best of my ability. I will tell the family of any changes in the procedures or in the possible harms/possible benefits of the study that may affect parent/guardian’s willingness to have their child stay in the study.

____________________________________ ___________

Printed Name of Researcher Obtaining Date

Parental Permission or Consent

____________________________________ ___________

Signature of Researcher Obtaining Date

Parental Permission or Consent

WHAT WILL BLOOD SAMPLES BE USED FOR?

During the course of this study, over 12 months your child will have blood collected in an amount specified above (this enrollment visit and then 12 monthly visits). We will collect this blood to perform routine testing to monitor the effects of SCA, malaria, and the study medications.

In addition, one blood sample will collected and used only for future research, if you agree to this request. These samples will be stored for a long time at one of the universities or research organizations conducting this study, including Moi University or Duke University. Samples may also be shared with investigators at other institutions. Any use of these stored samples must be approved by an institutional review or ethics board.

Here are a few important considerations for these samples:

- We will collect and store approximately 2.5 mL of blood for future research during a visit at which we are collecting blood for routine care. This sample will only be used to study malaria, SCA, or other infectious diseases in children with SCA.
- We will also store a blood spot on special paper from the blood we have collected from you. After the study is over, this will be used to test for malaria parasite genes.
- The information that we get from these studies will not affect your child’s care.
- These samples will not be sold or used for the production of commercial products.
- We may perform research on the human or malaria genes in these samples, but no information will be placed in your child’s medical record, and samples will be coded so that nobody can figure out the identity of your child.
- There is no direct benefit for your child if you allow us to use samples for these uses. However, if they can be used for future research studies, we hope that these will help us learn how to better prevent, treat, or cure medical problems in children with SCA.

Every effort will be made to protect your confidential information, but this cannot be guaranteed. The information that may be obtained as a result of your participation in future research will not be included in your medical record.

IF I AGREE TO PROVIDE SAMPLES NOW, CAN I REQUEST THAT MY SAMPLES BE DESTROYED AT A LATER DATE?

If you decide later that you would like your samples destroyed, you must inform Dr. Njuguna or his representatives on the study team that you are withdrawing your permission for your samples to be stored and used for future research.

- During the main study, please contact the study coordinator Mr. Joseph Kipkoech at +XXXXXXXXX.
- After the main study, please contact the Moi’s Institutional Research Ethics Committee (IREC) at +2540787723677.

If the information linking your coded samples to you has already been destroyed, your samples can no longer be linked to you and we will be unable to locate them for destruction.

Also, even though your samples will be destroyed:

- We cannot get back samples or information that we have already given out to researchers
- You cannot withdraw your sample if it has already been analyzed or tested

Please sign on one signature line below whether you agree to have your blood samples stored for possible future research, or if you do not agree to have your blood samples stored.

I agree to allow my child’s blood samples to be stored for possible future research of SCA, malaria, and other infections in children with SCA.

Parent or Legal Guardian Signature Date

I DO NOT agree to allow my child’s blood samples to be stored for possible future research of SCA, malaria, and other infections in children with SCA.

Parent or Legal Guardian Signature Date

______________________________________________________________________Witness Signature (if applicable) Date

## APPENDIX G: INFORMED CONSENT (KISWAHILI)

Enrollment Consent to Participate in a Research Study

Title of Study: Enhancing Preventive Therapy of Malaria in Children with Sickle Cell Anemia in East Africa (EPiTOMISE).

**Mtafiti:**DaktariFestus Njuguna

Unaulizwa kuruhusu mtoto wako ashiriki katika utafiti huu kwa sababu mtoto wako ako na Sickle Cell Anemia (SCA).

NI MAMBO GANI YA JUMLA UNAYOSTAHILI KUJUA KUHUSU TAFITI ZA MASOMO?

Unaulizawa kuruhusu mtoto wako ashiriki katika utafiti wa kimasomo ya kliniki.Kabla ya kukubali kumruhusu mtoto wako ashiriki katika huu utafiti wa kimasomo ya kliniki, ni muhimu uelewe kwamba tafiti kama hizi hujumuisha tu watu ambao huchagua kushiriki. Kushiriki kwa mtoto wako ni kwa hiari. Tafadhali soma fomu hii ya idhini kwa makini na uchukue muda wako kufanya uamuzi wako.Daktari wa utafiti wa mtoto wako au wafanyakazi wa utafiti watazungumza kuhusu hii fomu ya idhini na wewe. Tafadhali uliza kuhusu mambo yoyote au habari ambayo hauelewi kwa kina

Hii fomu ya idhini inaeleza ni nini kitatendeka katika utafiti huu.Hii fomu inaelezea hatari, shida, ugumu na mambo mengine muhimu. Ni muhimu kwamba uulize maswali na uelewe kile kinachohitajika kwa ajili ya kushiriki katika utafiti huu.Tunakuhimiza uzungumze na familia yako na marafiki kabla ya kuamua kumruhusu mtoto wako ashiriki katika utafiti huu. Unaweza kuenda nyumbani na nakala ya fomu hii uipitie huku ukiamua kama ungependa mtoto wako ashiriki katika utafiti. Ikiwa utakubali kumruhusu mtoto wako ashiriki na utie sahihi fomu hii, utapewa nakala ya fomu hii iliyotiwa sahihi na tarehe uweke.

Tafadhali ambia daktari wa utafiti au wafanyakazi wa utafiti ikiwa mtoto wako anashiriki katika utafiti mwingine.

NANI ATAKUWA DAKTARI WANGU WA UTAFITI?

Ikiwa utakubali kushiriki, Daktari Njuguna atakuwa daktari wako wa utafiti na atawasiliana na mhudumu wako wa afya wa kawaida katika wakati wote ambao wewe utakuwa kwenye utafiti na baada ya hapo, ikiwa itahitajika.

Ruzuku kutoka kwa kwaTaasisi ya Taifa ya Afya ya Marekani (NIH) itadhamini utafiti huu. Sehemu ya mishahara ya Daktari Njuguna na timu yake ya utafiti italipwa na ruzuku hii.

KWA NINI UTAFITI HUU UNAFANYWA?

Unaulizwa kuruhusu mtoto wako ashiriki katika utafiti huu kwa sababu ako kati ya umri wa mwaka 1 na umri wa miaka 10, ako na SCA, na anaishi katiki eneo lililo na malaria mengi.

Tunataka kutafuta njia bora za kuzuia malaria kwa watoto walio na SCA. Huu utafiti utatusaidia kutambua dawa bora ambazo zinaweza kutumika kuzuia malaria kwa watoto walio na SCA katika maeneo ya Afrika ambayo yana malaria.

Katika utafiti huu, tutalinganisha madawa matatu ya malaria ambayo tayari yanapatikana katika maduka ya madawa ya eneo lako ili kuona ni madawa gani, kama yapo, ni bora katika kupunguza idadi ya nyakati mtoto wako anakuwa na malaria.

Dawa za utafiti zilizojumuishwa katika utafiti huu zimeorodheshwa hapo chini. Katika utafiti huu, mtoto wako atachukua moja ya dawa za aina tatu zifuatazo kwa kinywa ili kuzuia malaria:

- *Proguanil* (pia inajulikana kama *Paludrine*) ni kipimo cha sasa cha utunzaji kwa ajili ya kuzuia malaria kwa watoto walio na SCA nchini Kenya. Mtoto wako anaweza kuwa tayari anatumia dawa hii kuzuia malaria. Dawa hii humezwa kila siku.

AU

- *Sulfadoxine/Pyrimethamine-Amodiaquine (SP-AQ*) ni mchanganyiko wa madawa zikiwemo za *sulfadoxine* na *pyrimethamine* zinazomezwa wakati mmoja kama amodiaquine. Dawa hizi humezwa kila mwezi.

AU

- Dihydroartemisinin-Piperaquine (DP) ni dawa mchanganyiko wa dihydroartemisinin na piperaquine.Dawa hii humezwa kila mwezi.

Aidha, ikiwa mtoto wako atapata malaria, yeye atatibiwa kwa kinywa na dawa iitwayo Artemether-Lumefantrine (AL). Watoto wote chini ya umri wa miaka 5 pia watapokea penicillin kila siku kwa kinywa, ambayo inalengwa kuzuia maambukizi ya bacteria na ni kipimo cha matunzo kwa watoto walio na SCA.

NI WATOTO WANGAPI WATASHIRIKI KATIKA UTAFITI HUU?

Karibu watoto 246 watakamilisha utafiti huu. Utafiti huu utafanywa katika hospitali ya kaunti ya Homa Bay, katika Homa Bay, Kenya.

NINI KINACHOHUSISHWA KATIKA UTAFITI HUU?

Ikiwa utakubali kuruhusu mtoto wako ashiriki katika huu utafiti, utaulizwa kutia sahihi hii fomu ya idhini. Mtoto wako atafanyiwa taratibu zifuatazo ili kuhakikisha kwamba anastahili kuwa kwa utafiti:

Ziara ya Uchunguzi

Kabla ya kushiriki katika utafiti, wafanyakazi wa utafiti watahitaji kutambua ikiwa mtoto wako ana haki ya kushiriki. Hii husaidia kuboresha usalama wa kushiriki katika utafiti.

Wakati wa ziara ya uchunguzi, taratibu zifuatazo zitafanywa na kurekodiwa:

- Tutapitia historia ya matibabu ya mtoto wako
- Tutapitia madawa ambayo mtoto wako anatumia
- Tutafanya uchunguzi wa *electrocardiogram (ECG),*ambapo shughuli za umeme katika moyo wa mtoto wako hupimwa kwa kupitia ngozi yake
- Tutakusanya takriban mililita 31/2 (mL) za damu ili kupima seli nyekundu za damu za mtoto wako kuona protini zinazosababisha SCA na kufanya uchunguzi wa damu ili kubaini ikiwa ni salama kumhusisha katika utafiti.

Ziara ya Kujiunga

Wakati wa ziara ya kujiunga, taratibu zifuatazo zitafanywa na taarifa kurekodiwa:

- Tutapitia na kurekodi historia ya matibabu ya mtoto wako, ikiwa ni pamoja na matukio yoyote ya matibabu yaiyotokea tangu ziara iliyopita.
- Tutarekodi madawa ambayo mtoto wako anatumia
- Tutafanya uchunguzi wa mwili, ikiwa ni pamoja na urefu na uzito.
- Tutakusanya sampuli ya damu kutoka kwa kidole chini ya mililita ½
- Iwapo ni muhimu, tutampa mtoto wako chanjo ya *Meningococcal*.  Chanjo hii itasaidia kumkinga mtoto wako dhidi ya bakteria zinazosababisha meningitis. Hii haitakuwa muhimu ikiwa mtoto wako tayari ashapokea chanjo hii, ambyo kwa kawaida imependekezwa nchini Kenya.
- Tutakupatia ugavi wa penicillin wa siku 30 (ikiwa mtoto wako ako chini ya umri wa miaka 5), ambayo ni dawa inayotumika kuzuia maambukizi ya bakteria katika watoto walio na SCA.
- Tutaandikisha mtoto wako katika bima ya kitaifa ya hospitali ya Kenya (NHIF)
- Tutakupatia madawa ya utafiti ya kuzuia uambukizaji wa malaria katika mtoto wako na maelekezo ya jinsi atakavyoyatumia.

Pia katika ziara hii ya kujiunga tutaamua ni dawa gani za utafiti za kuzuia malaria ambazo mtoto wako atapokea.Kufanya hivyo, mtoto wako, bila mpangilio (kama kutoa nambari kutoka kwa kofia) atawekwa kupokea aidha *Proguanil* kila siku, *SP-AQ* kila mwezi, au *DP* kila mwezi. Mtoto wako ana fursa 1 kati ya 3 ya kupata dawa Fulani ya utafiti.

Namna yeyote ya dawa ambayo mtoto wako atawekwa kupokea kupitia utaratibu huu, itakuwa dawa ya kuzuia malaria atakayoipokea katika utafiti wote. Watoto wote watapokea aina fulani ya dawa ya kuzuia malaria.

Ziara za kufuatilia zilizopangwa (kila mwezi)

Katika kila ziara ya kila mwezi, taratibu zifutazo zitafanywa:

- Tutapitia historia ya matibabu ya mtoto wako, ikiwa ni pamoja na matukio ya matibabu tangu ziara iliyopita
- Tutafanya uchunguzi wa mwili, ikiwa ni pamoja na urefu na uzito.
- Tutakusanya sampuli ya damu iliyo chini ya mililita ½ kutoka kwa kidole kilichodungwa ambayo itatumika kupima kiwango cha anemia kwa mtoto wako na vimelea vya malaria .
- Ikiwa mtoto wako ana joto au amekuwa na joto ndani ya masaa 24 iliyopita, yeye atachunguzwa vijidudu vya malaria mara moja; ikiwa mtoto wako hajakuwa na joto, basi damu itahifadhiwa kwa ajili ya uchunguzi mwishoni mwa utafiti.
- Kila ziara ya kila mwezi wa tatu, tutakusanya takriban mililita 2 ½ ya damu kwa upimaji wa mara kwa mara.
- Katika ziara ya mwezi wa 6 na ziara ya mwisho ya mwezi wa 12, tutakusanya ziada ya mililita 1 ya damu ili kuchunguza seli nyekundu za damu zilizo na selimundu.
- Ikiwa utatia saini fomu tofauti itakayoidhinisha kukusanya damu zaidi katika ziara ya mwezi wa 3, 6, 9 ama 12, tutachukua mililita 2 ½ zaidi ya damu kwa ajili ya utafiti wa baadaye.
- Ikiwa ni itahitajika, tutafanya *ECG*;*
- Tutakupatia dawa za utafiti na maelekezo ya jinsi ya kuzitumia;
- Tutakupatia ugavi wa siku 30 wa penicillin ya kila siku ( ikiwa ako chini ya umri wa miaka 5).

*Takriban watoto 20 wataulizwa kurudi kwa kliniki siku 2 baadaye ili warudie kufanya *ECG* kila mwezi wakati wa utafiti; hii itakuwa kufuatilia matokeo ya *ECG* juu ya misingi endelevu katika kipindi chote.Hii itakuwa tu kwa baadhi ya watoto waliowekwa katika kikundi cha DP cha utafiti

Ikiwa mtoto wako atapimwa damu na awe na malaria, yeye atatibiwa wakati huo na AL.

Ziara ya Huduma ya papo hapo (kama inahitajika)

Katika huu utafiti, ikiwa mtoto wako yuko nyumbani na ana joto, aidha kwa sababu anahisi joto au kipimo cha joto la mwili kiko juu ya nyuzi 37.5, tunakuomba kwamba upigie simu wafanyakazi wa utafiti ili wapange aonwe na mmoja wa madaktari.Katika ziara hii, taratibu zifuatazo zitafanyika:

- Tutapitia historia ya matibabu ya mtoto wako, ikiwa ni pamoja na matukio ya matibabu tangu ziara iliyopita.
- Tutafanya uchunguzi wa kimwili, ikiwa ni pamoja na urefu na uzito.
- Tutakusanya sampuli ya damu iliyo chini ya mililita ½. Ikiwa mtoto wako ana joto au amekuwa na joto ndani ya masaa 24 iliyopita, yeye atachunguzwa vijidudu vya malaria mara moja; ikiwa mtoto wako hajakuwa na joto, basi damu itahifadhiwa kwa ajili ya uchunguzi mwishoni mwa utafiti.

Ikiwa mtoto wako atapimwa damu na awe na malaria, yeye atatibiwa wakati huo na AL.

MTOTO WANGU ATAKUWA KWA UTAFITI KWA MUDA GANI?

Inatarajiwa muda wa kushiriki kwa utafiti ni wa kila mwezi kwa miezi 12. Kushiriki kwa mtoto wako katika utafiti kutaisha baada ya ziara ya mwisho ya mwezi wa 12.

Unaweza kuchagua kuwacha kushiriki wakati wowote bila adhabu. Katika hali hiyo, hutaweza tena kupokea huduma ya afya au faida zinazotolewa na utafiti, na kuhudumiwa kwa mtoto wako kutahamishiwa madaktari wengine ambao hawahusiki katika utafiti, kama vile daktari wa mtoto wako, hospitali au kliniki. Hata hivyo, ikiwa utaamua kuacha kushiriki katika utafiti, tunakuhimiza uzungumze na daktari wako kwanza.

HATARI ZA UTAFITI NI GANI?

Kutokana na kushiriki kwako katika utafiti huu, mtoto wako ako katika hatari ya athari zifuatazo. Ikiwa utachagua kushiriki, unapaswa kujadili haya na daktari wa utafiti na mhudumu wako wa afya wa kawaida.

Inawezekana dawa yoyote kusababisha athari zisizotakikana. Unahitajika kujua kuhusu athari zinazoweza kutokea katika huu utafiti kabla hujaamua ikiwa mtoto wako anapaswa kushiriki. Kunaweza kuwa na hatari, usumbufu, mwingiliano wa madawa au athari ambazo bado hazijulikani. Athari hizi zaweza kuwa ni pamoja na kuongezeka kwa matatizo makubwa ya selimundu na anemia.Ikiwa matatizo haya ni makubwa, yanaweza yakasabisha kifo.

*Proguanil* inaweza kusababisha baadhi, yote au isisababishe madhara yaliyoorodheshwa hapa chini.

Uwezekano mkubwa:

- Kichefuchefu Upungufu wa damu
- Kutapika Athari mzio
- Maumivu ya tumbo majonzi
- Ndoto zisizo za kawaida Kukohoa
- Kuumwa na kichwa Kukosa usingizi
- Kuhara Upele
- Udhaifu Joto
- Kupoteza hamu ya chakula Kizunguzungu
- Kujikuna

Uwezekano mdogo

- Kuwa mwoga
- Moyo kudunda kwa haraka, kwa nguvu au kusiko kwa kawaida
- Vidonda mdomoni
- Nywele kupotea
- Upele mkali

*SP-AQ* inaweza kusababisha baadhi, yote au isisababishe madhara yaliyoorodheshwa hapa chini.

Uwezekano mkubwa:

- Kuumwa na kichwa Kukohoa
- Kutapika Kichefuchefu
- Kupoteza hamu ya chakula Maumivu ya tumbo
- Kuhara Udhaifu
- Joto Uchovu
- Ugumu katika kulala Upele/milipuko ya ngozi
- Kuhisi usingizi Kujikuna

Uwezekano mdogo

- Matatizo ya macho/kuharibika kwa retina
- Matatizo ya ini/hepatitis
- **Anemia**

*DP* inawza kusababisha baadhi, yote au isisababishe madhara yaliyotajwa hapa chini..

Uwezekano mkubwa:

- Kuumwa na kichwa
- Joto
- Kukohoa
- Homa ya mafua
- Kuhara

Uwezekano mdogo:

- Kizunguzungu
- Kutapika
- Kichefuchefu
- Kupoteza hamu ya chakula

HATARI ZINGINE

Kutokana na kutoa damu kutoka kwa mkono wa mtoto wako au kudunga kidole chake, mtoto wako anaweza kuwa na maumivu na/au kuchibuka. Maambukizi, kutoka damu kupita kiasi, kugandamana, au kuzirai pia kunawezekana, ijapokuwa kwa ugumu.Ili kupunguza uwezekano wa hizi hatari, tutaajiri tu wafanyakazi wa utafiti waliohitimu na vifaa sahihi vya kutoa damu.

Kwa kuongezea, mtoto wako anawezakumbwa na hatari zisizoweza kutabirika na kero linalohusishwa na matumizi ya dawa za utafiti. Hizi zinaweza kujumuisha kuongezeka kwa uwezekano wa matatizo ya selimundu na anemia ambayo ikiwa mbaya sana, inaweza kusababisha maafa. Unapaswa kuripoti matatizo yoyote anayokuwa nayo kwa timu ya utafiti.

KUNA MANUFAA YA KUSHIRIKI KATIKA UTAFITI?

Ikiwa utaruhusu mtoto wako ashiriki katika utafiti huu, kunaweza kuwa na manufaa ya matibabu ya moja kwa moja kwa mtoto wako. Watoto washiriki watapata madawa ya bure ya utafiti ya kuzuia malaria na maambukizi ya bakteria, tathmini ya kina kila mwezi, kuchunguzwa bure kwa vijidudu vya malaria wanapokuwa na joto na usajili katika bima ya kitaifa ya afya ya Kenya. Huduma hizi zina uwezo wa kupunguza matatizo ya kiafya yatokayo kwa malaria na SCA.

Katika muda mrefu, tunatumaini kwamba katika siku zijazo habari tuliyojifunza kutoka kwa utafiti huu utawafaidi watoto walio na SCA barani Afrika, kwa kupata dawa bora ya kuzuia malaria.

NINI MBADALA WA KUSHIRIKI KATIKA UTAFITI HUU?

Mtoto wako hahitaji kushiriki katika utafiti huu. Hata kama hautamruhusu mtoto wako ashiriki, yeye bado anaweza kupokea matibabu ya kawaida kutoka kwa watoa huduma ya matibabu wa kawaida.

JE, HABARI YA MTOTO WANGU ITAWEKWA SIRI?

Mtoto wako hatatambuliwa kwa jina, au mambo mengine yeyote ambayo yanaweza kumbainisha katika rekodi za utafiti zitakazoshirikiwa nje ya Hospitali ya Kaunti ya Homa Bay. Kwa rekodi zitakozofichuliwa nje ya Hospitali ya Kaunti ya Homa Bay, mtoto wako atapewa nambari ya kificho ya kipekee. Logi au orodha ambayo ni pamoja na nambari ya kificho na jina la mtoto wako na/au kitambulisho kingine cha kibinafsi ni ya siri na itawekwa salama na timu ya utafiti.

Kila juhudi zitafanywa kudumisha usiri wa rekodi za matibabu za mtoto wako. Hata hivyo, hii haiwezi kuwa ya uhakika. Kuna hatari ya uwezekano wa kupotea kwa usiri.Baadhi ya ofisi na watu wengine zaidi kuliko watafiti wanaweza kuangalia chati ya matibabu ya mtoto wako na rekodi za utafiti.Hawa ni pamoja na watu kutoka:

Timu za utafiti katika Chuo Kikuu cha Moi, Chuo Kikuu cha Duke, na Taasisi ya Utafiti wa kliniki ya Duke;

- Taasisi ya utafiti na kamati ya maadili ya Chuo Kikuu cha Moi na Bodi ya Taasisi ya Kupitia Mfumo wa Afya ya Chuo Kikuu cha Duke;
- Wawakilishi kutoka NIH ya nchi ya Marekani.

Rekodi za utafiti zitahifadhiwa kwa miaka kumi baada ya kukamilka kwa utafiti kwa taratibu za hapa. Habari za utafiti katika rekodi za matibabu za mtoto wako zitawekwa milele.

Wakati wa kipindi cha utafiti huu, daktari wako na timu ya utafiti wanaweza shiriki habari yako ya utafiti na baadhi ya watu. Hawa ni pamoja na:

- Wachunguzi wa utafiti, ambao huthibitisha usahihi wa taarifa;
- Watu wenye asili ya matibabu ambao huamua athari za dawa zitakazokuwa kwa ugonjwa wa mtoto wako; na/au
- Watu ambao huweka pamoja taarifa yote ya utafiti katika fomu ya ripoti.

Wakati mtoto wako yuko katika huu utafiti, habari ya utafiti iliyokusanywa juu yake inaweza wekwa katika rekodi zake za matibabu.

Una haki ya kuacha kushiriki katika utafiti huu wakati wowote. Kuacha kushiriki, ni lazima umjulishe daktari wako wa utafiti kwamba unataka kuacha.Ikiwa unataka mtoto wako ashiriki katika utafiti huu, ni lazma utie sahihi hii fomu ya adhini ili kuwapa timu ya utafiti ruhusa ya kuanagalia taarifa ya afya ya mtoto wako. Ikiwa utakataa kuturuhusu kupitia taarifa ya afya ya mtoto wako, mtoto wako hataweza kuwa katika utafiti. Hata hivyo, kutotia sahihi fomu hii hakutakuathiri wewe au mtoto wako kupata huduma ya matibabu.

Ilhali taarifa inayotokana na utafiti huu inaweza kuwasilishwa katika mikutano ya kisayansi au kuchapishwa katika jarida ya kisayansi, utambulisho wa mtoto wako hautafunuliwa na mtoto wako hatatajwa kwa jina. Maelezo ya majaribio haya ya kliniki yatapatikana kwa <http://www.ClinicalTrials.gov> na sajili ya jaribio za kliniki ya *Pan* Afrika *(http://www.pactr.org*). Mitandao hii haitakuwa pamoja na taarifa inayoweza kukutambua wewe. Kwa ukubwa, mitandao hii itajumuisha mukhtasari wa matokeo.

Rekodi za mtoto wako hazitatumika kwa madhumuni mengine yeyote au kushirikiwa na mtu mwingine bila idhini yako.

GHARAMA NI GANI?

Hakutakuwa na gharama za ziada kwako wewe au mtoto wako kwa sababu ya kuwa katika utafiti huu. Taratibu zozote za utafiti, dawa za utafiti, au vipimo vya utafiti vitatolewa kwa mtoto wako bila malipo.Kwa kuongezea, ikiwa mtoto wako atashiriki katika utafiti, tutamsajili katika Bima ya Kitaifa ya Afya ya Kenya (NHIF) wakati yeye anashiriki katika utafiti. Baadhi ya aina za huduma za matibabu zinaweza kulipwa kwa NHIF.

NINI KUHUSU FIDIA?

Hakutakuwa na fedha au fidia kwa ajili ya mtoto wako kushiriki katika utafiti huu. Hata hivyo, utapokea shilingi 500/= kwa ajili ya kukusaidia kwa usafiri wa ziara za ufuatiliaji zilizopangwa. Hii huenda isifidie gharama kamili ya usafiri, lakini itasaidia kufanya iwe rahisi kushiriki kikamilifu katika utafiti.

JE, KAMA MTOTO WANGU AMEJERUHIWA?

Huduma ya matibabu ya haraka na ya muhimu inapatikana katika Hospitali ya Kaunti ya Homa Bay endapo mtoto wako atajeruhiwa kutokana na kushiriki katika huu utafiti. Hakuna dhamira kutoka kwa Chuo Kikuu cha Moi, Chuo Kikuu cha Duke kupitia Taasisi yake ya Duke ya Utafiti wa kliniki, Hospitali ya Kaunti ya Homa Bay, au NIH kukurudishia pesa au kulipa malipo ya gharama ya jeraha lolote linalohusiana na utafiti, pesa au huduma ya matibabu ya bure.

Kwa maswali yoyote kuhusu utafiti au kuumia kunakohusiana na utafiti, wasiliana na Daktari Njuguna kupitia nambari +XXXXXXXXX wakati wa masaa ya kawaida ya kazi na nambari +XXXXXXXXX baada ya masaa ya kazi, wikendi na likizo.

JE, KUHUSU HAKI ZANGU NIKIMKATAZA MTOTO WANGU KUTOSHIRIKI AU KUJIONDOA KATIKA UTAFITI?

Unawezachagua kutomruhusu mtoto wako asiwe katika utafiti. Pia unaweza chagua kumruhusu mtoto wako awe katika utafiti na unaweza kumwondoa katika utafiti wakati wowote. Kama utamwondoa mtoto wako kutoka kwa utafiti, hakuna habari yoyote mpya kuhusu mtoto wako itakusanywa kwa madhumuni ya utafiti isipokuwa kama mtoto wako ana athari inayohusiana na dawa ya utafiti.Ikiwa athari kama hii itatokea, tutahitaji kupitia rekodi zote za matibabu za mtoto wako. Taarifa yote iliyokusanywa kwa ajili ya utafiti itatumwa kwa mdhamini wa utafiti, ambaye ni NIH ya nchi ya Marekani.

Uamuzi wako kutomruhusu mtoto wako ashiriki au kumwondoa kutoka kwa utafiti hakutahusisha adhabu yoyote, ingawa hautapokea tena huduma ya matibabu au faida zinazotolewa na utafiti na huduma ya mtoto wako itahamishwa kwa madaktari ambao hawashiriki katika utafiti, kama vile daktari wa mtoto wako, hospitali au kliniki. Hii haitakuthuru wewe au mtoto wako kupata huduma ya afya kupitia watoa huduma wa afya wako wa kawaida.

KUONDOLEWA KUTOKA KWA UTAFITI BILA HIARI

Daktari wa utafiti anaweza amua kumwondoa mtoto wako kutoka kwa utafiti ikiwa:

- Hali ya mtoto wako itakuwa mbaya Zaidi , au
- Mtoto wako ana athari mbaya sana, au
- Anahitaji matibabu ambayo hayaruhusiwi katika utafiti, au
- Daktari wa utafiti akiamua si bora kwa maslahi ya mtoto wako kuendelea katika utafiti.

NIH, Daktari Njuguna au madaktari wengine wa utafiti, mashirika ya udhibitishaji wanaweza simamisha huu utafiti wakati wowote bila idhini yako. Ikiwa hili litatokea, utafahamishwa na daktari wako wa utafiti atakueleza kuhusu chaguo zingine.

IKIWA KUNA HABARI MPYA?

Utapewa habari yoyote mpya tutakayojifunza wakati wa kipindi cha utafiti ambayo inaweza kuathiri wewe iwapo utakubali kuendeleza ushirikishi wa mtoto wako katika utafiti.

NITAPIGIA NANI SIMU IKIWA NINA MASWALI AU TATIZO?

Kwa maswali kuhusu utafiti au kuumia kunakohusiana na utafiti au ikiwa una TATIZO, wasiwasi au mapendekezo kuhusu utafiti, wasiliana na Daktari Njuguna kwa nambari +XXXXXXXXX wakati wa masaa ya kazi,baada ya masaa ya kazi, na wikendi na likizo. Kwa maswali kuhusu haki za mtoto wako kama mshiriki katika utafiti, au kuongea kuhusu tatizo, wasiwasi au mapendekezo kuhusiana na utafiti, au kupata taarifa au kutoa mchango kuhusu utafiti, wasiliana na Taasisi ya utafiti na kamati ya maadili ya Moi (IREC) kwa nambari +2540787723677. Bodi ya kupitia mifumo ya afya ya Taasisi ya Chuo Kikuu cha Duke pia imefanya mapitio ya utafiti huu, ikiwa una maswali yoyote ambayo wanachama hao wanaweza kukusaidia tafadhali yaelekeze kwa +1(919) 668-5111.

TAARIFA YA IDHINI

Madhumuni ya utafiti huu, taratibu zitakazofuatwa, hatari, manufaa yameelezwa kwa mtoto wangu na mimi.Nimeruhusiwa kuuliza maswali, na maswali yangu yamejibiwa kwa kuridhika kwangu. Nimeambiwa ni nani wa kuwasiliana naye ikiwa nina maswali, kuongea kuhusu tatizo, wasiwasi au mapendekezo kuhusiana na huu utafiti. Nimesoma fomu hii ya idhini na kukubali kuwa mtoto wangu awe katika huu utafiti, kwa kuelewa kwamba naweza kumwondoa mtoto wangu wakati wowote. Nimeambiwa kwamba nitapewa nakala ya fomu hii ya idhini iliyotiwa sahihi na tarehe.

___________________________________

Jina la mzazi au mlezi wa kisheria lililochapishwa

____________________________________

Sahihi ya mzazi au mlezi wa kisheria Tarehe Wakati: ___: ____

____________________________________ Wakati: ___: ____

Sahihi ya shahidi (ikiwa inafaa) Tarehe

Nimeeleza kwa kikamilifu huu utafiti kama ulivyofafanuliwa kwa fomu hii. Nimejibu maswali ya mzazi/mlezi na nitajibu maswali yoyote ya siku za usoni kadri ya uwezo wangu. Nitaiambia familia mabadiliko yoyote katika taratibu au katika uwezekano wa madhara/uwezekano wa faida za utafiti zinazoweza kuathiri nia ya mzazi/mlezi ya kutaka mtoto wake aendelee kuwa katika utafiti.

____________________________________ ___________

Jina lililochapishwa la mtafiti anayepokea ruhusa au idhini ya mzazi. Tarehe

____________________________________ ___________

Sahihi ya mtafiti anayepokea ruhusa au idhini ya mzazi. Tarehe

SAMPULI ZA DAMU ZITATUMIKA VIPI?

Wakati wa kipindi cha utafiti huu, kwa zaidi ya miezi 12, damu ya mtoto wako itachukuliwa katika kiasi Fulani kilichotajwa hapo juu (katika ziara hii ya kujiunga na kisha kila ziara ya kila mwezi kwa miezi 12). Tutakusanya hii damu ili kuipima mara kwa mara kufuatilia athari za SCA, malaria na dawa za utafiti.

Aidha, sampuli moja ya damu itakusanywa na kutumika tu kwa ajili ya utafiti wa siku zijazo, kama wewe utakubaliana na ombi hili. Sampuli hizi zitahifadhiwa kwa muda mrefu katika moja ya vyuo vikuu au mashirika ya utafiti yanayofanya utafiti huu, ikiwa ni pamoja na Chuo kikuu cha Moi au Chuo Kikuu cha Duke. Sampuli pia zinaweza kushirikiwa na wachunguzi katika taasisi zingine. Matumizi yoyote ya hizi sampuli zilizohifadhiwa lazima iidhinishwe na taasisi ya kupitisha au bodi ya maadili.

Yafuatayo ni mazingatio kadhaa muhimu kwa ajili ya sampuli hizi

- Tutakusanya na kuhifadhi takriban mililita 2.5 ya damu kwa ajili ya utafiti wa siku za usoni wakati wa ziara ambapo tunakusanya damu kwa ajili ya huduma ya kawaida Sampuli hii itatumiwa tu kujifunza malaria, SCA, au magonjwa mengine ya kuambukiza katika watoto walio na SCA.
- Tutahifadhi pia doa la damu katika karatasi maalum kutoka kwa damu tuliyochukua kutoka kwako. Baada ya utafiti kukamilika, hii damu itatumiwa kupima jeni za vijidudu vya malaria.
- Taarifa tutakayoipata kutoka kwa tafiti hizi haitaathiri kuhudumiwa kwa mtoto wako.
- Sampuli hizi hazitauzwa au kutumika kwa uzalishaji wa bidhaa za kibiashara.
- Tunaweza kufanya utafiti kwa jeni za binadamu au malaria katika sampuli hizi, lakini hakuna taarifa itakayowekwa katika rekodi za matibabu za mtoto wako, na sampuli zitawekwa kwa kificho ili kwamba hakuna mtu atakayeweza kumtambulisha mtoto wako.
- Hakuna faida ya moja kwa moja kwa mtoto wako ikiwa utaturuhusu kutumia sampuli kwa matumizi haya. Hata hivyo, kama zinaweza kutumika kwa utafiti wa siku za usoni, tunatumai kwamba zitatusaidia kujifunza jinsi ya kuzuia vyema, kutibu, au kutibu matatizo ya kiafya katika watoto walio na SCA.

Kila jitihada zitafanywa kulinda habari yako ya siri, lakini hii haiwezi kuwa na uhakika. Habari inayoweza kupatikana kutokana na kushiriki kwako katika utafiti wa siku za usoni haitawekwa pamoja na rekodi zako za matibabu.

IKIWA NITAKUBALI KUTOA SAMPULI SASA, NAWEZA KUTOA OMBI KWAMBA SAMPULI ZANGU ZIHARIBIWE KATIKA TAREHE YA BAADAYE?

Ikiwa utaamua baadaye kwamba ungependa sampuli zako ziharibiwe, lazima umuarifu Daktari Njuguna au wawakilishi wake katika timu ya utafiti kwamba unaondoa ruhusa yako kwamba sampuli zako zihfadhiwe na kutumika kwa utafiti wa siku zijazo

- Wakati wa utafiti mkuu, tafadhali wasiliana na mratibu wa utafiti Bwana. Joseph Kipkoech kwa nambari +XXXXXXXXX.
- Baada ya utafiti mkuu, tafadhali wasiliana na Taasisi ya utafiti na kamati ya maadili ya Moi kwa nambari +XXXXXXXXX

Ikiwa taarifa inayounganisha sampuli zako fiche na wewe tayari zimeharibiwa, sampuli zako haziwezi tena kuhusishwa nawe na hatutaweza kuzipata kwa ajili ya kuziharibu.

Pia, hata kama sampuli zako zitaharibiwa:

- Hatuwezi kupata tena sampuli au taarifa ambayo tayari tumeshapatia watafiti.
- Hauwezi kuondoa sampuli yako ikiwa tayari imeshachambuliwa au kupimwa.

Tafadhali tia sahihi kwenye mstari mmoja wa sahihi hapo chini kama unakubali kuwa sampuli zako za damu zihifadhiwe kwa uwezekano wa utafiti wa siku zijazo, au ikiwa haukubali sampuli zako za damu zihifadhiwe.

Ninakubali kuruhusu sampuli zangu za damu zihifadhiwe kwa ajili ya uwezekano wa utafiti wa siku zijazo wa SCA, malaria na maambukizi mengine katika watoto walio na SCA.

Sahihi ya mzazi au mlezi wa kisheria Tarehe

Sahihi ya shahidi (ikiwa inafaa) Tarehe

Mimi sikubali kuruhusu sampuli za damu yangu kuhifadhiwa kwa uwezekano wa utafiti wa siku zijazo wa SCA, malaria, na maambukizi mengine katika watoto walio na SCA.

Sahihi ya mzazi au mlezi wa kisheria Tarehe

## APPENDIX H: INFORMED CONSENT (KISWAHILI TO ENGLISH)

Enrollment Consent to Participate in a Research Study

Title of Study: Enhancing Preventive Therapy of Malaria in Children with Sickle Cell Anemia in East Africa (EPiTOMISE).

Researcher: Dr. Festus Njuguna

We are seeking your consent to allow your child to take part in this research because your child has Sickle Cell Anemia (SCA).

WHAT DO YOU NEED TO KNOW IN GENERAL ABOUT RESEARCH STUDIES?

We are seeking your consent to allow your child to take part in a clinical trial research. Before you consent to allow your child to participate in this clinical trial research study, it is important for you to understand that a research like this includes only those people who choose to participate. Your child’s participation is optional. Please read this consent form keenly and take your time to make your decision. Your child’s research doctor or research assistants will discuss about this consent form with you. Please ask about anything or any information that you do not understand well.

This consent form explains what will happen in this research. The form explains the dangers, problems, challenges and any other thing of importance. It is important that you ask questions and understand what is needed for your child’s participation in this study. We encourage you to discuss with your family and friends before you decide to allow your child to take part in this research. You can go home with a copy of this form to go through as you decide whether you would like your child to participate in the research. If you will agree that your child participates and you sign on this form, you will be given a copy of this form that has a signature and date for you to keep.

Please inform the research doctor or research assistants in case your child is participating in another research.

WHO WILL BE MY RESEARCH DOCTOR?

If you agree to participate, Dr. Njuguna will be your doctor for this research and he will communicate with your normal health officer at all times that you will be in the study and even after, if need be.

The NIH grant will fund this research. Part of Dr. Njuguna’s salary and his research team will be paid from this grant.

WHY IS THIS RESEARCH BEING DONE?

We are seeking your consent for your child to participate in this research because your child is between 1 and 10 years of age, has SCA and lives in a malaria endemic area.

We are looking for the best way to prevent malaria in children with SCA. This research will help us discover the best drugs that can be used to prevent malaria in children with SCA in malaria endemic areas in Africa.

Below is a list of drugs included in this study. In this research, your child will take one out of the following three types of oral drugs to prevent malaria:

- *Proguanil* (also known as *Paludrine*) is the current drug used in Kenya that helps to prevent malaria in children with SCA. Your child could already be using this drug to prevent malaria. This drug is taken daily.

OR

- *Sulfadoxine/Pyrimethamine-Amodiaquine (SP-AQ*) is a combination of drugs that constitute *sulfadoxine* and *pyrimethamine* that are taken at the same time like amodiaquine. These drugs are taken monthly.

OR

- Dihydroartemisinin-Piperaquine (DP) is a combination of dihydroartemisinin and piperaquine.These drugs are taken monthly.

In case your child gets malaria, he/she will be given an oral drug that is called Artemether-Lumefantrine (AL). All the children below 5 years of age will also be given Penicillin orally every day, whereby it is aimed at preventing bacterial infection and is a maintenance dose for children with SCA.

HOW MANY CHILDREN WILL PARTICIPATE IN THIS RESEARCH?

About 246 children will complete this research. The research will take place in Homa Bay county hospital in Homa Bay, Kenya.

WHAT IS ASSOCIATED WITH THIS RESEARCH?

If you agree that your child takes part in this study, you will be asked to sign on this consent form. Your child will go through the following procedures to make sure that he/she is suitable for this study:

Examination Visit

Before enrolling in the research, the research assistants will need to verify if your child is suitable to participate. This helps in improving the safety of participating in the study.

During the examination visit, the following procedures will be performed and recorded:

- We will go through your child’s medical history
- We will go through the drugs that your child is using
- We will perform an electrocardiogram (ECG) test, whereby the electrical activity in your child’s heart is measured through the skin.

We will draw approximately 1milliliters (mL) of blood to measure the red blood cells of your child to see proteins that cause SCA and perform a blood test to determine if it safe to enroll him/her in the research.

Enrollment visit

During the visit for enrollment, the following procedures will be done and the information recorded:

- We will go through and record your child’s medical history, together with any other medical treatment that came up since the previous visit.
- We will record the drugs that your child is using
- We will do physical examination which will include height and weight.
- We will collect a blood sample from a finger prick less than 1/2ml
- If necessary, we will administer to your child a Meningococcal vaccine.  This vaccine will help protect your child against bacteria that causes meningitis. This may not be necessary if your child has already received this vaccine, which is routinely recommended in Kenya.
- We will give you a supply of penicillin for 30 days (if your child is below 5 years of age) which is a drug used to prevent bacterial infection in children with SCA.
- We will register your child with the National Health Insurance Fund (NHIF)
- We will give you drugs for research that will prevent your child from getting malaria and instructions on how to take the drugs.

In this enrolment process also, we will decide on which drugs your child will receive that prevents malaria. To do so, your child, without any order (like getting a number), will receive either *Proguanil* every day, *SP-AQ* every month, or *DP* every month. Your child has a chance of 1 out of 3 of getting a particular research drug.

Whichever drug that your child will receive through this procedure will be the preventive drug for malaria that your child will receive all through the study. All the children will receive a particular type of drug that prevents malaria.

Follow up visits (monthly)

In every monthly visit, the following procedures will be done:

- We will go through your child’s medical history, together with the outcomes of the previous visit
- We will do physical examination including height and weight
- We will draw a sample of blood less than ½ mL from a finger prick. which will be tested for anaemia level and malaria parasites in your child
- If your child has fever or has had a fever within the past 24 hours, he/she will immediately undergo a test for malaria parasites; if your child has not had a fever, then the blood will be preserved for testing at the end of the study.
- Every three months, we will draw approximately 2 ½ ml of blood for frequent/ regular testing.
- On the 6^th^ month and the 12^th^ month which will be the last, we will collect blood samples of 1 ml to check for red blood cells that are sickled.
- If you sign a separate form to consent for additional blood collections for the visits after 3,6,9 or 12 months, we will collect additional 2 ½ml of blood for future research studies.
- If need be, we will do an ECG*.
- We will give you study drugs and prescriptions on how to take them;
- We will give you a 30-day supply of penicillin to be taken daily (if below the age of 5)

*Approximately 20 children will be asked to return to the clinic two days later so as to repeat the ECG test every month during the study; this will be a follow up of ECG results as a sustainable basis through the whole period. This will only be among the children in the DP group of study.

If your child will have a blood test for malaria and it turns out positive, he/she will be treated at that moment with AL.

Emergency Visit (If needed)

In this research, if your child is at home and has a fever either because he/she feels it or the temperature reading is above 37.5 degrees Celsius, we kindly request you to call the research workers so that they can arrange for the child to be seen by one of the doctors. In this process, the following will be done:

- We will go through your child’s medical history including all the results from the previous visit.
- We will do physical examination including height and weight.
- We will draw a sample of blood less than ½ Ml from a finger prick. If your child has fever or has had a fever within the past 24 hours, he/she will immediately undergo a test for malaria parasites; if your child has not had a fever, then the blood will be preserved for testing at the end of the study.

If your child will have a blood test for malaria and it turns out positive, he/she will be treated at that moment with AL.

HOW LONG WILL MY CHILD BE IN THE STUDY?

It is expected that the period of participation in the study will be every month for 12 months. Your child’s participation in the study will end at the last visit at 12 months.

You can choose to stop participation at any point without any penalty. In that state, you will not be able to receive any health services or benefits provided by the study and the services given to your child will be handed over to other doctors who are not in the study like your child’s doctor, hospital or clinic. Even so, we encourage you to speak with your doctor first if you decide to stop participation in the study.

WHAT ARE THE RISKS IN THE STUDY?

Your participation in the study exposes your child to the following risks. If you choose to participate, you are required to discuss these with the study doctor as well as your normal health officer.

It is possible for any drug to cause unwanted side effects. You are required to know about the effects that can arise from this research before you decide if your child will take part. There ca be risks, disruption, drug interactions or other effects that are not known. These risks can be as a result of worsening of the complications of sicke cell anaemia. If these complications are worse they can cause death.

*Proguanil* can cause some, all or none of the effects listed below.

Most likely:

- Nausea Anemia
- Vomiting Allergic reactions
- Stomach ache Depression
- Abnormal dreams Coughing
- Headache Lack of sleep
- Diarrhea Rashes
- Weakness Fever
- Loss of appetite Dizziness
- Skin itching

Less likely:

- Being scared
- Fast, hard or unusual heartbeat
- Mouth sores
- Hair loss
- Severe rash

*SP-AQ* can cause some, all or none of the effects listed below:

Most likely:

- Headache Coughing
- Vomiting Nausea
- Loss of appetite Stomachache
- Diarrhea Weakness
- Fever Fatigue
- Difficulty in sleeping Rash
- Feeling sleepy Scratching/Itching

Less likely

- Eye problems/retinopathy
- Hepatitis
- Anaemia

*DP* can cause some, all or none of the effects listed below

Most likely:

- Headache
- Fever
- Coughing
- Flu
- Diarrhea

Less likely:

- Dizziness
- Vomiting
- Nausea
- Lack of appetite

OTHER RISKS

Your child can feel pain and/ or discomfort during the process of finger prick or drawing blood. Infection, abnormal bleeding, freeze-up and fainting can hardly occur. In order to reduce these risks, we will employ research workers who are qualified and know how to draw blood correctly.

In addition, your child can have unpredictable side effects and problems related to the use of the research drugs. These can combine with possible increase in the complications of sicke cell anaemia which if more serious can be fatal. You should report any problems you may have to the study team.

ARE THERE ANY BENEFITS IN PARTICIPATION IN THE RESEARCH?

If you will allow your child to participate in this research, there can be direct benefits of treatment to your child. The participating children will get study drugs for free that will prevent malaria and bacterial infection, monthly comprehensive evaluation, free malaria test whenever they have fever and registration to NHIF. These services are able to reduce health problems resulting from malaria and SCA.

In the end, we are hopeful that the lessons we learnt from this study will benefit children with SCA in Africa by getting the best drugs that prevent malaria.

BY CHOSING NOT TO PARTICIPATE, WHAT IS THE OTHER ALTERNATIVE?

It is not necessary that your child take part in this study. Even if you do not allow him/her to participate, your child will still get the normal medical care from the healthcare workers.

WILL MY CHILD’S INFORMATION BE KEPT CONFIDENTIALLY?

Your child will not be identified by name or by any other way that will reveal him/her in the study records that will be used outside of Homa Bay county hospital. Your child will be given a code for all records that will be outside of Homa Bay county hospital. The list that includes the code and your child’s name and/or any other personal identity is a secret and will be kept safe by the study team.

We will put all effort to maintain confidentiality of your child’s medical records. However, this cannot be guaranteed. There is a risk of breach of confidentiality. People in other offices other than the study team can look at your child’s medical charts and study records. These includes; research teams from Moi University, Duke University and Duke Medical Research Institute

- Moi University – Institutional Review and Ethics Committee and Duke University’s Institutional Review Board.
- Representatives from NIH in USA

Study records will be kept for 10 years after completion of the study as per the procedure here. Your child’s research information in the medical records will be kept forever.

During the period of this study, your doctor and the research team can share your research information with other people including:

- Research investigators who confirm the accuracy of the information
- Individuals with medical backgrounds who know the effect that the drugs have on your child’s disease; and/or
- People who put together all the research information in the report form.

At the time that your child will be in this study, any research information relating to him/her can be put in his/her medical records.

You have the right to stop participation in this research at any point. You must inform your research doctor when you want to withdraw from the study. If you want your child to take part in this study, you must sign on this consent form to allow the research team to look at your child’s information. If you will not allow us to go through your child’s health information, then your child will not be in the study. However, your not signing on the form will not prevent you or your child from getting health services.

While the information resulting from this study can be presented in scientific conferences or published in scientific journals, your child’s identity will not be revealed and will not be mentioned by name. Explanations of these clinical trials will be found in [*http://www.ClinicalTrials.gov*](http://www.ClinicalTrials.gov)and registers of clinical trials of Pan Africa (<http://www.pactr.org>). These websites will not have information that can identify you. In general, these websites will include summary of findings.

Your child’s records will not be used for other purposes or shared with any other person without your consent.

WHAT IS THE COST?

There will be no additional costs on you or your child because of being in this research. Any study procedure, study drugs or tests will be provided to your child at no charge. In addition, if your child will participate in this research, we will enroll him/her to NHIF during the period that the child will be in the study. Some of the health services can be paid through NHIF.

WHAT ABOUT COMPENSATION?

There will be no funds or compensation for your child’s participation in this study. However, you will receive Ksh. 500 to help you travel for follow-up visits as planned. This may not completely compensate for your travel cost, but it will help to make it easier for you to participate in the study.

WHAT IF MY CHILD HAS BEEN INJURED?

Health emergency services are available at Homa Bay county hospital whenever your child is injured while participating in this research. There is no commitment from Moi University, Duke University through its medical research institute, Homa Bay county hospital or NIH to give you back money or pay for any injury costs related to the study or to give you free treatment.

For any questions about the study or injury related to the study, get in touch with Dr. Njuguna through this number +XXXXXXXXX at normal working hours and this number +XXXXXXXXX after working hours, on weekends or public holidays.

WHAT ABOUT MY RIGHTS WHEN I DO NOT ALLOW MY CHILD TO PARTICIPATE OR WITHDRAW FROM THE STUDY?

You can decide not to allow your child to participate in the research. You can also allow your child to be in the study and you can remove him/her from the study at any time. If you will remove your child from the study, there will be no more collection of new information about your child for purposes of the study unless if your child has side effects related to the study drugs. If such effects come up, we will need to go through all of your child’s medical records. All information collected for this study will be sent to the funding agency that is NIH in the USA.

Your decision not to allow your child to participate or withdrawal from the research will not result in any punishment although you will no longer receive medical services or benefits given by the study. Services to your child will be transferred to other doctors who are not in the study like your child’s doctor in the hospital or clinic. This will not affect you or your child from getting health services from your normal health service provider.

REMOVAL FROM THE STUDY INVOLUNTARILY

The research doctor can decide to remove your child from the study if:

- Your child’s condition will be worse, or
- Your child has very bad side effects, or
- Requires treatment that is not allowed in the study, or
- If the study doctor decides that it is not in your child’s best interest to continue in the study

NIH, Dr. Njuguna or any other study doctors and regulatory agencies are able to stop this research at any time without your consent. If this happens, your study doctor will inform you and he will explain to you about other options.

WHAT IF THERE IS NEW INFORMATION?

You will be informed of any new thing that we will learn during the period of study that can affect you in case you will agree that your child continues participation in the study.

WHO CAN I CALL WHEN I HAVE A QUESTION OR PROBLEM?

For questions about the research or injury related to the study or if you have a problem, worries or recommendations about the research, get in touch with Dr. Njuguna on this number +XXXXXXXXX during working hours, after working hours, on weekends and public holidays. For questions about your child’s rights as a participant in the study, or to talk about any problems, worries or recommendations about the research, get in touch with Moi’s Institutional Review and Ethics Committee (IREC) on this number: +254787723677. Duke University’s Institutional Review Board of health systems has also done a review for this study and if you have any questions that the members can help you with, please direct them to +19196685111.

STATEMENT OF CONSENT

The purpose of this research, procedures to be followed, risks and benefits have been explained to my child and I have been allowed to ask questions, and the questions have been answered to my satisfaction. I have been told whom to communicate with if I have questions, problems to discuss, worries and recommendations related to this study. I have read this consent form and agreed that my child be in this research, with the understanding that I can remove my child at any time. I have been told that I will be given a copy of this consent form that has a signature and date.

___________________________________

Name of parent or legal guardian

____________________________________ ___:___

Signature of parent or legal guardian Date Time

____________________________________ ______________________________

Signature of Witness (if applicable) Date Time

I have explained this study in detail as outlined on this form. I have answered the questions from the parent/guardian and I will answer any future questions to my best. I will tell the family about any changes to the procedures or potential hazards/benefits of the study that can affect the interest of the parent/guardian to want to have the child continue with the study.

____________________________________ ___________

Name of the researcher receiving permission or approval from the parent Date

____________________________________ ___________

Signature of the researcher receiving permission or approval from the parent Date

HOW WILL THE BLOOD SAMPLES BE USED?

During this study period of over 12 months, your child’s blood will be taken in quantities as mentioned above (in the enrollment visit and thereafter monthly for 12 months). We will collect this blood so as to test regularly to check on the effects of SCA, malaria and the study drugs.

If you will agree, one blood sample may be collected and used by the study at a later date. These samples will be stored for a long period of time in one of the universities or institutions of research conducting this study, including Moi University and Duke University. The samples can also be used by researchers from other institutions. Any use of these preserved samples must be authorized by the institutional review board.

The following are some important factors about these samples:

- We will collect and preserve approximately 2.5ml of blood for future research use during the visits which we will collect blood for routine care.. This sample will only be used to learn about malaria, SCA, or any other infectious diseases in children with SCA.
- We will also preserve a drop of blood on a special paper from the sample of blood we took from you. This blood will be used to test microbial genes of malaria after the research is finished.
- The findings from this study will not affect the services given to your child.
- These samples will not be sold or used for production of goods for business.
- We can use this samples to do research on human genes or malaria, but no report will be put in your child’s record, and the samples will be coded so that no one can identify your child.
- There will be no direct benefits to your child if you will allow us to use the samples for this purpose. However, if they can be used in future research, we hope that they will help us learn how to properly prevent, treat and manage health problems related to children with SCA.

All effort will be put to protect your confidential information, but this cannot be guaranteed. Any information related to your future participation in the research will not be put together with your medical records.

IF I AGREE TO GIVE MY SAMPLE NOW, CAN I REQUEST THAT MY SAMPLE BE DESTROYED AT A LATER DATE?

If you decide later that you want your sample to be destroyed, you must inform Dr. Njuguna or his representatives in the study team that you are withdrawing and that your sample will not be preserved and used by the study in future.

- At the time of the main study, please communicate with the study coordinator Mr. Joseph Kipkoech on this number - +XXXXXXXXX.
- After the main study, please communicate with the Moi Institute of Research and Ethics Committee on this number - +2540787723677.

If the report linking your coded sample to you has been destroyed, then the sample cannot be linked to you and we cannot find them since they are destroyed.

Also, even if your samples will be destroyed:

- You cannot get the sample or report that we have already given to the researchers/investigators.
- You cannot remove your sample if it has already been analyzed or tested.

Please sign on one line below if you agree to give your sample of blood to be preserved for future use by the study, or if you disagree.

I agree to give my blood sample to be preserved for future study testing for SCA, malaria and any other infection in children with SCA.

Signature of parent or legally authorized representative Date

Signature of Witness (if applicable) Date

I do not agree to give my blood sample to be preserved for future study testing for SCA, malaria and any other infection in children with SCA

Signature of parent or legally authorized representative Date

## APPENDIX J: INFORMED CONSENT (DHOLUO)

Wi Nonro: Choro nyime chenro mar geng’o malaria e nyithindo mankod tuo mar remo ei Afrika mamilambo.

Janonro: Daktari Festus Njuguna

Ikwayi mondo iyie ni nyathini obed enonroni nikech nyathini nigi tuo mar remo.

GIN GIGO MAGE MOROMO ING’E ENONRO?

Ikwayi mondo miiyie ne nyathini obedie enonro mar thieth. Kapok iyie mimondo nyathini obed enonro mar thieth ni, ber mondo mi ing’e ni nonro kaka magi dwaro mana jogo moyiero bedo eachiel. Bedo mar nyathini en mana kachiuruok. Koyie some otasni eyomakare to ikau thuoloni mar yiero pachi. Daktach nyathini mar nonro kata jalo matimone nonro bowuoyo ewi otasni kachiel kodi. Koyie penje ewi wach moramora ma okiwinj maler.

Otasni nyiso matut gimabiro timore enonroni. Otasni nyiso matut ewi ahinye, gigo mamiyi pek, winjo marach, kod weche moko mapek. Ber mondo mi ipenj penjo kendo iwinj makare gigo maidwaro enonroni. Wajiwi mondo mi ilos gi anyuolani kata gi osiepeni kapok iyie mimondo nyathini odonj enonroni. Inyalotero otas achiel mar wachni edala kapodi iparo kabe nyathini onego donj enonroni. Kaponiyie mimondo nyathini oyie odonj enonroni to ogoyo seyi eotasni, ibomiyi otas mar wachni mogoye seyi kachielgi tarik mimondo ikan.

Koyie nyis dactari mar nonro kata ng’at matimo nonro kopo ni nyathini nitiere enonro machielo.

NG’ANO MABOBEDO DAKTARINA MAR NONRO?

Kapo ni iyie donjo e nonro, daktari Njuguna bobedo daktachi enonro to obobedo koloso gi jalno marango ngimani endalo duto ma ibobedo enonro to kata bang’e kapo ni idwaro.

Chiwo kowuok kar puonjre mar United States National Institute of Health (NIH) bo chung’ne nonroni. Kanmoro mar daktari Njuguna kod joge mag timo nonroni pesagi ibiro chul kod chiwoni.

ANG’O MOMIYO ITIMO NONRONI?

Ikwayi ni mimondo iyie nyathini odonj enonroni nikech en ekind higni mar 1 kod 10, en kod tuo mar remo, to odak e aluora ma malaria ng’enye.

Wadwayudo yore mabeyo mag geng’o malaria kuom nyithindo mankod tuo mar remo. Nonroni bomiyo wang’e kit thieth maber ma inyalo tikodo egeng’o malaria kuom nyithindo mangi tuo mar remo ekuonde mag Afrika man kod malaria.

Enonroni wadhi pimo kind yiedhi adek mag malaria mabede yudore e ute yiedhe mi mondo wane ni thieth mane kaponi nitie moramora ber eduoko chien kuan mar ndalo ma nyathini bedo gi malaria.

Thieth mag nonro manitie e nonroni gin mochiu piny gi. Enonroni nyathini birokawo thieth achiel kuom thieth adekgi kod dhoge kaka yoo mar geng’o malaria:

- Proguanil (mabende ong’e kaka Paludrine) enie sani rang’iny mamalo mar geng’o tuo mar malaria e nyithindo mankod tuo mar remo ei Kenya. Sechemoko nyathini osegabet nimuonyo yadhni mimondo ogeng’ malaria. Yadhni imuonyo pile.

KATA

- Sulfadoxine/Pyrimethamine-Amodiaquine (SP-AQ) en riuruok mar yiedhe moting’o sulfadoxine kod pyrimethamine mimuonyo karachiel kaka amodiaquine. Yadhni imuonyo edwe.

KATA

- Dihydroartemisinin-Piperaquine (DP) enriuruok mar yiedhi mar dihydroartemisinin kod piperaquine. Yadhni imuonyo edwe.

Kamedo, kapo ni nyathini oyudo malaria, ibiro thiedhe gi dhoge gi yath ma iluongo ni Artemether-Lumefantrine (AL). Nyithindo duto man piny mar higni 5 bende boyudo penicillin pile ka pile eyor dhok ma idwaro ni ogeng’ landruok mar bakteria to enyie rang’iny mamalo kuom nyithindo mankod tuo mar remo.

NYITHINDO ADI MABOBEDO ETIMO NONRONI?

Maromo nyithindo 246 botieko nonroni. Nonroni ibirotimo e osubtal mar County ma Homabay ei Homabay, Kenya.

ANG’O MA IDWARO ETIMO NONRONI?

Kaiyie mimondo nyathini odonj enonroni, ibokwayi mimondo igoseyi e otasni. Nyathini bokalo e okengegi mimondo onyis ni nyathini oromo enonroni.

Pimo elimbe

Kapok idonjo enonro, janonro nyaka ng’e kapo ni nyathini owinjore donji. Maye konyo e okang’ mar donjo enonroni.

Epimo mar limbe, okangegi ibiroluwo to kendo ndiko piny gigo duoko mabiro yudore:

- Wabiro nuoyo rang’o chal mar ngima nyathini
- Wabiro nuoyo rang’o yiedhi ma nyathini tiyogo
- Wabotimo electrocardiogram ma gocho mar dhochuny nyathini ipimo edende gi rapim
- Wabokawo maromo milliliter3.5 mar remb nyathini mimondo wapim bath remo malosore ei odhundo makowo muya kendo golo muya maricho mare nikech proteins makelo tuo mar remo Kendo timo pim ma kinde ka kinde mar remo mondo one kowinjore donjo e nonro

Donjo ni mokuong’o

E seche mar ndiko jii ma okwongo’, yore ma chalo kame ibiro luu bange kae to bange’ weche ibiro maki:

- Wabiro ng’iyo kod ndiko piny duoko mar nyathini kaka osebet e hospital, nyaka duoko ma osebedo ka timre chakre biro ne mokuongo.
- Wabiro ndiko yedhe ma nyathini osetiyo go.
- Wabotimo nono del mar del, ka achiel gi bor ne gi pek ne.
- Wabirokao remo koae lith lwet nyathi maok rom ½ ml.
- Ka nyalore, wabomiyo nyathini chanjo mar Meningococcal. Chanjoni bokonyo nyathini mondo kik yud bakteria makelo meningitis. Mae ok ochuno ka bed ni nyathini oseyudo chanjoni, ma owach e Kenya.
- Wabiro miyo u penicillin kuom odiechienge’ piero adek (ka nyathini pok ochopo higni abich), ma en chendro mar thieth ma itiyo go mondo ogeng’ bacterial infections kuom nyithindo man tiere gi tuo mar remo.
- Wabondiko nyithindu e Kenyan National Insurance Fund

(NHIF);

- Wabiro miyo u kaka nyathini inyalo kingo kod kaka inyalo kawe.

Kod chendro mar limbe ni, wanong’eyo ni en nonro mane mar malaria ma nyathini biro yudo. To mondo mano timre, nyathini ibiro mana mi (kwanyo nembni) modo giyud Proguani pile, SP-AQ e due, kata DP e due. Nyathini ni gi **atamalo mar** achiel kuom adek mar yudo yath ma ochan. Yath moro amora ma nyathini onego oyud e nondro ni biro bedo mar kingo malaria ma giyudo e nondro no. Nyithindo tee boyudo aina moro magengo malaria biro yudo yath machalre.

Limbe ma iluo (e due)

E limbe mar due ka due, magi e gik ma ibiro luu:

- Wabiro nono chal mar nyathini mar hospital, ka achiel gi weche mek thieth ka lure gi limbe ge ma ogik.
- Wabotimo penj, ka achiel gi pek mare gi bor ne.
- Wabiro kao remo ka ochuo lwete ma ok kal ½ ml maa ibiro tiyo go epimo romb rumo remo kod kute makelo malaria
- Ka nyathini ni gi liet, kata ka osebet gi liet ma okchop seche 24, obobedo gi pim mar malaria sano sano; ka nyathini pok ne dende ochwakre ga, koro rembe ibiro kan mar pim ka nondro orumo.**Bang thuolo mar duech a dek ka dueche adek**, wabiro kao gimoro 2 ½ ml mar remo mar pim.
- Ka ochopo due mar auchiel e limbe kod due mae apar gi ariyo, wabiro kao remo moro ma 1 ml ma ibiro pim ne tuo mar remo.
- Ka iketo sei e oboke mar yie machielo kendo, ka iyie ni remo inyalo kaw kendo e limbe dweye mar 3, 6, 9, kata 12 wabro kao kendo 2 1/2 ml mar remo ne nonro ma biro mbele
- Ka ochuno,wabiro timo ECG.
- Wabiro miyo u nondro mar thieth kod kaka itiyo kode.
- Kuom odiechieng’e piero adek, wabiro miyo u penicillin pile (ka pok ochopo higni abich)

Nyithindo maromo 20 ibokwa mondo odok e klinik band odiechienge ariyo mondo onuo ECG ma itimo due ka due seche mar nondro; mae ibiro tim mondo oyud duoko mar ECG mar chendro ma dhi mbele kuom seche go tee. Mae bobedo mana kuom nyithindo ma oyier kod DP mar nondro.

Ka nyathini oyud ni nitiere gi malaria bang’ pim, ibiro thiedhe seche mar AL.

Limbe ma opoya (Kaka dwarre)

Ka lure gi nonro, ka nyathini mantie ot dende ochwakre, nyalo bedo ni giwinjo liet kata nyalo nikech liet mar del osepim ma okalo 37.5, wakwayo ni ugochne jatend nonro mondo opang kaka inyalo kony gi jik ma timo nonro. E limbe ni, gik ma chalo kamae ibiro luu:

- Wabiro nono chal mar nyathini mar hospital, ka achiel gi weche mek thieth ka lure gi limbe ge ma ogik.
- Wabotimo penj, ka achiel gi pek mare gi bor ne.
- Wabiro kao remo ka ochuo lwete ma ok kal ½ ml.
- Ka nyathini ni gi **liet**, kata ka osebet gi **liet** ma okchop seche 24, obobedo gi pim mar malaria sano sano; ka nyathini pok ne dende ochwakre ga, koro rembe ibiro kan mar pim ka nondro orumo.

Ka remb nyathini oyud ni ni gi malaria, ibiro thiedhe e saa ma AL.

Nyathina biro kao seche adi e nonro?

Onego bed ni okao seche ma room dueche apar gi ariyo to itime due ka due. Nonro mar nyathini biro rumo bang’ due mar apar gi ariyo bang’ limbe.

Inyalo yiero weyo bedo e nonro saa moro amora maong’e saa ma ibong’adi. Kuom mano, okibochak iyud thieth moro amora kata ber ma nonro chiwo, to nyathini be ibiro ter laktar ma ok oriwre gi nonro, kaka laktar mar nyithindo matindo, osipital kata klinik. To be kata ka iseyiero ni okidwa riwri e nonro ni, wanyalo nyisi ni ikuong’ iwuo gi laktar mari mondi.

**Ang’o ma en ahinye mar nonro?**

Ka lure kod kaka isebedo kanyakla e nonro ni, nyathini yalo bedo gi gik ma nyalo wuok bange’. Ka iyiero ni idwaro riwri, onego obed ni iwacho weche gi laktar kod nga’t ma ngiyo ga chalni ma konyi ga.

Nyalre ni mondo omi yath moro amora nyalo bedo gi gima wuok bange. Onego bed ni ingeyo gik manyalo wuok mayalo biro e nonro ni ka pok iyie ka nyathini nyalo riwre. Gik machalo kaka ahinye, winjo marach, yedhe ma okwinyre gi del, kata gik moko mayalo wuoke ma kiaree. Andong’egi nyalo riwo koda ka medruok marach moloyo mar midekre motudre gi tuo mar remo. Midekre marach gi ka rach moloyo to, ngima nyalo lal.

Proguanil nyalo omo moko, magi tee kata oknyal omo gik ma nyalo wuok ma ondk piny kae.

Machalo kama:

- Chuny ma wire nyamuoda
- Ng’ok
- Ich kach
- Lek ma ok beyo ahonda
- Wich bar Puodruok
- Diep Pudhno
- Yom Del moolre
- Ketho dhok Wich marundre
- Guon ruok

Ma ok nyal timo ahinya

- Buogrok
- Piyo, matek, kata adundo magocho mok kare
- Adhola mar dhok
- Wich ma lal
- Pudno marach

SP-AQ nalo kelo gikmoko, tee kata mok achel ma kelo chanroke mo ket pin gi.

- Ma nyalo bedo ahinya:
- Wich bar Ahonda
- Ng’ok Chuny ma wire
- Dhok marach Ich kach
- Diep Yom yom
- Del maolre Olo
- Chandruok e nido Del ma pudno
- Tho nido Ilo

Ma ok nyal timo ahinya

- Chandok mar wang/tuo wang
- Chandork mar chuny/ tuo chuny
- .Rumo mar remo

DP nyalo kelo moko, tee kata tee kata mok achel ma kelo chanroke mo ket pin gi.

Ma nyalo bedo ahinya:

- Wich bar
- Del maorle
- Ahoda
- Yamo
- Diep

Ma konyalo bedo ahinya:

- Wich ma luore
- Ngok
- Chuny ma wire
- Dhok ma kethre

AHINYE MOKO:

Kak duoko mar golo remo kuom lut nyathini kata chonyolich, mar nyathini nyalo yudo okobibedo gi kue matin kata kol matin. Yamo, golo remo magen, poto mar lemo kata podho be nyalore, kata gik mok nyare. Duok piny nyalo mar yudo gik ma hinyo, wa diko mana gi mo puojire gi golo remo makare.

Ewi mano, yathini nyalo bedo gi ahinye kod gik ma ok winjre ma oluwore gi tiyo gi yedhe mag nonro ma nyalo ting’o medruok marach mar midekre e tuo mar remo, ma karach, ngima nyalo lal. Onego iwach chandruok moramora ma ingo ne jotim nonro.

BE NITIERE BER E BEDO KAKA JA TIM NONRO?

Ka iyie mar weyo nyathini mondo obed e nonro ni, Nyathini nyalo yudo ber mar thieth, Nyithindo ma odonjo e nonro biro yudo thieth ma nono mondo ogeng’ malaria kod kute ma makelo tuo, rengo matut mar dwe ka dwe, pim ma nono mar kute mag malaria ka gin gi liet mar del kendo donjo e Kenyan National Hospital Insurance Fund. Kony gi nigi nyalo mar duoko chien chandruok mag thieth ka oa kuom malaria kod SCA.

kuom ndalo mang’eny, wageno ni ndalo mabiro weche ma ji opuonjore kuom nonro ni biro konyo nyithindo ma nie Africa gi SCA ka iyudo thieth ma ber mar geng’o malaria.

YORE MAGE MAMOKO MA NITIERE KA MAR BEDO E NONRO NI?

Ok ochuno ni nyathini nyaka bed e nonro ni. Kata ka ok iyie ne nyathini mondo otime, pod onyalo yudo thieth ma kinde ka kinde ka owuok kuom jo chiw thieth ma kinde ka kinde.

BE WECHE NYATHINA IBIRO KAN E YO MA OPANDI?

Nyathini ok bi hul gi nyinge kata wach moramora ma biro chome e andike mag nonro ma ioro ne joma ni oko mar kar thieth mar Homa Bay County. Ne andike ma wuok oko mar kar thieth mar homa Bay county, nyathini ibiro mi namba ma opondo. Otas ma oting’o nembni ma opondo kod nying nyathini gi/kata weche mamoko ma wuoyo kuome opandi kendo ibiro kan kama irito gi oganda ma timo nonro.

Teko duto te ibiro ti godo mondo ondike mag nyathini mag thieth osik e yo ma opondo. Kata kamano, ma ok nyal yie. Nitiere ahinya mar fwenyruok mag weche ma opandi gi nyalore. Ofise moko gi jok mamoko ma opogore gi jo nonro nyalo neon goro mag thieth mag nyathini kod andike mag nonro. Ma oting’o ji ma wuok:

- Jo nonro man e mbalariany mar moi, mbalariany mar Duke, kod kar nonro mar thieth mar Duke;
- Kar nonro mar mbalariany mar Moi kod Ethics Committee and the Duke University Health System Institutional Review Board;
- Jok ma ochung’ ne NIH ma wuok e piny amerika.Representatives.

Andike mag nonro biro ibiro kan mar higni apar bang’ nonro kaluwore gi chike ma piny. Weche mag nonro ma ni e andike mag thieth mag nyathini ibiro kan nyaka chieng.

E saa ma nonro ni dhi mbele, ja thieth mari kod jo tim nonro nyalo ng’iyo weche mag nonro kanyakla gi jok mamoko. Ma nyalo riwo:

- Jok marango research, marango adieri mar wach;
- Jok man gi weche manyiso chakruok mag thieth ma hulo chandruok ma yedhe kelo kuom tuo mar nyathini kod/kata.
- Jok maketo wech te mag nonro kanyakla e yor andike.

E saa ma nyathini ni e nonro ni, weche mag nonro ma okaw kuome inyalo ndiki e andike mare mar thieth.

In gi ratiro mar weyo bedo e nonro ni samoramora. Mar mondo iwe, onego inyis ja thieth mari mar nonro ni idwa weyo. Ka idwaro mondo nyathini odonji e nonro ni, nyaka igo sei e andike ni manyiso ni iyiene mar mondo mi jo kanyakla ma timo nonro thuolo mar ng’iyo ngima nyathini korka thieth mare. Ka itamori yienwa mondo wang’i ma tut weche mag ngima nyathini korka thieth to nyathini ok binyalo bedo e nonro.Kata kamano weyo mar goyo sei e andikeni ok bi chandi kata nyathini e yudo kony mar thieth.

E saa ma duoko ma oa kuom nonro inyalo ter e nyim bura matimo nonro kata ondiki e oboke mag jo nonro, gima wuoyo kuom nyathini ok bi nyisi kendo Nyathini ok bi luong gi nyinge. Weche ma wuoyo kuom thieth biro yudore e <http://www.ClinicalTrial.gov> kod pan african clinical trials registry (<http://www.pactr.org>). Mbui ni ok bi ting’o weche ma nyalo fuli. Di mang’eny, mbui biro ting’o duoko e yo ma chiek. Inyalo manyo mbui gi saa moramora.

Weche nyathini ok bi ti godo e yo moramora kata nyiso ng’atang’ata ka ok ichiwo thuolo.

NENGO GIN ANG’O?

Onge nengo ma biro medore ne in kata ne nyathini kuom bedo e nonro ni. Yor nonro moramora, yedhe mag nonro kata pim mag nonro ibiro chiw ne nyathini nono ma onge chudo. Ewi manos, ka nyathini obedo e nonro, wabiro piko nyathini e NHIF mar Kenya ka gin e puonj. Gund thieth mamoko inyalo chul gi NHIF.

TO CHUDO?

Onge pesa ma biro bedo kata chudo ne nyathini kuom bedo e nonro. Kata kamano ibiro yudo omenda mar Kenya maromo mia abich mar konyi e yor wuoth mar lao chienro magi ma idhie. Ma samoro ok nyal romo nengo te mar wuoth to biro konyo kete ma yot mar bedo enonromalong’o.

TO KA NYATHINA OHINYORE?

Thieth ma nyalo duarore e sechego yudore e kar thieth mar Homa Bay county ka dibed ni nyathini ohinyore nikech bedo e nonroni. Onge andika moro gi mbalariany mar Moi, mbalariany mar Duke kokalo kuom kar puonjruok mar nonro mar thieth mar Duke, kar thieth mar Homa Bay county kata NIH mondo ochiw chudo moramora kata chudo mar hinyruok moramora ma otudore gi nonro, pesa kata thieth ma nono.

Ne penjo kuom nonro kata hinyruok ma otudore gi nonro, tudri gi Dr. Njuguna e +XXXXXXXXX e seche mag tich kod e +XXXXXXXXX bang’ seche mag tich kod giko juma kod ndalo yweyo.

TO RATIRO MARA MAR TAMO NYATHINA DONJO KATA WUOK E NONRO?

Inyalo yiero mar tamo nyathini bedo e nonro. Bende inyalo yiero mar yie ne nyathini mondo obed e nonro kendo inyalo gole e nonro samoramora. Ka igolo nyathini e nonro, onge weche manyien kuom nyathini ma ibiro kaw mag nonro mak mana ka nyathini nigi chandruok ka otudore gi yedhe mag nonro. Ka chandruok ma kama otimore, wanyalo dwaro mondo wachak warang andike mag nyathini mag thieth te. Weche te ma oyudi mag nonro ibiro or ne jatelo mar nonro ma en NIH mar America.

Yiero mari mar tamo nyathini mondo kik donji kata wuok e nonro ok bi kelo kum moramora, kata kamano ok ibi yudo thieth moramora kata ohala ma nitie ei nonro kendo rit mar nyathini ibiro ter ne jo thieth ma onge e nonro kaka jathieth mar nyithindo kar thieth maduong’ kata matin. Ma ok bi moni kata nyathini yudo rit e yor thieth kuom dakteche gi ma kinde ka kinde.

GOLO NG’ATO E NONRO GI ACHUNE.

Ja thieth mar nonro nyalo ng’ado mar golo nyathini oko mar nonro ka: ngima mar nyathini ni e okang’ marach, kata. Nyathini obedo gi chandruok ka oa kuom gik ma itiyogo, kata dwaro thieth ma ok dwarore e nonro, kata Jo thieth mag nonro ong’ado ni ok en dwaro maber moloyo mar nyathini mar dhi mbele.

NIH, Dr. Njuguna kata jothieth mag nonro ma moko, kata oganda ma keto chuke nyalo chungo nonro ni samoramora ka ok ing’eyo. Ka ma otimore, ibiro wachni kendo ja thieth mar nonro ma mari biro wuoyo kodi kuom yore ma moko.

TO KA NITIE WACH MA NYIEN?

Ibiro miyi wach moramora manyien ma ji opuonjore e seche mag nonro ma nyalo chandi kata iyie mar dhi mbele gi bedo mar nyathini e nonro.

NG’A MA ANYALO GOCHONE KA AN GI PENJO KATA CHANDRUOK?

Ne penjo man kuom nonro kata hinyruok ma otudore gi nonro kata ka in gi chandruok, wach moramora kata paro kuom nonro, tudri gi Dr. Njuguna e +XXXXXXXXX e seche mag tich, bang’ seche mag tich kendo nyaka giko juma kod ndalo yueyo. Ne penjo kuom ratiro mag nyathini kaka jatim nonro, kata wuoyo kuom chandruoge, weche ma in go, kata paro ma otudore gi nonro, kata yudo weche moko kata wuoyo kuom chandruoge, dwaro, kataparo ma otudore gi nonro, kata yudo weche moko, kata chiwo kony ne nonro, tudri gi jo bura mar nonro e wi timbe ma opuodhi mar Moi e +2540787723677. Jogo ma puodho weche mag ngima man e mbalariany mar Duke bende oseng’iyo puojni, ka in gi penjo moramora ma jo kanyo go nyalo konyo go akwayi ni kel gi e +1(919) 668-5111.

WACH MAR YIE

Dwaro mar nonro ni, yore ma iluwo,ahinye, kod berose ler ne nyathina kod an. Oyiena mondo apenj penjo kendo penjoga ose duoki e yo ma adwaro. Osenyisa ng’ama agochone ka an gi penjo, wuoyo kuom chandruok, dwaro, kata paro ma otudore gi nonro ni. Ase somo andike mayiena kendo yie mondo nyathina obed e nonro ni, kod winjo ni anyalo golo nyathina samoramora. Osenyisa ni ibiro miya andike mar yie machielo maket, ma ogoe sei kendo ondike tarik.

___________________________________

Nying janyuol ma ogo chapa kata jar it ma opuodhi.

____________________________________

Sei mar janyuol kata jar it ma opuodhi. Tarik Saa: ___: ____

____________________________________ Saa: ___: ____

Sei mar janeno (ka dwarore) Tarik

Ase lero malong’o nonro ma owuoe gi andike ni. Ase dwoko penjo mag ja nyuol/ja ritkendo abiro duoko penjo moramora ma biro mbele maber kar nyalona. Abiro nyisojo ot kuom lokruok moramora e yore kata e ber ma nyalore mag nonro ma nyalo chando gombo mar janyuol mar miyo nyathine mondo obed e nonro.

____________________________________ ___________

Nying jatim nonro ma kwayo Tarik

Mondo janyuol oyie ne.

_____________________________________ ___________

Sei mar jatim nonro ma kwayo Tarik

Mondo janyuol oyie ne.

REMO MAR RANYISI IBIRO TIM GO ANG’O?

E kinde ma nonro dhi nyime, ma ohingo dweche 12 nyathi biro kao remo e kiwango ma owachi malo kanyo ( ka idonjokendo kaibiro bng’ dweche 12). Wabiro kao remo ni mondo watim pim ma pile mondo wang’i duoko mar SCA, tuo ma ikelo gi suna kod thieth mag punj. Ewi mano, remo mar ranyisi moro ibiro kaw kendo kendo ti godo mana e nonro ma biro mbele,ka iyie gi kwayoni. Gik ranyisi gi ibiro kan kuom ndalo mang’eny e achiel kuom mbalariany kata kuonde riwruok mag timo nonro ma timo nonro ni, ka oting’o mbalariany mar Moi kata mbalariany mar Duke.Ranyisi gi bende inyalo tang’ne jotim nonro e kar puonjruok mamako. Tich moramora ma itimo gi gik ranyisi ma okan gi nyaka puodhi gi jok ma opuodhi ma nono kata jo bura ma puodho timbe.

Magi e moko matin kuom gik ma nigi nengo ma ing’iyo kuom gik ranyisi gi:

- Wabiro choko remo manyalo romomilliliter ariyo gi nusmondo okan ne nonro mabiro mbele elimbe ma ikawe remo ma kinde ka kinde mar rit makare. Ranyisi ni ibiro ti godo mana e nonro mar malaria, SCA, kata touché ma makoji mamoko kuom nyithindo man gi SCA.
- Bende wabiro kano remo ma ondong’ e kalatas ma opuodhi ka oa kuom remo ma wayudo kuomi. Ka nonro oserumo, ma ibiro ti godo mar pimo kute ma kelo malaria.
- Weche ma wayudo kuom nonro gi ok bi chando rit mar nyathini.
- Ranyisi gi ok bi usi kata tiyo godo mar loso mwandu mamoko mag ohala.
- Wanyalo timo nonro kuom dhano kata koth malaria ma nitiere e mag ranyisi gito onge wachmoro maibiro keti e andika mag thieth mag nyathini kendo ranyisi gi ibiro mi number ma opondo mondo ng’ato kikng’e gik ma hulo nyathini.
- Onge ber ma aching’ ma nyathini yudo ka iyienwa mondo wati gi ranyisi e tijegi. To kata kamano, ka inyalo ti kodgi e nonro ma biro mbele, wageno ni mae biro konyowa mondo wapuonjre kaka imedo bero geng’o, thiedho kata tieko chandruok mag thieth ma kuom nyithindo man kodSCA.

Ibiro tem matek mondo orit weche ma korka kori to ma ok nyal kony.Weche ma inyalo yud kuomi ka owuok kuom nonro ma ibiri timo ndalo ma biro ok bi ket e andikemari mar thieth.

TO KA AYIE MAR CHIWO RANYISI SANI, BE ANYALO KWAYO MONDO RANYISI MA ACHIWO OKETHI BANG’E?

Ka ing’ado bang’e ni diher mondo mi ranyisi magi okethi, nyaka ikon jathieth ma Njuguna kata jok ma ochung’ne e kanyakla mar nonro niigolo yie mane iyie godo mondo ranyisi magi okan kendo oti godo e research mabiro mbele.

Seche ma nonro maduong’ dhi nyime, ikwayi ni itudri gi ja ta nonro Mr.Joseph Kipkoech e +XXXXXXXXX. Bang’ nonro maduong’, ikwai ni itudri gi jo bura matimo nonro mar kar puonjruok mar Moi e +2540787723677. Ka weche weche ma tudo number mar ranyisi mari kod in ose kethi, ranyisi magi ok nyal chak tud kodi kendo ok wabinyalo yudo gi mondo oketh gi. Kendo, kata obedo ni ranyisi magi ibiro kethi:

Ok wanyal yudo kendo ranyisi kata weche ma wasechiwo ne jotim nonro.

Okinyal golo ranyisi mari ka osetimne nonro matut kata ka osepime.

Yieigo sei e kar go sei achiel piny ka iyie mondi rembi ma itiyogo ka ranyisi okan ne nonro manyalo biro mbele, kata ka ok iyie mondo rembi ma itiyogo kaka ranyisi mondo okan.

Ayie mondo okaw remb nyathina mar ranyisi okan ne nonro manyalo biro mbele mar SCA, malaria, kod touché moko ma mako nyithindo ma nigi SCA.

______________________________ __________________________

Sei mar ja nyuol Tarik

____________________________________ ________

Sei mar janeno (ka dwarore) Tarik

OK AYIE mondo **okaw remb nyathina** mar ranyisi okan ne nonro manyalo biro mbele mar SCA, malaria, kod touché mamoko mag nyithindo man kod SCA.

______________________________ __________________________

Sei mar jatuo Tarik

## APPENDIX K: INFORMED CONSENT (DHOLUO TO ENGLISH)

LETTER FOR JOINING STUDY

Study heading: Strengthening Plans for Malaria Prevention in Children with a Blood Disease in East Africa

**Researcher**: Doctor Festus Njuguna

You are requested to allow your child to be in study because your child has a blood disease

WHAT ARE THE THINGS YOU NEED TO KNOW IN STUDY?

You are requested to allow your child be in study. Before you accept to allow your child to be in this study for treatment, it is good you know that this like studies requires only those who have decided to be part of it. Your child’s being in the study voluntary. If you accept to read from this paper properly then take your time to make up your mind. Your child’s Dr. for the study or whoever is doing the study on his behalf/ for him will be speaking regarding this paper together with you. If you agree ask any question that you don’t understand properly

This paper gives us detailed information of what will take place in this study. This paper tells us in details over injuries, difficulties, discomfort, and other difficult issues. It is important that you ask question and you clearly understand what is required in this study. We encourage you to talk with your family or your friends before you allow your child in this study. You can take one of these papers at home while you are still thinking as to whether your child should join the study. If you accept to allow your child to join this study then sign on this paper. You will be given a paper containing this issues that is signed and dated for you. If she/he accepts tell study doctor or a researcher if your child is in another study.

WHO WILL BE MY STUDY DOCTOR?

If you accept to be in the study Doctor Njuguna will be the study doctor and will be talking with or your regular health worker during the period you will be in the study and later if you need. Donation from learning institution of United States National Institute of Health (NIH) will support this study. Doctor Njuguna and his people doing the study will be paid from this donation.

WHY IS THIS STUDY DONE?

You are requested to allow your child to join this study because she/he is between the age of 1 and 10 years, she/he has a blood disease and lives in an area that has high prevalence of malaria.

We would wish to get better ways of preventing malaria in children with blood disease. This study will enable us know better treatment that can be used to prevent malaria in children with blood disease in areas of Africa with malaria. In this study we will compare among the three malaria drugs available in chemist so that we can find out which treatment if any that is better in reducing number of days of your child having malaria. Study treatment available in this study are as below. Your child will be examined, your child will receive one of the three treatments per oral as a way of preventing malaria:

- Proguanil (also known as Paludrine) it is the current highest standard in prevention of malaria in children with blood disease in Kenya. Sometimes your child has been on this drug so as to prevent malaria. This drug is taken daily.

OR

- Sulfadoxine/Pyrimethamine-Amodiaquine (SP-AQ) is a combination of drug containing sulfadoxine and pyrimethamine taken together with amodiaquine. This drug is taken monthly

OR

- Dihydroartemisinin-Piperaquine (DP) it’s a combination of dihydroartemisinin and piperaquine. This drug is taken monthly.

I would wish to add that, if your child gets malaria, s/he will be treated through the mouth by Artemether-Lumefantrine (AL). All children below the age of 5 will receive penicillin everyday through the mouth this will prevent spread of bacteria which is highest in children with blood disease.

HOW MANY CHILDREN WILL BE IN THIS STUDY?

About 246 will complete this study. This study will be done at Homabay county hospital in Homabay, Kenya.

WHAT IS REQUIRED IN THIS STUDY?

If you allow your child to be in this study you will be requested to sign this paper. Your child will go through the following steps to show that she/he qualifies for enrolment

Examination at visit

In case you get in to the study, researcher has to know if your child qualifies. This helps in enrollment step.

In the examination visit you will go through these steps and documentation of the findings:

- We shall examine your health
- We shall again check treatment that your child receives.
- We will perform electrocardiogram this measure heart bit over the body using a machine
- We will collect about 1Ml of your child’s blood so that we test some the protein that causes blood disease. If either you or your child has had blood made in the marrow that collects gases and removes unwanted gases because results for sickle cell test, some of the blood made in the marrow that collects and removes unwanted gases you can give us documents for the result, this test shall not be done in examination visit
- We shall collect about 1Ml your child’s blood so that we test part of the blood made in the bone marrow that collects gases and removes unwanted gases because of the protein that causes blood disease and will perform routine blood test to determine if it is safe to be enrolled into the study. Here your child needs to complete test, that shows that your child is not prescribed for test that you know, However after that he/she can be enrolled in the study when she comes is when we can now if she/he qualifies.

Your first entry

During your first registration, the following procedure will be followed, then recorded

- We shall document results for your child how it has been in the hospital, and result that has been since the first coming.
- We shall document the drugs that your has used
- We shall do body examination together with height and weight.
- We shall collect about 5ml of blood
- We will collect the blood less than ½ ml from a finger prick of your child.
- If necessary, we will administer to your child a Meningococcal vaccine.  This vaccine will help protect your child against bacteria that causes meningitis. This may not be necessary if your child has already received this vaccine, which is routinely recommended in Kenya.
- We shall give you penicillin for 30 days ( if your child is under the age of 5 years), this is a treatment plan to prevent bacterial infection for children with blood disease
- We shall register your child in Kenyan National Insurance Fund (NHIF)
- We shall show how to protect and register your child

Also with this visit plan, we shall know which malaria study your child will get. For that to happen, your child will be given (picking numbers) so that they get daily Proguani, SP-AQ monthly, or DP monthly. Your child has one in three chance of getting drug that is in plan. Any drug that a child should get in the study will be to protect against malaria in that study. All children will get some kind of malaria prevention drug..

Subsequent visit (monthly visit)

In the monthly visit, these are what will be followed:

- We shall study how your child is doing hospital wise, together with his/her treatment issues as per her/his last visit.
- We shall ask questions, together with weight and height.
- We shall collect less than 1/2ml of blood after pricking his/her finger to test for sickle cell anaemia and malaria parasites. If your child has a headache, or has had a headache that has lasted less than 24 hours, he/she will have a malaria test immediately; if your child has had no fever, his/ her blood will be stored for test when the study is over.
- After every three months, we shall take about 2 ½ ml of blood for test.
- When it reaches sixth month of visit and twelfth month we shall collect some blood of about 1ml to test blood disease.
- If you sign a different form to consent for blood collection for visits after 3,6,9 an 12 months will collect 2 ½ of blood for future research studies.
- If we have to, we shall do ECG.
- We shall give you study treatment and how it is used
- For 30 days we shall give you daily penicillin (if he/she is less than 5 years)
- About 20 children will be selected to go back to clinic after two days for a repeat ECG this is done every month during study. This will be done so as to get results for ECG for study during the whole time. This will be for children who are selected for the DP of the study
- If your child is found to be having malaria after, will be treated at that time with AL.

Scheduled Visits (as required)

According to the study, if your child who is at home has fever, it could be because they are feeling hot, or body hotness that is over 37.5, we request you call the study leader to organize how you can be assisted with the people doing the study. In this visit, the following will be followed:

- We shall investigate your child health status, alongside other treatment issues in regards to the last visit.
- We shall enquire, together with weight and height
- We shall collect not more than ½ mL of blood after pricking his/her finger. If your child has fever or has had fever that has not lasted 24 hours, he/she will be tested for malaria at that particular time; if your child has not had fever, his/her blood will be stored to be tested at the end of the study.

If your child’s blood is found to be having malaria he/she will be treated immediately with AL

How long will your child be in the study?

He/She should be taking about 12 months, but it’s done monthly. Your child’s study will be completed after the 12^th^ month after visits

You can choose to avoid being in the study any time and there is no particular time that will be decided. For that, you will not receive treatment or study benefit, and your child will be taken to a doctor who is not in the study such as peadiatrician, hospital or clinic. And even if you have chosen not to be in the study, we will encourage you talk with your doctor before

What is it harm of the study?

According to how you have been in this study team, your child can have certain things that may occur later, if you decide that you can join, you need to discuss these issues with doctor and those who always help you

It is possible that every drug to have something that follows. You should know some of the things that follows which may come in the study before you allow your child to join the study. Things like injuries, feel bad, drugs that do not agree with the body, and other things that may come that are not known.These injuries can be as a result of increased complication associated with the sicke cell anaemia, if the complications are very serious they can be fatal.

Proguanil may cause this, some of this, all of this, may not cause, some of the things that may come that are written here.

Like these:

Nausea Anemia

- Vomiting
- Abdominal pain
- Bad dreams Cough
- Head ache Restlessness
- Diarrhea Rash
- Weakness Fever
- Loss of appetite Dizziness
- Itchiness
- These are less likely
- Anxiety
- Fast, strong, or abnormal heart beat
- Mouth wounds
- Forgetfulness
- Excessive rash

SP-AQ may cause, all or some or not one of the following problems.

That which is likely to happen:

- Headache Cough
- Vomiting Nausea
- Loss of appetite Abdominal pain
- Diarrhea Weakness
- Fever Tiredness
- Sleep problems Rash
- Sleep sickness Itchiness

These are not common

- Eye problems
- Liver diseases
- Anaemia

DP may cause, all or some or not one of the listed problems below.

Most common:

- Head ache
- Fever
- Cough
- Boils
- Diarrhoea

The most less likely:

- Dizziness
- Vomiting
- Nausea
- Loss of appetite

Some Injuries:

Such as collection of blood from your child, discomfort. Boil, bleeding in excess, blood clotting, falling. For reducing chances of injuries, we only employ those who are trained and proper equipment. In addition to that, your child can get some injuries and some undesired effect due to the study drugs which can increase serious complications of the sickle cell disease and can be fatal. Its better you share any problems with the researchers

ISTHERE BENEFIT AS A RESEACHER?

If you allow your child in this study. Your child can get treatment benefit. Children who join the study will get free malaria prevention drug and for other disease causing organism, free thorough monthly assessment, free malaria test if they have fever, and put on Kenyan National Hospital Insurance Fund. This assistance has the benefit of reducing problems related to treatment emanating from malaria and SCA

For a long period, we hope that in coming days what has been learnt from this study will help children from Africa with sickle SCA by getting better treatment for malaria prevention

WHAT ARE SOME OTHER WAYS AVAILABLE HERE FOR BEING IN THIS STUDY?

It is not a must your child be in this study. Even if you don’t allow your child to be in it, he/she can still get treatment from to time to time from regular health providers.

WILL INFORMATION OF MY CHILD BE KEPT CONFIDENTIAL?

Your child will not be identified by her/his name or by reports sent to people outside Homa bay county hospital. For any writings leaving Homa bay county hospital your child will be given a number that is secret, document containing the secret numbers together with the names of the children and other information that talks about her/him are kept secret and be kept by the team that does research.

All will be done to ensure that all that’s written about your child are kept permanently confidential. However there is a possibility of your child’s information being known. Some offices and other people who are not part of the study may be able to see your child’s information about treatment and study *information*

Among them are people from:

- Researchers from Moi and Duke university and research institute of Duke;
- Research institution of Moi and Ethics committee and the Duke University of Health System institutional Review Board
- The overseers for NIH from the American

The documentation for the study shall be kept for 10 years after the study in accordance with the regulations, research information that are in the treatment documents shall be kept for ever

During the study period your health worker and researchers may go through research matter together with other people. This may consist of:

- Those who check the research, those checking about honesty of the study
- Those who have information showing beginning of treatment, those who tell problems drugs brings to sickness of a child and also.
- The people who compile all research matters in writings.

When your child is in this study, information collected from him can be documented in his/her medical records.

You have a right to decline to be in this study any time. So as to stop, it is better you tell your health care provider for the study that you would like to stop. If you want your child to join this study, you must sign to show that you allow the research team to examine your child in relations to her/his treatment. If you refuse to allow us examine your child’s health in details then your child shall not be in the study. However, declining to sign in this form will not hinder you or your child in getting medical help.

Sometimes the result for study can be taken to a board that does research or be written in research papers, anything about your child will not be shared and your child will not be identified by name. Information about treatment shall be found at <http://www.ClinicalTrial.gov> and pan African clinical trials registry (<http://www.pactr.org>). This website shall not contain information that will reveal your identity. Most of the time this website will carry results in summery. You can visit this website anytime.

Information about your child shall not be used in anyway or be shown anybody without your permission.

WHAT IS THE COST?

There is no additional charge that shall be added to your child for being in this study. Any study, drug for the study or tests will be offered to your child free of charge. In addition to that, if your child is put in the study we shall put her/him in KNHIF. All sought of treatment can be paid by NHIF

HOW ABOUT COMPENSATION?

There is no money that will be available or compensation for child’s participation in the study. However you shall get five hundred Kenya shilling that will assist you in transport to access these treatment programs. Sometimes this may not cater for full transport but this will facilitate being in the study.

WHAT IF MY CHILD GETS INJURED?

Any treatment that may be required at that time will be found at Homa Bay county Hospital. If your child gets injured as a result of being in the study. There is no agreement with Moi University, university of Duke from the medical research of Duke, Homa Bay County or NHIF to offer any compensation for injuries related to this research. There is no agreement with the University of Moi, University of Duke through medical school of medical research, Homa bay county hospital or NHIF to offer any compensation or compensation for any injury related to this study, money or free treatment.

For question about the study or injuries in connection with the study get in touch with Dr. Njuguna at +XXXXXXXXX during working hours and on +XXXXXXXXX after working hours, weekend and holidays.

WHAT ABOUT MY RIGHT TO REFUSE TO ALLOW MY CHILD BE IN OR LEAVE THE STUDY?

You can decide to refuse to allow your child be in the study. You can also decide to allow your child to be in the study or leave the study anytime. If you decide to stop your child from being in the study there are no new information that will be collected from your child unless he/she has drug related problems, if such problems occur we may want to check again all his/her treatment records. All research information will be sent to the research lead which is American NIH.

Your decision to refuse to allow your child be in or leave the study will not attract any punishment, however, you will not receive any treatment or profit in the study, and care of your child will be transferred to health care providers who are not in the study such as pediatrician major or small health facilities. This will not prevent you or your child from receiving from the regular doctors from time to time.

FORCEFUL REMOVAL OF A PERSON FROM THE STUDY.

Study health care provider may decide to remove your child from the study when:

- Life of your child is in danger or
- Your child develops problems from some of the things used. Or
- Treatment requirement is not offered in the study or
- When study health care providers decide that it is not the best thing for the child to continue.

NIH, Dr. Njuguna or other study health care providers, or law implementers may decide to stop this study anytime without you knowing. If this happens you will be told and your study health care provider will talk to you about other ways

WHAT IF THERE IS NEW INFORMATION?

You will be informed of any new information that people have learnt during the study that may affect you or that may require your child to continue being in the study.

WHOM CAN I CALL IF I HAVE QUESTION OR PROBLEM?

For questions about the study or injuries in connection with the study or you have a problem, any issues get in touch with Dr. Njuguna on +XXXXXXXXX during working hours, after the working hours, weekends and holidays. For questions about your child’s rights as a researcher, or talking about problems, any issues you have, or ideas related to the study, or getting information or talking about problems, desire, or idea in connection with the study or getting other information, or supporting the study, get in touch with study board over approved behavior of Moi at +2540787723677. Those who approve health matter at Duke University has also studies this study, if you have any questions for the members that may assist forward them to +1(919) 668-5111.

ABOUT CONSENT

Requirements for this study, steps to be followed, injuries, has been explained to me and my child. Am allowed to ask questions, and my questions have been answered satisfactorily. I have been told whom to call when I have questions, talks about problems, desires, or ideas in connection with the study. I have read about being allowed and allowing my child to be in the study and understanding that I can stop my child from the study anytime. I have been told that I will be given a copy to keep which is signed and dated.

___________________________________

Printed name of the parent or legal guardian.

____________________________________

Signature of the parent or legal guardian. Date Time: ___: ____

____________________________________

Signature of Witness (if applicable) Date Time: ___: ____

I have explained properly about the study that is discussed in this paper. I have answered questions asked by the parent/ guardian and I will answer any questions to the best of my ability. I will tell family about any changes in ways or benefits from the study that may affect the desire of the parent to allow his/her child to be in the study.

____________________________________ ___________

STUDY STAFF OBTAINING CONSENT Date

_________________________________ __________

SIGNATURE OF STUDY STAFF OBTAINING CONSENT Date

WHAT WILL THE BLOOD SAMPLE BE USED FOR?

During the study, after lasting more than 12 months blood will be collected from the child in the volumes shown above ( if you join or come after 12 months) we will collect blood to do regular tests to observe results for SCA, a disease that is caused by mosquitoes and treatment.

In addition to that, blood sample will be collected and be used in the study in future if you accept this request. These samples will be stored for a long time in one of the universities or research organizations doing this research, this includes Moi University or Duke University. These samples may be shown to researchers in other learning institutions. Anything that is done with the stored samples must be approved by relevant people who oversee studies or committee that regulates behavior.

These are some of the most important things that you need to know the about the study:

- We will collect about two and a half romomilliliter of blood to be stored for future study at registration during visits which we will collect blood for routine care. At enrollment this sample will be used in malaria research, SCA or other diseases in children with sickle cell
- We shall store the remaining sample in approved paper from blood that we collected from you this will be used to test malaria parasites,
- Information we get from this study will not affect your child’s care.
- These samples will not be sold or be used for creating wealth or profit
- We can do study human or malaria parasites present in the sample and there is nothing that will be documented about your child’s care and these samples will be labeled by secret number so that someone does not identify your child.
- There is no direct benefit if you allow us use him/her sample in this work. However, they can be used for future study, we hope that this will help us to learn how to better prevent, treat, or complete problems of treatment of children with SCA.

Great effort shall be done to protect your information but this cannot be guaranteed. Information that will be received from you from the study shall not be recorded in your treatment notes.

IF I DECIDE TO GIVE SAMPLE NOW, CAN I REQUEST THAT THE SAMPLE BE DESTROYED LATER?

If you later decide later that you would like your samples be destroyed, you must tell Dr. Njuguna or representatives for the study that you have withdrawn consent for your sample to be stored for future research.

During the main study you are requested to get in touch with study Mr. Joseph Kipkoech at +XXXXXXXXX.

After the main study you are asked to get in touch with study committee members from Moi University at +2540787723677.

If the numbers that link your sample and you have been destroyed, then your sample cannot be linked to you and we will not be able to get them for destruction.

Even though your samples will be destroyed:

- We will not be able to get back your samples or information that has been delivered to researchers.
- You cannot get your samples after your sample has been studies in details or tested.

Kindly sign down here in the space provided if you allow your sample to be stored for future use, or if you refuse to allow your blood sample to be stored

I accept my sample that my child’s blood sample be stored for possible future research for SCA, malaria, and other diseases with SCA

______________________________________________________________________

Signature of Parent Date

______________________________________________________________________

Signature of Witness (if applicable) Date

I DECLINE to allow my child’s blood sample be stored for future study about SCA, malaria, and other diseases in children with SCA.

______________________________________________________________________

Parent ‘s Sign Date

## APPENDIX L: MEDICATIONS THAT PROLONG THE QT INTERVAL

The following medications are known to prolong the QT interval and are likely to be used in children:

| Azole antifungals | Fluconazole |
| --- | --- |
|  | Itraconazole |
|  | Ketoconazole |
|  | Posaconazole |
|  | Voriconazole |
| Fluoroquinolone antibiotics | Ciprofloxacin |
|  | Gatifloxacin |
|  | Levofloxacin |
|  | Moxifloxacin |
|  | Ofloxacin |
|  | Sparfloxacin |
| HIV antiretrovirals | Efavirenz |
|  | Lopinavir |
|  | Saquinavir |
| Macrolide antibiotics | Azithromycin |
|  | Clarithromycin |
|  | Roxithromycin |
|  | Telithromycin |
| Other antimicrobials | Metronidazole |
|  | Telavancin |
|  | Pentamidine |
|  | Foscarnet |
| Psychotropics | Haloperidol |
|  | Tricyclic antidepressants |
|  | Fluoxetine |
|  | Escitalopram |
|  | Citalopram |
|  | Risperidone |
| Bronchodilators | Albuterol |
|  | Levalbuterol |
|  | Formoterol |
|  | Salmeterol |
|  | Terbutaline |
