# Supplementary material for: Monthly sulfadoxine/pyrimethamine-amodiaquine or dihydroartemisinin-piperaquine as malaria chemoprevention in young Kenyan children with sickle cell anemia: A randomized controlled trial
Source: PLoS Med. 2022 Oct 10;19(10):e1004104. doi: 10.1371/journal.pmed.1004104 (PMC9591057; doi:10.1371/journal.pmed.1004104)
Supplement: S1 Analysis — (DOCX) [file pmed.1004104.s003.docx]

**Statistical Analysis Plan**

**Protocol Identifying Number: 1R01HL134211**

**Version:** 1.0

**Date:** 5/25/2021

**Author:** Angie Wu

**Enhancing Preventive Therapy of Malaria in Children with Sickle Cell Anemia in East Africa (EPiTOMISE)**

# Amendment history

**Date Brief description of change**

8/7/18 v1.0 created.

6/28/19 **§** 2.1.3 added, defining As-Treated population. Added **§**6.2 Stopping Rules. Removed **§**6.6 Drug Exposure and replaced with **§**6.7 Concomitant Medications. Updated abbreviations to reflect usage in SAP.

8/15/19 Added stopping rule and concomitant medications. Changed most endpoints besides all-cause mortality to binary instead of time to event. Updated to reflect analytic approach for all endpoints in final analysis.

3/4/20 Edited details of data collection and analysis population to be consistent with Protocol v8.0. Updated analysis methods for all outcomes.

12/14/20 Removed references to analysis on drug resistance markers. Clarified exploratory analyses.

03/08/21 Modify after investigator’s review.

05/04/21 Final review.

05/25/21 v1.0 modified after signature to correct description of ATP analysis population and clearly specify when analysis populations would be used for analysis.

**Table of Contents**

[Amendment history 1](#_Toc70947063)

[List of Abbreviations 4](#_Toc70947064)

[1. Study details 6](#_Toc70947065)

[1.1 Introduction 6](#_Toc70947066)

[1.2 Study Objectives 6](#_Toc70947067)

[1.2.1 Primary Objective 6](#_Toc70947068)

[1.2.2 Primary Objective 6](#_Toc70947068)

[1.3 Study Design 6](#_Toc70947069)

[1.3.1 Primary Objective 6](#_Toc70947068)

1.3.2 Protocol Change 7

1.3.3 COVID-19 Impact 7

[1.4 Sample Size and Power 7](#_Toc70947070)

[1.5 Timing of Major Assessments 7](#_Toc70947071)

[2. Analysis Sets 8](#_Toc70947072)

[2.1 Definition of Analysis Sets 8](#_Toc70947073)

[2.1.1 Intention-to-Treat (ITT) Population 8](#_Toc70947074)

[2.1.2 As-Treated (AT) Population 8](#_Toc70947075)

[2.1.3 According-to-Protocol (ATP) Population 8](#_Toc70947076)

[3. Background Characteristics 9](#_Toc70947077)

[3.1 Disposition of Patients 9](#_Toc70947078)

[3.2 Demographic and Baseline Characteristics 9](#_Toc70947079)

[4. Outcomes 10](#_Toc70947080)

[4.1 Primary Outcome 10](#_Toc70947081)

[4.2 Secondary Outcomes 10](#_Toc70947082)

[5. Analysis Methods 13](#_Toc70947083)

[5.1 General Principles 13](#_Toc70947084)

[5.2 Censoring 14](#_Toc70947085)

[5.3 Handling of Missing Data 14](#_Toc70947086)

[5.4 Statistical Hypotheses 14](#_Toc70947087)

[5.5 Analysis of the Primary Outcome 14](#_Toc70947090)

[5.6 Analysis of the Secondary Outcomes 15](#_Toc70947091)

[5.7 Sub-Group Analyses 16](#_Toc70947092)

[6 Safety and Tolerability Analysis 16](#_Toc70947093)

[6.1 Serious Adverse Events (SAEs) 16](#_Toc70947094)

[6.2 Stopping Rules 16](#_Toc70947095)

[6.3 Laboratory Data 17](#_Toc70947096)

[6.4 ECG Data 17](#_Toc70947097)

[6.5 Baseline Medications and Interventions 18](#_Toc70947098)

[6.6 Vitals and Physical Assessments 18](#_Toc70947099)

[7 DSMB and Interim Analyses 18](#_Toc70947100)

[7.1 Interim Analyses 18](#_Toc70947101)

# List of Abbreviations

**Abbreviation or Explanation
special term**

ACS Acute chest syndrome

ALT Alanine aminotransferase

ATP According-to-protocol

CBC Complete blood count

CI Confidence interval

DCRI Duke Clinical Research Institute

DP Dihydroartemisinin-piperaquine

DSMB Data and Safety Monitoring Board

ECG Electrocardiogram

EPiTOMISE Enhancing Preventive Therapy of Malaria In children with Sickle cell anemia in East Africa

GCP Good clinical practice

H_O_ Null hypothesis

H_A_ Alternative hypothesis

HBCH Homa Bay County Hospital

HbF Hemoglobin F

HbSS Hemoglobin SS

HPLC High-performance liquid chromatography

IR Incidence rate

ITN Insecticide-treated bednet

ITT Intention-to-treat

LM Light microscopy

MOP Manual of procedures

MSEC Milliseconds

PCR Polymerase chain reaction

QTcF Fridericia’s Corrected QT Interval

RDT Rapid diagnostic test

SAE Serious adverse event

SAP Statistical analysis plan

SCA Sickle cell anemia

SD Standard deviation

SNP Single nucleotide polymorphism

SOC Standard of care

SOP Standard operating procedure

SP-AQ Sulfadoxine/pyrimethamine-amodiaquine

SUSAR Suspected, unexpected serious adverse reaction

WHO World Health Organization

# 1. Study details

## 1.1 Introduction

This Statistical Analysis Plan (SAP) contains descriptions of study endpoints, definitions of analysis populations, variable descriptions and details for analyses and summaries of study data. Specifications of tables, figures, and data listings will be provided in the Appendix. The reader of this SAP is encouraged to read the clinical protocol for additional detail on conduct of the study, operational aspects of clinical assessments, and timings of individual patient assessments. A Data Safety Monitoring Board (DSMB) will monitor this study based on the DSMB charter.

## 1.2 Study Objectives

### 1.2.1 Primary Objective

The primary objective of the study is to evaluate the effect of daily Proguanil (current standard of care [SOC]), monthly Sulfadoxine/Pyrimethamine-Amodiaquine (SP-AQ), or monthly Dihydroartemisinin-Piperaquine (DP) to prevent *P. falciparum* malaria in children with SCA.

1.2.2 Secondary Objectives

The main secondary objective is to evaluate the effect of monthly SP-AQ or monthly DP relative to SOC in preventing painful events in children with SCA. This will be considered the m*ain hematologic endpoint.*

Other secondary objectives of the study are to evaluate the effect of monthly SP-AQ or monthly DP relative to SOC on additional parasitologic, hematologic, and general morbidity outcomes as described in Section 4.2.

## 1.3 Study Design

EPiTOMISE is a three-arm, single-site, open-label randomized clinical trial in children with SCA of malaria chemoprevention comparing daily Proguanil (current SOC), monthly SP-AQ, or monthly DP.

Up to 246 pediatric patients ranging from age 1-12 years satisfying enrollment criteria will be recruited into the trial over 3-4 years and randomly allocated to one of the three treatment arms.

Patients will be enrolled at one site, Homa Bay County Hospital (HBCH). Trial follow-up over 12 months will consist of routine and as-needed study visits, intermittent laboratory evaluations, and phone calls during the treatment period.

For additional details on the study design, please refer to the protocol sections **Protocol Summary and Schematic of Study Design.**

1.3.1. Study Implementation

Enrollment began January 18, 2018. On October 31, 2018, owing to several participant deaths among the children allocated to receive SP-AQ, enrollment of new patients was paused and children allocated to SP-AQ were temporarily administered daily Proguanil (SOC). Starting April 1, 2019, the DSMB and ethical boards allowed:

1. Re-enrollment of new participants.
2. Dispensing of SP-AQ to all patients who were allocated to SP-AQ.

*The change in treatment administration (crossover) will be captured within all statistical models evaluating treatment assignment for event rates using the As-Treated (AT) analysis population*.

1.3.2. Protocol Change

The major changes to the protocol implemented April 3, 2019 were:

1. Exclusion of children with low hemoglobin;
2. Collection of baseline laboratory testing at screening (compared to enrollment); and
3. Enhanced monthly hemoglobin monitoring, SAE review, and patient referral.

*The change in protocol will be evaluated by including an interaction term (pre/post-protocol change) with treatment in the models for the primary and main secondary endpoint analyzing incidence rate ratios (IRR)*. *This will be a sub-group analysis using the AT population.*

1.3.3. COVID-19 Impact

Owing to the COVID-19 pandemic, on April 1, 2020 operational changes were implemented to decrease participant travel, reduce clinic participant and staff density, and augment retention on protocol. As a result, monthly interval visits were conducted by telephone every other month, and scheduled laboratory testing was deferred until the following in-person interval visit.

*The impact of COVID-19 will be evaluated by including an interaction term (pre/post-COVID-19) with treatment in the models for the primary and main secondary endpoints analyzing incidence rate ratios (IRR)*. *This will be a sub-group analysis using the AT population.*

## 1.4 Sample Size and Power

This study was primarily powered for the primary outcome of clinical malaria. Assuming 65 patients would be enrolled within each of the three treatment arms, the sample size would allow up to 20% of the subjects to be lost to follow-up and still maintain >90% power to detect a reduction of 40% in the DP arm, ~40% power to detect a reduction of 20% in the Proguanil arm, and >90% power to detect a reduction of 40% in either arm. We assumed that each experimental arm would be made at the 0.0269 level in order to preserve a nominal alpha level of 0.05.

Using the sample size specified for the primary endpoint, we estimated that we would have at least 80% power to detect a 35% reduction in painful events, the main secondary endpoint.

For a detailed description of the power calculations used in this study, please refer to protocol **§9.5 Sample Size.**

## 1.5 Timing of Major Assessments

At screening, patients will sign an informed consent; complete an ECG; assess CBC, creatinine, and ALT; and complete hemoglobin electrophoresis or HPLC.

At enrollment, patients will provide their medical and social history, complete a review of systems and physical examination, and undergo randomization. Following randomization, study medication will be dispensed.

At monthly interval visits, patients will be assessed using interval history, review of systems, physical exam, and blood collection. In addition, up to 20 children allocated to DP will be enrolled in a sub-study for which they will have repeat ECG testing following the third dose of each monthly DP course. In all participants, these monthly interval visit assessments will serve as the source for:

- study medication adherence,
- the presence of SAEs/SUSARs,
- malaria RDT testing (in those with fever or a history of fever)
- regular clinical laboratory testing
  - hemoglobin concentrations,
  - CBC/creatinine/ALT (every 3 months) and
  - hemoglobin electrophoresis (every 6 months).
- regular research laboratory testing
  - *P. falciparum* parasitization
  - *P. falciparum* drug-resistance polymorphisms

To ensure the proper timing of routine assessments, study participants will be scheduled to come to the study clinic 30 (+/- 5) days following the preceding follow-up visit. If a study participant is not seen in the study clinic within this time, study staff will contact by phone the participant’s parent/guardian and if necessary visit the home and ask the patient’s parent/guardian to come to the clinic.

# 2. Analysis Sets

## 2.1 Definition of Analysis Sets

We will define three populations, including an as-treated (AT) population of all treated participants according to the treatment administered, an intention-to-treat (ITT) population of all randomized patients irrespective of confirmed receipt or administration of study drugs, and an according-to-protocol (ATP) population, consisting of those who were randomized and treated per protocol. We will assume that subjects still enrolled will have received study drugs at routine follow-up visits.

### 2.1.1 Intention-to-Treat (ITT) Population

The ITT population will consist of all randomized patients irrespective of their confirmed receipt or administration of study drugs. For all analyses using the ITT population, patients will be analyzed in the treatment group to which they were randomized. The ITT population will be used for descriptive statistics in the DSMB interim and final reports. The ITT population will also be used to evaluate both dichotomous and event rate data for the primary and secondary endpoints.

### 2.1.2 As-Treated (AT) Population

The As-Treated Population is defined as the population of all participants, with treatment assignment reflective of the actual treatment the patients received during the month under observation. The rationale for the definition of an AT population is that, from October 2018 until March 2019, children allocated to SP-AQ were temporarily crossed-over to the Proguanil (SOC) arm; those who were originally randomized to SP-AQ who remained active in April 2019 were then reverted back to monthly SP-AQ (see Section 1.3). We will account for the crossover that occurred during the study pause in which patients in the SP-AQ arm were treated with standard of care, Proguanil. *The AT population will be the primary population of interest in the final analysis. This population will be used for all primary and secondary models analyzing incidence rate ratios (IRR).*

### 2.1.3 According-to-Protocol (ATP) Population

The According-to-Protocol Population is defined as the population of all participants, with treatment assignment reflective of receiving the actual treatment assigned per protocol (ATP). We will assume that subjects still enrolled will have received study drugs at routine follow-up visits. *The ATP population will be used to evaluate both dichotomous and event rate data for the primary and secondary outcomes.*

# 3. Background Characteristics

## 3.1 Disposition of Patients

The number of patients in the ITT population will be summarized by treatment group. The number and percentage of patients who completed and patients who withdrew from the study will be presented for each treatment group and overall. The reasons for discontinuation from the study will be reported by number and percentage in each treatment group and overall.

Subject accrual will be tabulated on a monthly basis by treatment arm. Data availability, as measured by visit initiation, will be reported at baseline and each interval visit by treatment arm.

## 3.2 Demographic and Baseline Characteristics

Demographic, baseline clinical and anthropometric characteristics, as well as medial history will be tabulated and summarized as descriptive statistics by treatment for the ITT population. The baseline characteristics that will be reported may include, but are not restricted to:

- Age (years)
- Sex
- Tribe/ethnic group
- Weight (kg)
- Height (cm)
- Mid upper arm circumference in patients under 5 years of age
- Malnutrition
- Diabetes
- Regular medical care for SCA
- Baseline use of proguanil
- Baseline use of penicillin prophylaxis in patients under 5 years of age
- Baseline use of hydroxyurea
- White blood cell count (10^9^/L)
- Neutrophil count (10^9^/L)
- Hemoglobin (g/dL)
- Platelet count (10^9^/L)
- Mean corpuscular volume (fl)
- HbF (%) at screening, both in overall population and subset only to patients taking hydroxyurea
- HbS (%) at screening
- ALT (units/L)
- Creatinine (umol/L)
- RDT positive
- QTcF interval (msec)
- Nights under ITN within past week
- House treated with residual spraying within 6 months
- Receipt of blood transfusions within 12 months
- Treatment for malaria within 12 months
- All-cause hospitalizations within 12 months
- Pain crises within 12 months
- Dactylitis episodes within 12 months

Unless specified otherwise, a baseline value is the last assessment taken prior to or at randomization. When there is a missing baseline assessment it will not be imputed. Thus, patients are excluded from any changes from baseline analysis for which they have a missing baseline value.

# 4. Outcomes

## 4.1 Primary Outcome

The primary efficacy hypotheses for this study are:

- Monthly SP-AQ is superior to daily Proguanil for the prevention of clinical malaria in children with SCA.
- Monthly DP is superior to daily Proguanil for the prevention of clinical malaria in children with SCA, with a protective efficacy twice that of monthly SP-AQ.

The primary outcome, clinical malaria, will be expressed as both the cumulative incidence at 12 months and as an incidence rate (IR; the number of episodes per patient-year at risk of malaria) with 95% confidence interval (CI) and compared for effectiveness. Clinical malaria will be defined as having either:

- Uncomplicated malaria:
  - Presence of *P. falciparum* parasitemia of any density AND
  - Temperature of ≥ 37.5**°**C or history of objective or subjective fever in the preceding 24 hours

OR

- Case meeting the definition of severe malaria according to WHO criteria (see below for definition)

Uncomplicated malaria events will be captured from acute care and monthly interval visit data, and be either directly observed in the study clinic or recorded from review of outside records. Events either observed or collected outside the study will be required to satisfy the above case definition. Only confirmed RDT-positive events will be recorded as primary endpoints; suspected malaria cases that are not confirmed by RDT will be recorded as secondary endpoints.

Severe malaria will be defined below in **§4.2 Secondary Outcomes**.

## 4.2 Secondary Outcomes

The main secondary outcome, pain crisis, will be expressed as both the cumulative incidence at 12 months and as an incidence rate (IR; the number of episodes per patient-year at risk of malaria) with 95% confidence interval (CI) and compared for effectiveness. *This endpoint represents the main hematologic outcome.*

A pain crisis will be defined as pain lasting 2 hours or more without obvious cause (either as inpatients or outpatients). Pain crisis will be identified through interval visit data. At each visit, the patient is asked for the number of pain crises experienced since the previous visit.

Other secondary outcomes *based on the number of events* will be expressed as both the cumulative incidence at 12 months and as an incidence rate (IR; the number of episodes per patient-year at risk of malaria) with 95% confidence interval (CI). The IR will be evaluated through the 12-month follow-up visit and compared for effectiveness. Secondary outcomes based on the dichotomous incidence (yes/no), will be expressed as the cumulative incidence at 12 months and compared for effectiveness using Fine & Gray’s test.

All-cause mortality will be expressed as the mortality percentage at 12 months with 95% confidence interval (CI) based on Kaplan-Meier methods to allow for censoring for patients who withdraw or are loss to follow-up. The Kaplan-Meier estimate at 12-months will be compared for effectiveness.

The definitions for each other secondary outcome are as follows:

1) Severe malaria: a positive RDT AND one or more of the following:

- Impaired consciousness – Glasgow coma score <3 in children
- Prostration – Generalized weakness so that the person is unable to sit, stand, or walk without assistance
- Multiple convulsions – More than two episodes within 24 hours
- Acidosis – A base deficit of >8 mEq/L, a plasma bicarbonate level of <15 mmol/L, or venous plasma lactate ≥5 mmol/L. Severe acidosis manifests clinically as respiratory distress (rapid, deep, labored breathing).
- Hypoglycemia – Blood or plasma glucose <40 mg/dL (<2.2 mmol/L)
- Severe malarial anemia – Hemoglobin concentration ≤5 g/dL or hematocrit ≤15 percent in children with parasite count >10,000/mcL
- Renal impairment – Plasma or serum creatinine >3 mg/dL (265 mcmol/L) or blood urea >20 mmol/L
- Jaundice – Plasma or serum bilirubin >50 mcmol/L (3 mg/dL) with a parasite count >100,000/mcL
- Pulmonary edema – Radiographically confirmed or oxygen saturation <92 percent on room air with respiratory rate >30/min, often with chest indrawing and crepitations on auscultation
- Significant bleeding – Including recurrent or prolonged bleeding from the nose, gums, or venipuncture sites, hematemesis, or melena
- Shock – Compensated shock is defined as capillary refill ≥3 seconds or temperature gradient on leg (mid to proximal limb) but no hypotension. Decompensated shock is defined as systolic blood pressure <70 mmHg in children, with evidence of impaired perfusion (cool peripheries or prolonged capillary refill).
- *P. falciparum* parasitemia >10 percent (>500,000/mcL)

Severe malaria will be identified through the SAE data. During one distinct SAE, the patient will need to have had a positive RDT result and experienced one or more of the above symptoms.

2) Hospitalizations for malaria: hospitalization at HBCH or other inpatient facility with admitting diagnosis of malaria and microbiologic confirmation of *P. falciparum* infection by RDT.

Hospitalization for malaria will be identified through either the interval visit data or SAE data.

For nonfatal hospitalizations, the patient must have had a unique hospitalization at a particular interval visit that satisfies the following conditions:

- The intake diagnosis for the hospitalization was malaria
- The patient indicated that he/she had a positive RDT result during this hospitalization

For fatal hospitalizations, the hospitalization will be identified through SAE data. The following conditions must be satisfied for one unique SAE:

- Outcome of the event is fatal
- SAE narrative contains “hosp”, “admit”, “admission” or “discharge”
- At least one of the WHO criteria for severe malaria, specified above, is satisfied. All fatal malarias will be severe.
- The patient has a positive RDT for malaria recorded for this SAE

3) LM-positive malaria: the reported presence of *P. falciparum* parasites detected by LM irrespective of RDT or other detection results.

LM-positive malaria will be identified through the malaria testing data. The patient would need to have a LM result that was positive for *P. falciparum* parasitemia.

4) Unconfirmed malaria: the receipt of antimalarials for suspected malaria episodes that were not confirmed by any objective diagnostic test.

Unconfirmed malaria will be restricted to only anti-malarials reported at interval visits. For any hospitalization reported at an interval visit where anti-malarials were prescribed, a check will be done for malaria tests corresponding to this hospitalization. The test must be conducted at a time between one day prior to hospitalization and the day of discharge from hospitalization. If no such test exists, the event will satisfy the conditions for unconfirmed malaria.

5) Fatal malaria: death during hospitalization for malaria, as defined above.

Fatal malaria will be identified through the SAE data. All fatal malarias are severe malarias. The patient must have a SAE that yielded a fatal outcome and reported hospitalization in the narrative of the SAE. For the same event, the patient must also have a positive RDT for malaria and meet at least one of the WHO criteria for severe malaria.

6) Asymptomatic parasitization: the presence of parasites during routine follow-up visits as detected by PCR in patients without fever or a history of recent fever.

Asymptomatic parasitization will be identified through PCR and interval visit data. The patient must not have a history of fever ≥ 37.5**°**C or subjective fever in the past 24 hours of the interval visit.

*This is a lab-generated parasite detection resulting from the dried blood spots collected at each in-person visit. Since these are still generating within the lab, they may be analyzed after the final analysis report has been delivered using the methods as previously described.*

7) Dactylitis: pain or tenderness with or without swelling of the hands or feet.

Dactylitis will be identified through interval visit data. At each visit, the patient is asked for the number of dactylitis episodes experienced since the previous visit.

8) Transfusion: the receipt of red blood cells from any caregiver for any indication.

Transfusions will be identified through interval visit data. At each visit, the patient is asked for the number of transfusions received since the last visit.

9) Acute chest syndrome: a new pulmonary infiltrate and at least 3 of the following findings: chest pain, temperature elevation > 38.5°C, tachypnea, wheezing, or cough.

Acute chest syndrome will be identified through the interval data. At each interval visit, the patient will be asked how many times he/she was hospitalized for acute chest syndrome since the previous visit. It will be assumed that each hospitalization with acute chest syndrome indicated corresponds to one occurrence of acute chest syndrome.

10) All-cause hospitalizations: hospitalization at HBCH or other inpatient facility with any admitting diagnosis.

All-cause hospitalization will be identified through either the interval or SAE data.

For nonfatal hospitalizations, each reported hospitalization per interval visit will be counted as an event.

For fatal hospitalizations, SAE forms with fatal outcomes will be examined. A SAE that satisfies this condition and also contains “hosp”, “admit”, “admission” or “discharge” will be considered an event.

11) All-cause death: death by any cause

All-cause death will be identified through the SAE data. Any SAE with a fatal outcome will satisfy this condition.

12) Severe anemia: Defined as hemoglobin <5.5 g/dL based on lab testing.

Each Hgb <5.5 g/dL during follow-up will be counted as an event.

13) Molecular markers of malaria parasite drug resistance, defined as the presence of single nucleotide polymorphisms (SNPs) in parasite genes that confer resistance to the study drugs.

Parasites detected in dried blood spots from asymptomatic or symptomatic individuals will be subjected to PCR amplification of the genes of interest followed by Sanger sequencing for gene mutations.

*This is a lab-generated parasite detection resulting from the dried blood spots collected at each in-person visit. Since these are still generating within the lab, they may be analyzed after the final analysis report has been delivered using the methods as previously described.*

# 5. Analysis Methods

## 5.1 General Principles

In addition to specific analyses and presentations that are detailed in the following sections, results will be summarized by treatment using descriptive statistics, including the number of patients, mean, standard deviation, median, 25^th^ and 75^th^ percentiles, and range as appropriate. For categorical variables, count and percentage by treatment group will be presented.

Summaries of continuous variables will be based on non-missing observations. Percentages for categorical variables will be calculated based on number of patients with non-missing values for the variable.

For tabulations of changes from baseline or shift analyses, if multiple measurements are obtained at the same visit, then the measurement obtained on the day closest to the target day will be used in the analysis. In the case of a tie, the measurement obtained on the earlier day will be used. In the case of multiple measurements on the same day and time, the worst value will be used.

Unless otherwise specified, all statistical tests will be two-sided with a significance level of 0.05. All statistical analyses will be conducted using SAS version 9.4 or higher (SAS Institute Inc., Cary, NC, USA)

## 5.2 Censoring

In secondary analyses involving a dichotomous outcome, cumulative incidence at 12 months will be reported for each endpoint to allow for the competing risk of death, as well as right censoring at the date of last contact where all elements of the outcome can be assessed.

## 5.3 Handling of Missing Data

Subjects will be followed for up to 12 months for both the primary and secondary endpoints. The sample size allows up to 20% of the subjects to be lost to follow-up and still maintain adequate statistical power. Methods to reduce loss to follow-up will be employed by the study team, including telephone calls and, if necessary, home visits; however, not all subjects will be followed for the entire study period. Subjects may have missing data during some of the follow-up time points; however, the generalized linear models used for the longitudinal primary and secondary endpoints will allow for any data collected to be utilized for analysis. Thus, no imputation of baseline data or endpoints will be done. The tables will be generated from complete case data only.

## 5.4 Statistical Hypotheses

The primary outcome of the study is clinical malaria, which will be expressed as an incidence rate (IR; the number of episodes per patient-year at risk of malaria) and will be evaluated through the 12-month follow-up visit for effectiveness.

The null hypothesis for the primary endpoint is that there is no treatment difference between each of the experimental arms (monthly SP-AQ and monthly DP) compared to the standard-of-care (SOC) arm (daily Proguanil) from randomization to 12 months. The alternative hypothesis is that each of the experimental arms has a better treatment response (i.e., lower IR of malaria) compared to the SOC arm through 12 months of treatment and follow-up.

Let IR**_SP_** and IR**_DP_** be the expected number of episodes per patient-year at risk (incidence rate) through 12-months for SP-AQ and DP arms, respectively, and IR**_C_** the rate for the SOC arm. The null and alternative hypotheses are as follows:

H_O_: IR**_SP_** = IR**_C_**  vs. H_A_: IR**_SP_** ≠ IR**_C_,** *and*

H_O_: IR**_DP_** = IR**_C_**  vs. H_A_: IR**_DP_** ≠ IR**_C_**.

## 5.5 Analysis of the Primary Outcome

Incidence rates for each experimental arm in the AT population will be compared to the control group using a generalized regression model that allows for interdependence between multiple episodes within the same participant that is unadjusted for potential covariates. Models considered for the count data will include the Poisson, negative-binomial and zero-inflated models with the final model determined by the smaller residual deviance. Each regression model will provide incidence rate ratios (IRR) with 95% confidence intervals (CI) for each experimental treatment group compared to the control arm.

The primary outcome of malaria will be expressed as an incidence rate (IR), or the number of episodes per patient-year at risk through 12-months. IRs will be computed after subtracting two weeks from the time at risk for each episode, in order to avoid counting an episode more than once.

IRs of malaria will be primarily compared between each experimental treatment group and the control arm using a generalized regression model. The model will be unadjusted and will utilize the AT population. The regression model will allow interdependence between episodes within the same individual (i.e., clustering by subject). This will create robust standard errors for the parameter estimates, and also allow the use of actual follow-up time as the offset variable

The treatment difference for each hypothesis test will be tested using the contrast between treatment groups under the regression model. These differences will be presented as incidence rate ratios per patient-year at risk with 95% confidence intervals for each experimental treatment group compared to the control arm. In order to control the nominal type-I error rate (α=0.05), each test and corresponding confidence interval will use an α=0.0269 per Dunnett’s test for multiple comparisons to the same control arm.

*Similar analyses for the ITT and ATP population will also be evaluated.*

The number and cumulative incidence rate for patients who experience the primary endpoint will also be reported. Death will be considered a competing risk and patients will be censored at the last known follow-up date. The ITT population will be used for dichotomous endpoints.

## 5.6 Analysis of the Secondary Outcomes

The secondary outcomes will each be expressed as a dichotomous response (yes/no) during follow-up and compared between treatment arms using a Kaplan-Meier method to allow death to be a competing risk and censor for patients lost to follow-up using the ITT and ATP population. Additionally, for outcomes in which more than one event is possible, event rates will be compared using a generalized regression model to evaluate incidence rates similar to that used for the primary outcome based on the AT population. Since the event rates are likely to be very small, the following composite outcomes will also be compared: (1) dactylitis or painful events; (2) receipt of blood products (transfusions), acute chest syndrome (ACS), hospitalization, or death; and (3) *any* secondary outcome (with or without molecular markers of drug resistance or parasitization based on availability of lab data at time of analysis).

| **Outcome** | **Event rate** | **Cumulative incidence** |
| --- | --- | --- |
| Painful events | Yes | Yes |
| Severe malaria | Yes | Yes |
| Hospitalization for malaria | Yes | Yes |
| LM-positive malaria | Yes | Yes |
| Unconfirmed malaria | Yes | Yes |
| Fatal malaria | No | Yes |
| Asymptomatic parasitization | Yes | Yes |
| Dactylitis | Yes | Yes |
| Transfusion | Yes | Yes |
| Acute chest syndrome | Yes | Yes |
| All-cause hospitalization | Yes | Yes |
| All-cause death | No | Yes |
| Severe anemia | Yes | Yes |
| Molecular markers of parasite drug resistance | Yes | Yes |

## 5.7 Sub-Group Analyses

We will explore the impact of COVID-19 and protocol changes by including an interaction term (i.e., pre/post-COVID-19) with treatment in the models for the primary and main secondary endpoint evaluating event rates. This will be a sub-group analysis using the AT population. Other secondary endpoints may be considered based on viewing the initial results.

Additionally, we will explore the effect of the treatment arm on the two main outcomes (clinical malaria and painful events) in the AT population within these pre-specified subgroups defined using baseline data:

1. Age at enrollment (1-5 years vs >5 years)
2. Hydroxyurea use (Yes vs No)
3. Sex (Girls vs Boys)
4. Reported hospitalization in the prior 12 months (Yes vs No)
5. Hemoglobin concentration (<7, 7 – 9 or >9 g/dL)

# 6 Safety and Tolerability Analysis

## 6.1 Serious Adverse Events (SAEs)

Only serious adverse events (SAEs) will be recorded in the data. Free text taken from the CRF will be used to identify SAEs, with site staff asked to review the terms for consistency at weekly data reviews. The project leader and site staff will be responsible for inclusion of any keywords corresponding to endpoints (including notes about hospitalization) within the SAE narrative. Counts of occurrences and percentages of patients experiencing each SAE type will be reported by treatment group, sorted by descending frequency in alphabetical order.

Counts and percentages describing seriousness, actions taken as a result of SAEs, outcomes, and relationship to study drug will also be reported by treatment. The number and percentages of patients experiencing any SAE, as well as the number of SAEs of any type will be reported by treatment. No hypothesis tests will be conducted for SAEs.

SUSARs, if present, will be described in the same manner as SAEs.

## 6.2 Stopping Rules

Study stopping guidelines are designed to detect an imbalance of participant deaths in either intervention arm (SP-AQ or DP) compared to the SOC arm (Proguanil). For this purpose, sequential boundaries will be used to continuously monitor the rate of participant deaths in each treatment arm compared to the SOC arm. Accrual into a treatment arm will be considered for stopping if the difference in the number of deaths between that treatment arm and SOC arm is equal to or exceeds *b_n_* out of *n* enrolled participants in the treatment arm (see table below). This is based on a Pocock-type stopping boundary that yields the probability of crossing the boundary when the difference in the rate of death is equal to or exceeds 5%.

| ***5%*** | ***n*** | 2-5 | 6-14 | 15-24 | 25-36 | 37-48 | 49-61 | 62-74 | 75-82 |
| --- | --- | --- | --- | --- | --- | --- | --- | --- | --- |
|  | ***b_n_*** | 2 | 3 | 4 | 5 | 6 | 7 | 8 | 9 |

Assuming a maximum planned sample size of 82 in each of the three study arms and a desired probability of stopping owing to chance of 0.10, a treatment arm (SP-AQ or DP) will be considered for stopping if the difference in the number of deaths between the treatment arm and the SOC arm is equal to or exceeds *b_n_* out of *n* enrolled participants in each of the two arms.

The stopping rules assume an equal number of participants in the treatment and SOC arms being compared when a death occurs. If the numbers within the arms are imbalanced, the confidence interval should be verified by the study statistician prior to the final decision to stop a treatment arm. The timing of events will be considered through Kaplan-Meier plots before the final decision is made.

## 6.3 Laboratory Data

Descriptive statistics will be provided for laboratory measurements which may include, but may not be restricted to the measurements below. This will include measurements taken at baseline and each scheduled (or reported) time point as well as changes from baseline by treatment. Refer to **§1.5 Timing of Major Assessments** for the timing of lab assessments

- White blood cell count (10^9^/L)
- Neutrophil count (10^9^/L)
- Hemoglobin (g/L)
- Platelet count (10^9^/L)
- Mean corpuscular volume (fl)
- HbF (%)
- HbS (%)
- ALT/SGPT (units/L)
- Creatinine (mmol/L)
- QTcF interval (msec)

Laboratory abnormalities will be reported. The number and percentage of patients experiencing at least one incidence of a laboratory abnormality will also be reported by treatment group. The percentages will be calculated relative to the number of patients who have at least one incidence of the specified lab measurement in each group.

In addition, in the population of patients experiencing at least one laboratory abnormality, the number of abnormalities per patient will be reported.

## 6.4 ECG Data

All participants will have a screening ECG to determine eligibility based on QTcF.

In addition, ECG assessments will be taken 4-6 hours following the final dose of monthly DP for up to the first 20 patients assigned to DP living in the town of Homa Bay, for the purposes of measuring the QTcF. In these children, QTcF will be measured at screening and following each interval visit. The QTcF measurement used will be the last observation per visit. In many patients, this will be the only measurement taken. In patients whose first measurement exceeds 450 msec, a repeat ECG will be taken and the second measurement will be used for analysis. The change from the screening measurement will be reported at enrollment and all interval visits by treatment.

QTcF measurements following receipt of DP will be analyzed in two ways. Firstly, as the number of patients and as the number of ECG measurements with a post-DP QTcF >450 msec. Secondly, we will express for each post-DP ECG the change in QTcF from the baseline measurement, and then as the number of patients with a change from baseline of >50 msec.

## 6.5 Baseline Medications and Interventions

A baseline medication is defined as a medication that was reported on the medical history form at the enrollment visit. Patients taking baseline medications of interest will be summarized by treatment group in the ITT population.

All patients aged between 1 and 5 years will receive daily penicillin prophylaxis.

## 6.6 Vitals and Physical Assessments

Descriptive statistics will be provided by treatment group at baseline for desired vitals and physical assessments using the ITT population.

6.7 Concomitant Medications Concomitant medications of interest taken by patients will be reported. The number of patients taking each medication, time initiated (relative to baseline), current medication status, and duration of medication use will be reported for each medication.

## 7 DSMB and Interim Analyses

## 7.1 Interim Analyses

A Data and Safety Monitoring Committee (DSMB) will review safety data, including a report of primary and secondary outcomes at least every 6 months. The DSMB can recommend continuing the study as planned, or stop for overwhelming efficacy or safety signal.

Prior to each meeting, the DCRI will conduct any requested statistical analyses and prepare a summary report along with the following information: patient enrollment reports, rates of compliance with the assigned testing strategy, frequency of protocol violations, and descriptions of SUSARs. The extracted data files and analysis programs for each DSMB report will be archived and maintained at the DCRI for the life of the study.

For ethical reasons, an interim examination of key safety data will be performed when the study has accrued approximately ½ of the estimated total person-time. The primary objective of this analysis will be to evaluate the accumulated data for high frequency of negative clinical outcomes in either of the three randomized arms. In addition, the periodic monitoring will review the standard-of-care arm event rates, patient recruitment, compliance with the study protocol, status of data collection, and other factors that reflect the overall progress, data quality, and integrity of the study. The results of the interim analysis and monitoring reports will be carefully and confidentially reviewed by the DSMB. The DSMB will then give their recommendations for continuing or stopping the study to the PIs.

See DSMB table shells for expected output that will be provided for each DSMB meeting.
