# Supplementary material for: Monthly sulfadoxine/pyrimethamine-amodiaquine or dihydroartemisinin-piperaquine as malaria chemoprevention in young Kenyan children with sickle cell anemia: A randomized controlled trial
Source: PLoS Med. 2022 Oct 10;19(10):e1004104. doi: 10.1371/journal.pmed.1004104 (PMC9591057; doi:10.1371/journal.pmed.1004104)
Supplement: S1 Text — Supplemental methods, figure, and tables. (DOCX) [file pmed.1004104.s004.docx]

**Supplementary Appendix**

**This appendix accompanies the following manuscript:**

Monthly sulfadoxine/pyrimethamine-amodiaquine or dihydroartemisinin-piperaquine as malaria chemoprevention in young Kenyan children with sickle cell anemia: A randomized controlled trial

Steve M Taylor, Sarah Korwa, Angie Wu, Cynthia L Green, Betsy Freedman, Sheila Clapp, Joseph Kipkoech Kirui, Wendy P O’Meara, Festus M Njuguna

**Table of Contents**

[Supplemental Methods 3](#_Toc113270924)

[Additional sample size considerations 3](#_Toc113270925)

[Outcome definitions 3](#_Toc113270926)

[Dosing of study-provided medications 5](#_Toc113270927)

[Molecular parasite detection 6](#_Toc113270928)

[Human genotyping 7](#_Toc113270929)

[Supplemental Figure 8](#_Toc113270930)

[Figure A. Mean QTcF Measurements Among DP Recipients by Month 8](#_Toc113270931)

[Supplemental Tables 9](#_Toc113270932)

[Table A. Adherence to Chemoprevention Regimen 9](#_Toc113270933)

[Table B. Outcome Comparisons Between SP-AQ and DP in the As-Treated Population 10](#_Toc113270934)

[Table C. Secondary Outcomes in the Intention-To-Treat Population 12](#_Toc113270935)

[Table D. Main Hematologic Outcomes According to Subgroups in the As-Treated Population 14](#_Toc113270936)

[Table E. Serious Adverse Events in the Intention-To-Treat population 17](#_Toc113270937)

[Table F. Change from Baseline in Neutrophil Count (10^9^/L) in the Intention-to-Treat Population 19](#_Toc113270938)

[Table G. Change from Baseline in Platelet Count (10^9^/L) in the Intention-to-Treat Population 21](#_Toc113270939)

[Table H. G6PD and CYP2C8*2 Alleles in 10 Participants Who Died 23](#_Toc113270940)

[Table I. QTcF Measurements in DP Recipients and Change from Baseline 24](#_Toc113270941)

[References 25](#_Toc113270942)

# Supplemental Methods

## Additional sample size considerations

As described in the protocol (Section 9.5), the sample size was computed based upon the rate of observed events of clinical malaria among children aged up to 17m participating in a randomized trial of the RTS,S/AS01 malaria vaccine in Siaya district. The observed event rate was 3.7 episodes per year per child. This was considered a suitable estimate of the baseline event rate of the primary outcome amongst recipients of the standard of care daily Proguanil for several reasons:

1. Malaria transmission is historically similar between Siaya and Homa Bay districts;
2. The expected mean age of enrollment of children with SCA was expected to be 2-3 years of age;
3. Because the vaccine efficacy was ultimately reported at 43%, and the observed rate represents an aggregate of vaccinated and unvaccinated children, this mild decrease from the true event rate could feasibly approximate the mild protection offered by daily Proguanil.

## Outcome definitions

The primary endpoint was incidence of clinical malaria expressed as episodes per person-year at risk, defined as:

- - Uncomplicated malaria:
    - Presence of *P. falciparum* parasitemia of any density detected by malaria rapid diagnostic test (RDT) AND
    - Temperature of ≥ 37.5C or history of objective or subjective fever in the preceding 24 hours

OR

- Case meeting the definition of severe malaria according to WHO criteria,[1] defined as a positive malaria RDT AND one or more of the following:

● Impaired consciousness – Glasgow coma score <3 in children

● Prostration – Generalized weakness so that the person is unable to sit, stand, or walk without assistance

● Multiple convulsions – More than two episodes within 24 hours

● Acidosis – A base deficit of >8 mEq/L, a plasma bicarbonate level of <15 mmol/L, or venous plasma lactate ≥5 mmol/L. Severe acidosis manifests clinically as respiratory distress (rapid, deep, labored breathing).

● Hypoglycemia – Blood or plasma glucose <40 mg/dL (<2.2 mmol/L)

● Severe malarial anemia – Hemoglobin concentration ≤5 g/dL or hematocrit ≤15 percent in children with parasite count >10,000/mcL

● Renal impairment – Plasma or serum creatinine >3 mg/dL (265 mcmol/L) or blood urea >20 mmol/L

● Jaundice – Plasma or serum bilirubin >50 mcmol/L (3 mg/dL) with a parasite count >100,000/mcL

● Pulmonary edema – Radiographically confirmed or oxygen saturation <92 percent on room air with respiratory rate >30/min, often with chest indrawing and crepitations on auscultation

● Significant bleeding – Including recurrent or prolonged bleeding from the nose, gums, or venipuncture sites, hematemesis, or melena

● Shock – Compensated shock is defined as capillary refill ≥3 seconds or temperature gradient on leg (mid to proximal limb) but no hypotension. Decompensated shock is defined as systolic blood pressure <70 mmHg in children, with evidence of impaired perfusion (cool peripheries or prolonged capillary refill).

● *P. falciparum* parasitemia >10 percent (>500,000/mcL)

*Ascertainment*: Signs of severe malaria were captured during routine or acute care clinic visits.

Secondary outcomes and their definitions and ascertainment were:

1. Severe malaria, defined as above [1]:
2. Hospitalization for malaria, defined as hospitalization at HBCH or other inpatient facility with admitting diagnosis of malaria and microbiologic confirmation of *P. falciparum* infection by RDT.

*Ascertainment*: Review of HBCH pediatric ward admission logs and query of caregivers at follow-up visits.

1. Light microscopy (LM)-positive malaria, defined as the reported presence of *P. falciparum* parasites detected by LM irrespective of RDT or other detection results.

*Ascertainment*: Review of HBCH and other healthcare facility records.

1. Unconfirmed malaria, defined as the receipt of antimalarials for suspected malaria episodes that were not confirmed by any objective diagnostic test.

*Ascertainment*: Review of HBCH and other healthcare facility records.

1. Fatal malaria, defined as death during hospitalization for malaria, as defined above.

*Ascertainment*: Review of HBCH pediatric ward inpatient logs and query of caregivers at follow-up visits.

1. Asymptomatic parasitization, defined as the presence of parasites during routine follow-up visits as detected by PCR in patients without fever or a history of recent fever.

*Ascertainment*: Dried blood spots collected during routine visits from all participants and tested post-hoc for the presence of *P. falciparum* parasites using a real-time PCR assay.

1. Painful events, defined as pain lasting 2 hours or more without obvious cause (either as inpatients or outpatients) [2].

*Ascertainment*: Query using structured interview at each clinic visit.

1. Dactylitis, defined as pain or tenderness with or without swelling of the hands or feet [2].

*Ascertainment*: Query using structured interview at each clinic visit.

1. Transfusions, defined as the receipt of red blood cells from any caregiver for any indication.

*Ascertainment*: Query using structured interview at each clinic visit.

1. Severe anemia, defined as hemoglobin concentration < 5.5 g/dL

*Ascertainment*: Hemoglobin measurement by either venous complete blood count or capillary point-of-care test

1. Acute chest syndrome, defined as a new pulmonary infiltrate and at least 3 of the following findings: chest pain, temperature elevation > 38.5°C, tachypnea, wheezing, or cough.

*Ascertainment*: Structured history and physical exam at each scheduled and acute care visit, or review of outside records.

1. All-cause hospitalizations, defined as hospitalization at HBCH or other inpatient facility with any admitting diagnosis.

*Ascertainment*: Query using structured interview at each clinic visit.

1. All-cause deaths, defined as death by any cause.

*Ascertainment*: Query using structured interview during follow-up.

## Dosing of study-provided medications

Dosing of daily proguanil was targeted to approximately 3mg/kg/day, consistent with clinical practice and current recommendations in Kenya, rounded to the nearest 25mg increment [3]:

| **Weight (kg)** | **No. of 100mg tabs Proguanil daily** |
| --- | --- |
| ≤ 13 | ¼ |
| 13-21 | ½ |
| 21-29 | ¾ |
| > 29 | 1 |

Dosing of SP-AQ was consistent with WHO recommendations for SMC with SP-AQ in West Africa [4]:

| **Age (y)** | **Day 1** | **Day 2** | **Day 3** |
| --- | --- | --- | --- |
| 1-5 | 1 tablet 500/25mg SP  1 tablet 150mg AQ | 1 tablet 150mg AQ | 1 tablet 150mg AQ |
| 6-10 | 1.5 tablets 500/25mg SP  1.5 tablets 150mg AQ | 1.5 tablets 150mg AQ | 1.5 tablets 150mg AQ |

The weight-based dosing of tablets of 40/320mg of DP was:

| **Weight (kg)** | **No. of 40/320mg tabs DP daily for 3 days** |
| --- | --- |
| ≤ 5 | ¼ |
| 6-10 | ½ |
| 11-14 | ¾ |
| 15-19 | 1 |
| 20-23 | 1 ¼ |
| 24-25 | 1 ½ |
| 26-35 | 2 |
| 36-59 | 3 |

Children under 5 years of age received daily penicillin prophylaxis with oral Penicillin V:

| **Age (y)** | **No. of 125mg tabs BID** |
| --- | --- |
| 1-3 | 1 |
| >3-5 | 2 |

Children with RDT-positive malaria received weight-based AL as per national guidelines for uncomplicated falciparum malaria [3]**:**

| **Weight (kg)** | **No. of 20/120mg tabs BID for 3 days** |
| --- | --- |
| 5-14 | 1 |
| 15-24 | 2 |
| 25-34 | 3 |
| >34 | 4 |

## Molecular parasite detection

From each dried blood spot (DBS), 1 8mm punch was deposited into a single well of a deepwell plate. Punched DBS were first incubated overnight with 0.5% Saponin at 4C and then overnight with Proteinase K at 56C, and then genomic DNA (gDNA) isolated with 7% Chelex with boiling, and ultimately eluted into approximately 125uL and stored at -20C.

Each gDNA extract was tested in duplicate for *P. falciparum* parasites in a duplex real-time PCR reaction targeting the parasite *pfr364* motif and human β-globin. Each 12µL reaction consisted of 1µL of template, 6µL of TaqMan Environmental MasterMix, 250nM each of *pfr364* primers, 300nM each of β-tubulin primers, and 300nM each of *pfr364* and β-tubulin probes labeled with FAM and VIC, respectively. Reactions were performed on 384-well plates on an ABI QuantStudio6 platform, and each plate included a panel of quantitative controls of gDNA extracted in parallel from DBS containing *P. falciparum* parasites in concentrations from 2000 parasites/µL to 0.1 parasite/µL. These controls were also tested in duplicate, and standard curves were computed from their cycle threshold (Ct) values. We defined a *P. falciparum*-positive sample as one in which either replicate amplified with a Ct value <38 or in which both replicates amplified with any Ct value.

## Human genotyping

The CYP2C8*2 locus was genotyped using PCR amplification and Sanger sequencing. Five µL of purified genomic DNA was amplified in a 25µL reaction including 0.5µL of KAPA HiFi Hotstart DNA polymerase, 5µL 5X KAPA HiFi Fidelity Buffer, 0.75µL of dNTPs, and forward and reverse primers at a final concentration of 200nM. The forward primer CYP2C8-2F was GAACACCAAGCATCACTGGA and the reverse primer CYP2C8-2R was GAAATCAAAATACTGATCTGTTGC [5]. Cycling conditions were 95C x 3m, followed by 30 cycles of 98C x 20s, 63C x 15s, and 72C x 15s, followed by a final extension at 72C for 1m. Products were electrophoresced on agarose gels, the 107nt product was bi-directionally sequenced using amplification primers, and reads were aligned to reference sequence NG007972.1 and manually scored using SeqMan Ultra (v17, DNASTAR, Madison, WI).

The G6PD locus was amplified and sequenced across the loci encoding variants at amino acid positions 68 and 126. Four µL of purified genomic DNA was amplified in a 25µL reaction including 0.5µL of KAPA HiFi Hotstart DNA polymerase, 5µL 5X KAPA HiFi Fidelity Buffer, 0.75µL of dNTPs, and forward and reverse primers at a final concentration of 200nM. The forward primer was GTGGCTGTTCCGGGATGGCCTTCTG and the reverse primer was AGGGCAACGGCAAGCCTTAC [6]. Cycling conditions were 95C x 3m, followed by 32 cycles of 98C x 20s, 74C x 15s, and 72C x 15s, followed by a final extension at 72C for 30s. Products were electrophoresced on agarose gels for visual confirmation of the 897nt product which contained both loci. This product was bi-directionally sequenced, reads were aligned to reference sequence NC000023.11, and manually scored using SeqMan Ultra at both positions. Results were classified as follows:

| **202 allele** | **Codon 68** | **376 allele** | **Codon 126** | **G6PD type** |
| --- | --- | --- | --- | --- |
| G | V | A | N | B |
| G | V | G | D | A+ |
| A | M | A | N | Unknown |
| A | M | G | D | A- |

# Supplemental Figure

## Figure A. Mean QTcF Measurements Among DP Recipients by Month

Month 0 measurement is baseline, and other month measurements are following the 3^rd^ and final dose of the monthly DP course. Dots indicate mean, and bars the standard deviation.

QTcF: Fridericia’s corrected QT interval; DP: Dihydroartemisinin/Piperaquine; msec: millisecond; CI: confidence interval

# Supplemental Tables

## Table A. Adherence to Chemoprevention Regimen

|  | **Daily Proguanil**  **(N=81)** | **Monthly SP-AQ**  **(N=83)***** | **Monthly DP**  **(N=82)** |
| --- | --- | --- | --- |
| Missed any doses, % (n/N)* |  |  |  |
| Month 1 | 6.2 (5/81) | 1.3 (1/75) | 0 (0/80) |
| Month 2 | 3.8 (3/80) | 2.8 (2/72) | 0 (0/77) |
| Month 3 | 3.8 (3/78) | 1.5 (1/65) | 0 (0/78) |
| Month 4 | 3.9 (3/77) | 1.6 (1/64) | 0 (0/75) |
| Month 5 | 2.6 (2/76) | 3.3 (2/60) | 2.7 (2/75) |
| Month 6 | 2.6 (2/76) | 8.5 (5/59) | 0 (0/73) |
| Month 7 | 2.6 (2/76) | 2.0 (1/51) | 0 (0/74) |
| Month 8 | 6.6 (5/76) | 13.0 (7/54) | 0 (0/72) |
| Month 9 | 4.1 (3/74) | 3.6 (2/55) | 0 (0/72) |
| Month 10 | 2.7 (2/74) | 1.9 (1/52) | 1.4 (1/72) |
| Month 11 | 2.7 (2/74) | 7.4 (4/54) | 1.4 (1/72) |
| Month 12 | 0 (0/75) | 0 (0/54) | 2.8 (2/72) |
| Ever difficulties getting the child to take the medication, % (n)** | 3.7 (3) | 27.5 (22) | 6.3 (5) |
| Ever forgot to give medications, %, (n)** | 16.0 (13) | 2.5 (2) | 0 (0) |

* For Proguanil, any daily doses in prior 14 days; for SP-AQ and DP, any doses of the prior month’s regimen.

** Across all months while receiving the specified treatment

*** Excludes responses during months in which children allocated to SP-AQ received Proguanil

SP-AQ: Sulfadoxine/Pyrimethamine-Amodiaquine; DP: Dihydroartemisinin/Piperaquine

## Table B. Outcome Comparisons Between SP-AQ and DP in the As-Treated Population

|  | **Monthly DP**  **(n=82)** | **Monthly SP-AQ**  **(n=83)** | **Incidence Rate Ratio or**  **Hazard Ratio**** | |
| --- | --- | --- | --- | --- |
|  |  |  | **95% CI** | **P-value** |
| **Patient-years follow-up** | **69.3** | **56.7** |  |  |
| **Main Outcomes** |  |  |  |  |
| **Clinical malaria** |  |  |  |  |
| Number of events | 3 | 3 |  |  |
| Incidence rate per patient-year (95% CI) | 0.04  (0.01 – 0.13) | 0.09  (0.02 – 0.39) | 2.24  (0.23 – 21.61) | 0.66 |
| **Painful events** |  |  |  |  |
| Number of events | 286 | 238 |  |  |
| Incidence rate per patient-year (95% CI) | 4.18  (3.19 – 5.46) | 4.28  (3.38 – 5.42) | 1.02  (0.66 – 1.58) | 0.99 |
| **Secondary Outcomes** |  |  |  |  |
| **Severe malaria** |  |  |  |  |
| Incidence rate per patient-year (95% CI) | 0 | 0 | NA | NA |
| **Hospitalized for malaria** |  |  |  |  |
| Number of events | 3 | 2 |  |  |
| Incidence rate per patient-year (95% CI) | 0.08  (0.02 – 0.32) | 0.02  (0.01 – 0.11) | 0.32  (0.03 – 3.74) | 0.50 |
| **Light-microscopy-positive malaria** |  |  |  |  |
| Number of events | 19 | 10 |  |  |
| Incidence rate per patient-year (95% CI) | 0.25  (0.15 – 0.42) | 0.15  (0.07 – 0.31) | 0.59  (0.20 – 1.76) | 0.47 |
| **Unconfirmed malaria** |  |  |  |  |
| Number of events | 40 | 21 |  |  |
| Incidence rate per patient-year (95% CI) | 0.57  (0.40 – 0.82) | 0.45  (0.26 – 0.81) | 0.80  (0.35 – 1.80) | 0.77 |
| **Fatal malaria** |  |  |  |  |
| Cumulative incidence rate (95% CI) | 0 | 0 | NA | NA |
| **Asymptomatic *P falciparum* infection** |  |  |  |  |
| Number of events | 9 | 15 |  |  |
| Incidence rate per patient-year (95% CI) | 0.12  (0.06 - 0.25) | 0.27  (0.14 - 0.52) | 2.23  (0.68 – 7.29) | 0.23 |
| **Dactylitis** |  |  |  |  |
| Number of events Count | 37 | 45 |  |  |
| Incidence rate per patient-year (95% CI) | 0.51  (0.33 – 0.78) | 0.84  (0.53 – 1.35) | 1.66  (0.77 – 3.56) | 0.24 |
| **Severe anemia*** |  |  |  |  |
| Number of events | 4 | 8 |  |  |
| Incidence rate per patient-year (95% CI) | 0.06  (0.02 – 0.16) | 0.14  (0.07 – 0.29) | 2.46  (0.57 – 10.68) | 0.30 |
| **Transfusion of blood products** |  |  |  |  |
| Number of events | 4 | 5 |  |  |
| Incidence rate per patient-year (95% CI) | 0.19  (0.04 – 0.99) | 0.09  (0.03 – 0.30) | 0.48  (0.04 – 5.42) | 0.73 |
| **Acute chest syndrome** |  |  |  |  |
| Number of events | 0 | 3 |  |  |
| Incidence rate per patient-year (95% CI) | 0 | 0.05  (0.01 – 0.20) | NA | NA |
| **All-cause hospitalization** |  |  |  |  |
| Number of events | 40 | 43 |  |  |
| Incidence rate per patient-year (95% CI) | 0.78  (0.44 – 1.37) | 1.28  (0.54 – 2.99) | 1.64  (0.48 – 5.63) | 0.59 |
| **Death** |  |  |  |  |
| Cumulative incidence | 1 | 7 |  |  |
| Cumulative incidence rate (95% CI) | 1.3%  (0.2 – 9.1) | 11.3%  (6.1 – 20.7) | 8.86  (0.74 – 106.55) | 0.10 |

* Defined as hemoglobin concentration <5.5 grams/deciliter

**Incidence rate ratio or hazard ratio for SP-AQ relative to DP using Tukey’s post-hoc adjustment for multiple pairwise comparisons assuming all three pairwise comparisons were of interest.

SP-AQ: Sulfadoxine/Pyrimethamine-Amodiaquine; DP: Dihydroartemisinin/Piperaquine; CI: confidence interval

## Table C. Secondary Outcomes in the Intention-To-Treat Population

|  | **Overall (N=246)** | **Daily Proguanil (N=81)** | **Monthly SP-AQ (N=83)** | **Monthly DP (N=82)** |
| --- | --- | --- | --- | --- |
| **Patient years follow-up** | **208.5** | **71.6** | **67.6** | **69.3** |
| **Parasitologic** |  |  |  |  |
| **Severe malaria** |  |  |  |  |
| Incidence rate per patient-year (95% CI) | 0 | 0 | 0 | 0 |
| **Hospitalization for malaria** |  |  |  |  |
| Number of events | 7 | 3 | 3 | 3 |
| Incidence rate per patient-year (95% CI) | 0.03 (0.02 - 0.07) | 0.03 (0.01 - 0.11) | 0.03 (0.01 - 0.12) | 0.04 (0.01 - 0.13) |
| Incidence rate ratio (95% CI) |  |  | 1.06 (0.12 - 9.46) | 1.55 (0.21 - 11.48) |
| p-value |  |  | >0.99 | 0.84 |
| **LM-positive malaria** |  |  |  |  |
| Number of events | 47 | 17 | 11 | 19 |
| Incidence rate per patient-year (95% CI) | 0.22 (0.16 - 0.31) | 0.24 (0.14 - 0.41) | 0.16 (0.09 - 0.30) | 0.27 (0.16 - 0.45) |
| Incidence rate ratio (95% CI) |  |  | 0.69 (0.27 - 1.73) | 1.15 (0.49 - 2.70) |
| p-value |  |  | 0.57 | 0.90 |
| **Unconfirmed malaria** |  |  |  |  |
| Number of events | 107 | 42 | 25 | 40 |
| Incidence rate per patient-year (95% CI) | 0.51 (0.41 - 0.63) | 0.58 (0.41 - 0.83) | 0.37 (0.23 - 0.58) | 0.58 (0.42 - 0.80) |
| Incidence rate ratio (95% CI) |  |  | 0.63 (0.33 - 1.21) | 0.99 (0.58 - 1.70) |
| p-value |  |  | 0.21 | >0.99 |
| **Asymptomatic *P. falciparum* parasitization** |  |  |  |  |
| Number of events | 74 | 45 | 20 | 9 |
| Incidence rate per patient-year (95% CI) | 0.35 (0.25 - 0.51) | 0.62 (0.36 - 1.06) | 0.30 (0.20 - 0.47) | 0.13 (0.06 - 0.26) |
| Incidence rate ratio (95% CI) |  |  | 0.49 (0.22 - 1.06) | 0.21 (0.08 - 0.56) |
| p-value |  |  | 0.074 | <0.001 |
| **Hematologic** |  |  |  |  |
| **Dactylitis** |  |  |  |  |
| Number of events | 180 | 86 | 57 | 37 |
| Incidence rate per patient-year (95% CI) | 0.87 (0.65 - 1.17) | 1.20 (0.72 - 1.99) | 0.88 (0.56 - 1.38) | 0.53 (0.36 - 0.80) |
| Incidence rate ratio (95% CI) |  |  | 0.73 (0.34 - 1.57) | 0.45 (0.22 - 0.93) |
| p-value |  |  | 0.56 | 0.028 |
| **Severe anemia*** |  |  |  |  |
| Number of events | 21 | 8 | 9 | 4 |
| Incidence rate per patient-year (95% CI) | 0.10 (0.07 - 0.15) | 0.11 (0.06 - 0.22) | 0.13 (0.07 - 0.25) | 0.06 (0.02 - 0.15) |
| Incidence rate ratio (95% CI) |  |  | 1.19 (0.42 - 3.38) | 0.52 (0.14 - 1.94) |
| p-value |  |  | 0.91 | 0.44 |
| **Transfusion of blood products** |  |  |  |  |
| Number of events | 24 | 13 | 7 | 4 |
| Incidence rate per patient-year (95% CI) | 0.13 (0.07 - 0.23) | 0.18 (0.07 - 0.45) | 0.12 (0.05 - 0.28) | 0.07 (0.02 - 0.22) |
| Incidence rate ratio (95% CI) |  |  | 0.64 (0.15 - 2.75) | 0.38 (0.07 - 2.07) |
| p-value |  |  | 0.73 | 0.35 |
| **Acute chest syndrome** |  |  |  |  |
| Number of events | 4 | 1 | 3 | 0 |
| Incidence rate per patient-year (95% CI) | 0.02 (0.01 - 0.06) | 0.01 (0.00 - 0.10) | 0.04 (0.01 - 0.18) | 0 |
| Incidence rate ratio (95% CI) |  |  | 3.07 (0.21 - 44.62) |  |
| p-value |  |  | 0.50 |  |
| **General** |  |  |  |  |
| **All-cause hospitalization** |  |  |  |  |
| Number of events | 139 | 47 | 52 | 40 |
| Incidence rate per patient-year (95% CI) | 0.68 (0.55 - 0.84) | 0.66 (0.46 - 0.94) | 0.79 (0.59 - 1.05) | 0.59 (0.37 - 0.94) |
| Incidence rate ratio (95% CI) |  |  | 1.20 (0.71 - 2.02) | 0.90 (0.47 - 1.75) |
| p-value |  |  | 0.67 | 0.92 |

* Defined as hemoglobin concentration <5.5 grams/deciliter

SP-AQ: Sulfadoxine/Pyrimethamine-Amodiaquine; DP: Dihydroartemisinin/Piperaquine; CI: confidence interval

## Table D. Main Hematologic Outcomes According to Subgroups in the As-Treated Population

|  | **Overall** | **Daily Proguanil** | **Monthly SP-AQ** | | | **Monthly DP** | | | |
| --- | --- | --- | --- | --- | --- | --- | --- | --- | --- |
|  | **Incidence (events/PPY)** | **Incidence (events/PPY)** | **Incidence**  **(events/PPY)** | **Incidence Rate Ratio (95% CI)*** | **p-value*** | **Incidence (events/PPY)** | **Incidence Rate Ratio**  **(95% CI)*** | **p-value*** |  |
| **Painful events** |  |  |  |  |  |  |  |  |  |
| **Age at enrollment** |  |  |  |  |  |  |  |  |  |
| 1- 5 years | 4.10  (3.38 - 4.97) | 4.48  (3.41 - 5.90) | 3.59  (2.64 - 4.89) | 0.80  (0.48 - 1.34) | 0.68 | 4.17  (2.70 - 6.45) | 0.93  (0.48 - 1.80) | >0.99 |  |
| 5 – 10 years | 4.45  (3.70 - 5.35) | 4.34  (3.26 - 5.79) | 4.86  (3.38 - 7.00) | 1.12  (0.64 - 1.97) | 0.97 | 4.18  (3.09 - 5.66) | 0.96  (0.56 - 1.65) | >0.99 |  |
| Interaction p-value | 0.50 |  |  |  |  |  |  |  |  |
| **Hydroxyurea use at baseline** |  |  |  |  |  |  |  |  |  |
| Yes | 3.73  (3.05 - 4.57) | 4.20  (3.23 - 5.47) | 3.39  (2.14 - 5.35) | 0.81  (0.42 - 1.54) | 0.84 | 3.29  (2.23 - 4.85) | 0.78  (0.43 - 1.43) | 0.72 |  |
| No | 4.79  (4.02 - 5.71) | 4.66  (3.45 - 6.30) | 4.87  (3.68 - 6.44) | 1.04  (0.62 - 1.75) | >0.99 | 4.85  (3.42 - 6.86) | 1.04  (0.58 - 1.88) | >0.99 |  |
| Interaction p-value | 0.58 |  |  |  |  |  |  |  |  |
| **Sex** |  |  |  |  |  |  |  |  |  |
| Girls | 4.14  (3.34 - 5.11) | 4.23  (3.06 - 5.86) | 4.25  (2.86 - 6.32) | 1.00  (0.54 - 1.88) | >0.99 | 3.90  (2.61 - 5.82) | 0.92  (0.47 - 1.79) | 0.99 |  |
| Boys | 4.44  (3.81 - 5.17) | 4.58  (3.61 - 5.82) | 4.12  (3.31 - 5.14) | 0.90  (0.59 - 1.36) | 0.93 | 4.54  (3.21 - 6.42) | 0.99  (0.58 - 1.70) | >0.99 |  |
| Interaction p-value | 0.86 |  |  |  |  |  |  |  |  |
| **Hospitalized in prior 12 months** |  |  |  |  |  |  |  |  |  |
| Yes | 4.37  (3.55 - 5.38) | 4.85  (3.68 - 6.38) | 4.03  (2.69 - 6.04) | 0.83  (0.46 - 1.52) | 0.87 | 4.17  (2.70 - 6.43) | 0.86  (0.44 - 1.66) | 0.95 |  |
| No | 4.19  (3.53 - 4.97) | 4.08  (3.06 - 5.43) | 4.35  (3.22 - 5.87) | 1.07  (0.64 - 1.78) | 0.99 | 4.19  (3.08 - 5.69) | 1.03  (0.60 - 1.76) | >0.99 |  |
| Interaction p-value | 0.69 |  |  |  |  |  |  |  |  |
| **Baseline hemoglobin (g/dL)** |  |  |  |  |  |  |  |  |  |
| < 7 | 4.30  (3.09 - 5.99) | 4.63  (2.95 - 7.26) | 5.36  (2.83 - 10.16) | 1.16  (0.41 - 3.28) | >0.99 | 2.80  (1.63 - 4.82) | 0.60  (0.23 - 1.60) | 0.58 |  |
| 7 – 9 | 4.27  (3.65 - 5.00) | 4.45  (3.44 - 5.76) | 4.34  (3.32 - 5.68) | 0.98  (0.60 - 1.59) | >0.99 | 3.93  (2.90 - 5.34) | 0.88  (0.51 - 1.53) | 0.98 |  |
| > 9 | 4.91  (3.33 - 7.25) | 4.20  (2.76 - 6.40) | 3.02  (2.19 - 4.17) | 0.72  (0.35 - 1.50) | 0.72 | 6.89  (3.50 - 13.58) | 1.64  (0.55 - 4.93) | 0.71 |  |
| Interaction p-value | 0.12 |  |  |  |  |  |  |  |  |
| **Dactylitis** |  |  |  |  |  |  |  |  |  |
| **Age at enrollment** |  |  |  |  |  |  |  |  |  |
| 1- 5 years | 1.09  (0.73 - 1.62) | 1.50  (0.90 - 2.50) | 1.03  (0.52 - 2.07) | 0.69  (0.29 - 1.65) | 0.69 | 0.67  (0.41 - 1.09) | 0.45  (0.18 - 1.11) | 0.093 |  |
| 5 – 10 years | 0.60  (0.35 - 1.03) | 0.81  (0.34 - 1.89) | 0.56  (0.24 - 1.32) | 0.70  (0.15 - 3.28) | 0.95 | 0.33  (0.15 - 0.74) | 0.41  (0.09 - 1.86) | 0.40 |  |
| Interaction p-value | 0.99 |  |  |  |  |  |  |  |  |
| **Hydroxyurea use at baseline** |  |  |  |  |  |  |  |  |  |
| Yes | 0.86  (0.54 - 1.37) | 1.15  (0.58 - 2.29) | 0.78  (0.37 - 1.63) | 0.68  (0.19 - 2.39) | 0.87 | 0.42  (0.23 - 0.79) | 0.37  (0.11 - 1.23) | 0.13 |  |
| No | 0.83  (0.53 - 1.29) | 1.08  (0.62 - 1.88) | 0.83  (0.38 - 1.81) | 0.76  (0.32 - 1.82) | 0.87 | 0.56  (0.32 - 0.98) | 0.52  (0.19 - 1.42) | 0.31 |  |
| Interaction p-value | 0.85 |  |  |  |  |  |  |  |  |
| **Sex** |  |  |  |  |  |  |  |  |  |
| Girls | 0.87  (0.58 - 1.31) | 1.23  (0.61 - 2.46) | 0.83  (0.45 - 1.50) | 0.67  (0.22 - 2.10) | 0.81 | 0.50  (0.29 - 0.86) | 0.40  (0.13 - 1.26) | 0.15 |  |
| Boys | 0.81  (0.48 - 1.36) | 1.01  (0.57 - 1.77) | 0.77  (0.27 - 2.18) | 0.77  (0.25 - 2.36) | 0.94 | 0.52  (0.27 - 1.02) | 0.52  (0.17 - 1.60) | 0.41 |  |
| Interaction p-value | 0.91 |  |  |  |  |  |  |  |  |
| **Hospitalized in prior 12 months** |  |  |  |  |  |  |  |  |  |
| Yes | 0.78  (0.52 - 1.15) | 1.10  (0.59 - 2.03) | 0.72  (0.31 - 1.65) | 0.65  (0.17 - 2.54) | 0.86 | 0.48  (0.27 - 0.85) | 0.44  (0.15 - 1.28) | 0.18 |  |
| No | 0.91  (0.56 - 1.47) | 1.14  (0.59 - 2.20) | 0.89  (0.43 - 1.82) | 0.78  (0.30 - 2.06) | 0.93 | 0.54  (0.28 - 1.02) | 0.47  (0.14 - 1.53) | 0.33 |  |
| Interaction p-value | 0.96 |  |  |  |  |  |  |  |  |
| **Baseline hemoglobin (g/dL)** |  |  |  |  |  |  |  |  |  |
| < 7 | 1.03  (0.41 - 2.63) | 0.95  (0.27 - 3.37) | 1.42  (0.43 - 4.68) | 1.49  (0.50 - 4.47) | 0.86 | 0.64  (0.22 - 1.87) | 0.67  (0.07 - 6.53) | >0.99 |  |
| 7 – 9 | 0.79  (0.55 - 1.14) | 1.12  (0.62 - 2.02) | 0.69  (0.38 - 1.26) | 0.62  (0.19 - 2.00) | 0.79 | 0.42  (0.25 - 0.69) | 0.37  (0.13 - 1.08) | 0.079 |  |
| > 9 | 1.02  (0.49 - 2.11) | 1.31  (0.40 - 4.26) | 1.09  (0.39 - 3.07) | 0.83  (0.16 - 4.35) | >0.99 | 0.70  (0.25 - 1.96) | 0.54  (0.06 - 4.56) | 0.94 |  |
| Interaction p-value | 0.63 |  |  |  |  |  |  |  |  |

* Relative to Daily Proguanil.

SP-AQ: Sulfadoxine/Pyrimethamine-Amodiaquine; DP: Dihydroartemisinin/Piperaquine; CI: confidence interval; PPY: per person-year, g/dL: grams per deciliter

## Table E. Serious Adverse Events in the Intention-To-Treat population

| **Adverse Event** | **Overall (N=246)** | **Daily Proguanil (N=81)** | **Monthly SP-AQ (N=83)** | **Monthly DP (N=82)** |
| --- | --- | --- | --- | --- |
| **Painful crisis** |  |  |  |  |
| Number of occurrences | 91 | 33 | 28 | 30 |
| Number of patients (%) | 63 (25.6%) | 22 (27.2%) | 22 (26.5%) | 19 (23.2%) |
| **Anemia** |  |  |  |  |
| Number of occurrences | 33 | 12 | 14 | 7 |
| Number of patients (%) | 29 (11.8%) | 10 (12.3%) | 12 (14.5%) | 7 (8.5%) |
| **Malaria** |  |  |  |  |
| Number of occurrences | 12 | 4 | 3 | 5 |
| Number of patients (%) | 11 (4.5%) | 4 (4.9%) | 3 (3.6%) | 4 (4.9%) |
| **Sepsis** |  |  |  |  |
| Number of occurrences | 12 | 4 | 5 | 3 |
| Number of patients (%) | 12 (4.9%) | 4 (4.9%) | 5 (6.0%) | 3 (3.7%) |
| **Elevated ALT** |  |  |  |  |
| Number of occurrences | 10 | 4 | 4 | 2 |
| Number of patients (%) | 9 (3.7%) | 3 (3.7%) | 4 (4.8%) | 2 (2.4%) |
| **Pneumonia** |  |  |  |  |
| Number of occurrences | 8 | 4 | 2 | 2 |
| Number of patients (%) | 8 (3.3%) | 4 (4.9%) | 2 (2.4%) | 2 (2.4%) |
| **Acute chest syndrome** |  |  |  |  |
| Number of occurrences | 7 | 2 | 5 | 0 |
| Number of patients (%) | 6 (2.4%) | 2 (2.5%) | 4 (4.8%) | 0 |
| **Adenotonsillar hypertrophy** |  |  |  |  |
| Number of occurrences | 2 | 0 | 1 | 1 |
| Number of patients (%) | 2 (0.8%) | 0 | 1 (1.2%) | 1 (1.2%) |
| **Thrombocytopenia** |  |  |  |  |
| Number of occurrences | 2 | 0 | 1 | 1 |
| Number of patients (%) | 2 (0.8%) | 0 | 1 (1.2%) | 1 (1.2%) |
| **Elevated white blood cells** |  |  |  |  |
| Number of occurrences | 1 | 1 | 0 | 0 |
| Number of patients (%) | 1 (0.4%) | 1 (1.2%) | 0 | 0 |
| **Hemolytic crisis** |  |  |  |  |
| Number of occurrences | 1 | 0 | 1 | 0 |
| Number of patients (%) | 1 (0.4%) | 0 | 1 (1.2%) | 0 |
| **Low platelet count** |  |  |  |  |
| Number of occurrences | 1 | 0 | 1 | 0 |
| Number of patients (%) | 1 (0.4%) | 0 | 1 (1.2%) | 0 |
| **Lumbago** |  |  |  |  |
| Number of occurrences | 1 | 0 | 0 | 1 |
| Number of patients (%) | 1 (0.4%) | 0 | 0 | 1 (1.2%) |
| **Sickle cell disease** |  |  |  |  |
| Number of occurrences | 1 | 0 | 1 | 0 |
| Number of patients (%) | 1 (0.4%) | 0 | 1 (1.2%) | 0 |
| **Soft tissue injury** |  |  |  |  |
| Number of occurrences | 1 | 0 | 0 | 1 |
| Number of patients (%) | 1 (0.4%) | 0 | 0 | 1 (1.2%) |
| **Unknown** |  |  |  |  |
| Number of occurrences | 1 | 0 | 0 | 1 |
| Number of patients (%) | 1 (0.4%) | 0 | 0 | 1 (1.2%) |

SP-AQ: Sulfadoxine/Pyrimethamine-Amodiaquine; DP: Dihydroartemisinin/Piperaquine; ALT: alanine aminotransferase

## Table F. Change from Baseline in Neutrophil Count (10^9^/L) in the Intention-to-Treat Population

|  | **Overall (N=246)** | | **Daily Proguanil (N=81)** | | **Monthly SP-AQ (N=83)** | | **Monthly DP (N=82)** | |  |
| --- | --- | --- | --- | --- | --- | --- | --- | --- | --- |
| **Time** | **Value** | **Change from baseline** | **Value** | **Change from baseline** | **Value** | **Change from baseline** | **Value** | **Change from baseline** |  |
| **Baseline** |  |  |  |  |  |  |  |  |  |
| N | 238 |  | 80 |  | 78 |  | 80 |  |  |
| Mean (SD) | 5.63 (3.92) |  | 5.34 (2.86) |  | 5.80 (3.07) |  | 5.76 (5.36) |  |  |
| Median (IQR) | 4.93 (3.46, 6.93) |  | 4.82 (3.25, 6.92) |  | 5.64 (3.51, 7.24) |  | 4.77 (3.57, 6.55) |  |  |
| Range (min, max) | 0.42, 47.30 |  | 1.40, 18.64 |  | 0.42, 14.21 |  | 1.42, 47.30 |  |  |
|  | | | | | | | | |  |
| **Month 1^[2]^** |  |  |  |  |  |  |  |  |  |
| N | 7 | 0 | 1 | 0 | 4 | 0 | 2 | 0 |  |
| Mean (SD) | 6.38 (3.88) | - (-) | 7.82 (-) | - (-) | 6.82 (4.91) | - (-) | 4.79 (3.18) | - (-) |  |
| Median (IQR) | 6.13 (2.54, 7.82) | - (-, -) | 7.82 (7.82, 7.82) | - (-, -) | 5.85 (3.76, 9.89) | - (-, -) | 4.79 (2.54, 7.04) | - (-, -) |  |
| Range (min, max) | 1.96, 13.64 | -, - | 7.82, 7.82 | -, - | 1.96, 13.64 | -, - | 2.54, 7.04 | -, - |  |
|  | | | | | | | | |  |
| **Month 3** |  |  |  |  |  |  |  |  |  |
| N | 232 | 225 | 79 | 78 | 75 | 71 | 78 | 76 |  |
| Mean (SD) | 5.32 (3.05) | -0.37 (4.62) | 5.44 (3.75) | 0.09 (4.26) | 5.63 (2.78) | -0.33 (3.24) | 4.91 (2.45) | -0.87 (5.89) |  |
| Median (IQR) | 5.01 (3.29, 6.61) | -0.14 (-1.95, 1.34) | 5.14 (3.25, 6.24) | -0.13 (-1.95, 1.62) | 5.18 (3.80, 6.95) | 0.34 (-2.00, 1.34) | 4.59 (2.90, 6.29) | -0.48 (-1.96, 1.18) |  |
| Range (min, max) | 0.81, 30.10 | -44.21, 25.51 | 1.07, 30.10 | -11.94, 25.51 | 1.21, 13.81 | -11.31, 6.70 | 0.81, 12.79 | -44.21, 9.52 |  |
|  | | | | | | | | |  |
| **Month 4** |  |  |  |  |  |  |  |  |  |
| N | 1 | 1 | 0 | 0 | 1 | 1 | 0 | 0 |  |
| Mean (SD) | 4.16 (-) | 0.02 (-) | - (-) | - (-) | 4.16 (-) | 0.02 (-) | - (-) | - (-) |  |
| Median (IQR) | 4.16 (4.16, 4.16) | 0.02 (0.02, 0.02) | - (-, -) | - (-, -) | 4.16 (4.16, 4.16) | 0.02 (0.02, 0.02) | - (-, -) | - (-, -) |  |
| Range (min, max) | 4.16, 4.16 | 0.02, 0.02 | -, - | -, - | 4.16, 4.16 | 0.02, 0.02 | -, - | -, - |  |
|  | | | | | | | | |  |
| **Month 6** |  |  |  |  |  |  |  |  |  |
| N | 222 | 216 | 77 | 76 | 70 | 67 | 75 | 73 |  |
| Mean (SD) | 5.17 (2.81) | -0.39 (4.36) | 5.02 (2.77) | -0.03 (3.21) | 5.42 (2.70) | -0.45 (3.34) | 5.08 (2.96) | -0.71 (5.97) |  |
| Median (IQR) | 4.64 (3.33, 6.33) | -0.23 (-1.86, 1.05) | 4.54 (3.53, 5.78) | -0.05 (-1.78, 1.10) | 4.79 (3.55, 7.03) | -0.24 (-2.36, 0.76) | 4.63 (2.94, 6.47) | -0.28 (-1.67, 1.05) |  |
| Range (min, max) | 0.46, 19.93 | -43.59, 16.36 | 1.34, 19.93 | -8.86, 12.26 | 1.23, 15.73 | -11.09, 10.13 | 0.46, 18.85 | -43.59, 16.36 |  |
|  | | | | | | | | |  |
| **Month 7** |  |  |  |  |  |  |  |  |  |
| N | 2 | 1 | 0 | 0 | 2 | 1 | 0 | 0 |  |
| Mean (SD) | 5.24 (1.25) | 1.93 (-) | - (-) | - (-) | 5.24 (1.25) | 1.93 (-) | - (-) | - (-) |  |
| Median (IQR) | 5.24 (4.35, 6.12) | 1.93 (1.93, 1.93) | - (-, -) | - (-, -) | 5.24 (4.35, 6.12) | 1.93 (1.93, 1.93) | - (-, -) | - (-, -) |  |
| Range (min, max) | 4.35, 6.12 | 1.93, 1.93 | -, - | -, - | 4.35, 6.12 | 1.93, 1.93 | -, - | -, - |  |
|  | | | | | | | | |  |
| **Month 9** |  |  |  |  |  |  |  |  |  |
| N | 217 | 210 | 75 | 74 | 71 | 67 | 71 | 69 |  |
| Mean (SD) | 5.39 (3.37) | -0.14 (4.82) | 5.28 (2.57) | 0.07 (3.12) | 5.66 (3.72) | -0.16 (3.99) | 5.25 (3.74) | -0.36 (6.74) |  |
| Median (IQR) | 4.74 (3.31, 6.58) | 0.09 (-1.81, 1.50) | 4.84 (3.41, 6.66) | 0.37 (-1.63, 1.39) | 4.65 (3.64, 6.58) | 0.02 (-2.03, 1.55) | 4.90 (2.72, 6.67) | 0.00 (-1.86, 1.57) |  |
| Range (min, max) | 0.58, 28.61 | -46.72, 20.67 | 1.20, 12.88 | -10.05, 10.15 | 1.32, 22.29 | -11.00, 15.00 | 0.58, 28.61 | -46.72, 20.67 |  |
|  | | | | | | | | |  |
| **Month 12** |  |  |  |  |  |  |  |  |  |
| N | 212 | 206 | 74 | 73 | 67 | 63 | 71 | 70 |  |
| Mean (SD) | 5.29 (2.98) | -0.18 (4.69) | 5.97 (3.89) | 0.79 (4.12) | 5.24 (2.16) | -0.49 (3.41) | 4.63 (2.38) | -0.90 (5.96) |  |
| Median (IQR) | 4.83 (3.38, 6.60) | -0.14 (-1.66, 1.54) | 5.26 (3.56, 7.24) | 0.24 (-1.38, 2.01) | 4.96 (3.85, 6.48) | -0.14 (-1.98, 1.53) | 4.21 (2.67, 5.73) | -0.31 (-1.74, 1.09) |  |
| Range (min, max) | 1.03, 25.66 | -45.16, 23.01 | 1.03, 25.66 | -9.30, 23.01 | 1.21, 11.60 | -10.31, 8.01 | 1.46, 12.38 | -45.16, 5.76 |  |
|  | | | | | | | | |  |
| SD: standard deviation; IQR: interquartile range; -: not applicable  ^[1]^ Baseline measures are taken at either screening or enrollment. | | | | | | | | | |
| ^[2]^ CBC was repeated at Month 1 for 7 patients whose specimen drawn at enrollment was rejected. | | | | | | | | | |

## Table G. Change from Baseline in Platelet Count (10^9^/L) in the Intention-to-Treat Population

|  | **Overall (N=246)** | | **Daily Proguanil (N=81)** | | **Monthly SP-AQ (N=83)** | | **Monthly DP (N=82)** | |  |
| --- | --- | --- | --- | --- | --- | --- | --- | --- | --- |
| **Time** | **Value** | **Change from baseline** | **Value** | **Change from baseline** | **Value** | **Change from baseline** | **Value** | **Change from baseline** |  |
| **Baseline** |  |  |  |  |  |  |  |  |  |
| N | 238 |  | 80 |  | 78 |  | 80 |  |  |
| Mean (SD) | 466 (185) |  | 455 (179) |  | 472 (185) |  | 470 (194) |  |  |
| Median (IQR) | 464 (344, 585) |  | 441 (362, 586) |  | 487 (352, 595) |  | 471 (330, 580) |  |  |
| Range (min, max) | 30, 1076 |  | 30, 833 |  | 32, 1007 |  | 116, 1076 |  |  |
|  | | | | | | | | |  |
| **Month 1^[2]^** |  |  |  |  |  |  |  |  |  |
| N | 7 | 0 | 1 | 0 | 4 | 0 | 2 | 0 |  |
| Mean (SD) | 564 (162) | - (-) | 737 (-) | - (-) | 543 (149) | - (-) | 520 (233) | - (-) |  |
| Median (IQR) | 563 (355, 708) | - (-, -) | 737 (737, 737) | - (-, -) | 559 (450, 636) | - (-, -) | 520 (355, 685) | - (-, -) |  |
| Range (min, max) | 345, 737 | -, - | 737, 737 | -, - | 345, 708 | -, - | 355, 685 | -, - |  |
|  | | | | | | | | |  |
| **Month 3** |  |  |  |  |  |  |  |  |  |
| N | 232 | 225 | 79 | 78 | 75 | 71 | 78 | 76 |  |
| Mean (SD) | 461 (181) | -6 (203) | 454 (171) | -6 (201) | 456 (200) | -26 (220) | 472 (174) | 14 (190) |  |
| Median (IQR) | 460 (356, 563) | 3 (-114, 106) | 459 (353, 567) | 7 (-93, 123) | 452 (356, 544) | -22 (-148, 77) | 482 (371, 572) | 11 (-78, 124) |  |
| Range (min, max) | 42, 1520 | -623, 762 | 42, 795 | -462, 391 | 48, 1520 | -499, 762 | 46, 976 | -623, 540 |  |
|  | | | | | | | | |  |
| **Month 4** |  |  |  |  |  |  |  |  |  |
| N | 1 | 1 | 0 | 0 | 1 | 1 | 0 | 0 |  |
| Mean (SD) | 284 (-) | -194 (-) | - (-) | - (-) | 284 (-) | -194 (-) | - (-) | - (-) |  |
| Median (IQR) | 284 (284, 284) | -194 (-194, -194) | - (-, -) | - (-, -) | 284 (284, 284) | -194 (-194, -194) | - (-, -) | - (-, -) |  |
| Range (min, max) | 284, 284 | -194, -194 | -, - | -, - | 284, 284 | -194, -194 | -, - | -, - |  |
|  | | | | | | | | |  |
| **Month 6** |  |  |  |  |  |  |  |  |  |
| N | 223 | 217 | 78 | 77 | 70 | 67 | 75 | 73 |  |
| Mean (SD) | 456 (156) | -5 (201) | 458 (147) | 4 (196) | 452 (175) | -19 (190) | 457 (149) | -4 (217) |  |
| Median (IQR) | 446 (357, 562) | -4 (-110, 123) | 440 (367, 562) | -13 (-104, 106) | 458 (359, 562) | 5 (-127, 109) | 437 (349, 570) | -4 (-126, 137) |  |
| Range (min, max) | 33, 901 | -769, 547 | 33, 892 | -485, 371 | 45, 823 | -507, 487 | 126, 901 | -769, 547 |  |
|  | | | | | | | | |  |
| **Month 7** |  |  |  |  |  |  |  |  |  |
| N | 2 | 1 | 0 | 0 | 2 | 1 | 0 | 0 |  |
| Mean (SD) | 572 (38) | 140 (-) | - (-) | - (-) | 572 (38) | 140 (-) | - (-) | - (-) |  |
| Median (IQR) | 572 (545, 599) | 140 (140, 140) | - (-, -) | - (-, -) | 572 (545, 599) | 140 (140, 140) | - (-, -) | - (-, -) |  |
| Range (min, max) | 545, 599 | 140, 140 | -, - | -, - | 545, 599 | 140, 140 | -, - | -, - |  |
|  | | | | | | | | |  |
| **Month 9** |  |  |  |  |  |  |  |  |  |
| N | 218 | 211 | 75 | 74 | 71 | 67 | 72 | 70 |  |
| Mean (SD) | 472 (163) | 7 (195) | 462 (157) | 8 (203) | 478 (153) | 1 (196) | 477 (181) | 12 (189) |  |
| Median (IQR) | 484 (359, 574) | 22 (-110, 130) | 458 (350, 551) | 14 (-104, 163) | 492 (367, 577) | 2 (-118, 102) | 494 (346, 588) | 30 (-80, 136) |  |
| Range (min, max) | 6, 949 | -643, 572 | 134, 944 | -480, 446 | 104, 949 | -643, 572 | 6, 876 | -484, 504 |  |
|  | | | | | | | | |  |
| **Month 12** |  |  |  |  |  |  |  |  |  |
| N | 213 | 207 | 75 | 74 | 67 | 63 | 71 | 70 |  |
| Mean (SD) | 475 (173) | 11 (213) | 478 (178) | 24 (209) | 465 (164) | -11 (223) | 482 (179) | 16 (209) |  |
| Median (IQR) | 460 (380, 573) | 27 (-122, 128) | 475 (377, 610) | 39 (-98, 173) | 452 (386, 564) | 30 (-177, 122) | 454 (376, 559) | 22 (-102, 105) |  |
| Range (min, max) | 4, 1212 | -603, 776 | 4, 868 | -528, 440 | 92, 970 | -443, 616 | 103, 1212 | -603, 776 |  |
|  | | | | | | | | |  |
| SD: standard deviation; IQR: interquartile range; -: not applicable  ^[1]^ Baseline measures are taken at either screening or enrollment. | | | | | | | | | |
| ^[2]^ CBC was repeated at Month 1 for 7 patients whose specimen drawn at enrollment was rejected. | | | | | | | | | |

## Table H. G6PD and CYP2C8*2 Alleles in 10 Participants Who Died

| **Sex** | **Arm** | **Interval on study, days** | **Approximate age at enrollment, y** | **SAE term(s)** | **G6PD allele** | **CYP2C8*2 allele** |
| --- | --- | --- | --- | --- | --- | --- |
| Girl | Proguanil | 110 | 2.5 | Painful crisis | B | Wildtype |
| Girl | Proguanil | 279 | 4 | Severe anemia  Painful crisis | B | Heterozygote |
| Girl | SP-AQ | 81 | 2.5 | Severe anemia | B | Wildtype |
| Boy | SP-AQ | 102 | 3.5 | Hemolytic crisis | B | Wildtype |
| Boy | SP-AQ | 148 | 10 | Painful crisis | A+ | Wildtype |
| Boy | SP-AQ | 186 | 7 | Severe anemia  Acute chest syndrome | A- | Wildtype |
| Girl | SP-AQ | 258 | 4 | Pneumonia  Malaria | A+/B | Heterozygote |
| Boy | SP-AQ | 267 | 2 | Acute chest syndrome | B | Wildtype |
| Boy | SP-AQ | 294 | 4 | Painful crisis | A- | Wildtype |
| Boy | DP | 279 | 3 | Severe anemia | B | Wildtype |

SP-AQ: Sulfadoxine/Pyrimethamine-Amodiaquine; DP: Dihydroartemisinin/Piperaquine; SAE: serious adverse event; G6PD: glucose-6-phosphate dehydrogenase

## Table I. QTcF Measurements in DP Recipients and Change from Baseline

|  |  |  |  |  | **Change from baseline** | | |
| --- | --- | --- | --- | --- | --- | --- | --- |
| **Timepoint** | **No.** | **Mean (SD)** | **Range** | **>450 msec, n** | **Mean (SD)** | **Range** | **>50 msec, n** |
| **Baseline*** | 12 | 428 (16) | 397, 448 | 0 | NA | NA | NA |
| **Enrollment** | 12 | 429 (27) | 352, 450 | 0 | 1 (33) | -83, 47 | 0 |
| **Month 1** | 12 | 434 (13) | 404, 457 | 1 | 6 (20) | -44, 35 | 0 |
| **2** | 11 | 433 (16) | 402, 448 | 0 | 4 (19) | -35, 30 | 0 |
| **3** | 12 | 441 (17) | 413, 473 | 2 | 13 (20) | -23, 48 | 0 |
| **4** | 11 | 443 (10) | 419, 459 | 1 | 15 (14) | -2, 41 | 0 |
| **5** | 12 | 440 (15) | 412, 466 | 3 | 12 (13) | -12, 34 | 0 |
| **6** | 11 | 446 (16) | 419, 479 | 2 | 17 (13) | 2, 43 | 0 |
| **7** | 12 | 441 (11) | 420, 456 | 2 | 13 (13) | -4, 39 | 0 |
| **8** | 10 | 437 (13) | 419, 453 | 1 | 10 (23) | -28, 52 | 1 |
| **9** | 12 | 444 (13) | 421, 469 | 3 | 16 (14) | 1, 45 | 0 |
| **10** | 10 | 435 (13) | 411, 449 | 0 | 7 (20) | -36, 38 | 0 |
| **11** | 9 | 434 (14) | 415, 451 | 1 | 8 (16) | -13, 40 | 0 |

* Only among children enrolled in the ECG sub-study

QTcF: QT interval corrected for heart rate using Fridericia’s method; DP: Dihydroartemisinin/Piperaquine; SD: standard deviation; msec: milliseconds

# References

1. World Health Organization, Communicable Diseases Cluster. Severe falciparum malaria. Trans R Soc Trop Med Hyg. 2000;94 Suppl 1:S1-90. Epub 2000/12/05. PubMed PMID: 11103309

2. Thornburg CD, Files BA, Luo Z, Miller ST, Kalpatthi R, Iyer R, et al. Impact of hydroxyurea on clinical events in the BABY HUG trial. Blood. 2012;120(22):4304-10; quiz 448. Epub 2012/08/24. doi: 10.1182/blood-2012-03-419879. PubMed PMID: 22915643; PubMed Central PMCID: PMC3507142.

3. Ministry of Public Health and Sanitation, Republic of Kenya. National Guidelines for the Diagnosis, Treatment, and Prevention of Malaria in Kenya. 2010.

4. World Health Organization. WHO Policy Recommendation: Seasonal Malaria Chemoprevention (SMC) for *Plasmodium falciparum* malaria control in highly seasonal transmission areas of the Sahel sub-region in Africa. 2012. https://apps.who.int/iris/handle/10665/337978 (Accessed August 23, 2022)

5. Marwa KJ, Schmidt T, Sjogren M, Minzi OM, Kamugisha E, Swedberg G. Cytochrome P450 single nucleotide polymorphisms in an indigenous Tanzanian population: a concern about the metabolism of artemisinin-based combinations. Malaria J. 2014;13:420. Epub 2014/11/05. doi: 10.1186/1475-2875-13-420. PubMed PMID: 25363545; PubMed Central PMCID: PMCPMC4228099.

6. Hirono A, Beutler E. Molecular cloning and nucleotide sequence of cDNA for human glucose-6-phosphate dehydrogenase variant A(-). Proc Nat Acad Sci U S A. 1988;85(11):3951-4. Epub 1988/06/01. doi: 10.1073/pnas.85.11.3951. PubMed PMID: 2836867; PubMed Central PMCID: PMCPMC280338.
