# Supplementary material for: Monthly sulfadoxine/pyrimethamine-amodiaquine or dihydroartemisinin-piperaquine as malaria chemoprevention in young Kenyan children with sickle cell anemia: A randomized controlled trial
Source: PLoS Med. 2022 Oct 10;19(10):e1004104. doi: 10.1371/journal.pmed.1004104 (PMC9591057; doi:10.1371/journal.pmed.1004104)
Supplement: Attachment — Submitted filename: EPiTOMISE PlosMed RevResponse final.docx [file pmed.1004104.s005.docx]

17 May 2022

Richard Turner PhD

Senior Editor, *Plos Medicine*

Dr. Turner:

This letter accompanies the resubmission of our manuscript entitled “Monthly sulfadoxine/pyrimethamine-amodiaquine or dihydroartemisinin-piperaquine as malaria chemoprevention in young Kenyan children with sickle cell anemia: A randomized controlled trial.” We appreciate the opportunity to revise the manuscript and believe it has been enhanced in doing so.

As with the initial submission, we have not submitted this manuscript for consideration to publish elsewhere and my co-authors Sarah Korwa, Angie Wu, Cynthia Green, Betsy Freedman, Sheila Clapp, Joseph Kipkoech Kirui, Wendy O’Meara, and Festus Njuguna all meet criteria for authorship and agree with this submission.

Below we have enumerated our point-by-point responses to the reviewer comments. For each comment, the reviewer comments are italicized, and our responses are indented and in normal text, with specific text changes underlined.

**Editorial Requests**

1. *Please adapt the data statement (submission form) as appropriate, noting that authors cannot be listed as contacts for inquiries about data access according to PLOS' policy.*

RESPONSE: We are still investigating this. We have not identified an entity at Duke, the Duke Clinical Research Institute, or Moi University that is qualified to serve as a durable point of contact. We are open to suggestions beyond those that are included in your policy (ethics committee, or data access committee). We will continue to investigate while this review is under consideration.

2. *Please quote the country name in your title.*

RESPONSE: Changed title to “Monthly sulfadoxine/pyrimethamine-amodiaquine or dihydroartemisinin-piperaquine as malaria chemoprevention in young Kenyan children with sickle cell anemia: A randomized controlled trial”

3. *Please quote the study dates in your abstract.*

RESPONSE: Added to the Abstract: “… open-label trial conducted between January 23, 2018, and December 15, 2020.”

4. *Please quote participant demographic details in the abstract.*

RESPONSE: Added to the Abstract: “Overall, 53.3% (n=131) were boys and the mean age was 4.6 ± 2.5 years.”

5. *Noting "p<0.01" at line 54 (abstract), please quote exact p values or "p<0.001" throughout.*

RESPONSE: We have confirmed that all p-values have been reported using 3-digits to the right of the decimal for any p <0.10 within the manuscript text and tables.

6. *Please add a new final sentence to the "Methods and findings" subsection of your abstract, which should begin "Study limitations include ..." or similar and should quote 2-3 of the study's main limitations.*

RESPONSE: Added to Abstract: “Study limitations include the unexpectedly limited transmission of *P. falciparum* in the study setting, the high use of hydroxyurea, and the enhanced supportive care for trial participants, which may limit generalizability to higher-transmission settings where routine sickle-cell care is more limited.”

7. *After the abstract, please add a new and accessible "Author summary" section in non-identical prose. You may find it helpful to consult one or two recent research papers in PLOS Medicine to get a sense of the preferred style.*

RESPONSE: We have added this section to the text.

8. *Throughout the main text, please style reference call-outs as follows: " ... complications [6,7]." (noting the absence of spaces within the square brackets).*

RESPONSE: We have changed this.

9. *Noting reference 1 and any others, please use the journal name abbreviation "PLoS Med."; and "PLoS ONE" where appropriate.*

RESPONSE: We fixed these.

10. Noting references 8 & 13, we suggest spelling out the institution names.

RESPONSE: We’ve fixed these.

11. Noting reference 14 and others, please list 6 author names where appropriate, followed by "et al.".

RESPONSE: We’ve fixed this too.

12. Noting reference 17, please list the individual or institutional author first.

RESPONSE: This has always been a weird reference, but we’ve tried to clarify it.

13. Please add an author name to reference 23.

RESPONSE: Changed.

14. Please add a completed CONSORT checklist, labelled "S1_CONSORT_Checklist" or similar and referred to as such in the Methods section (main text).

RESPONSE: We’ve included a CONSORT checklist and added a call-out in the text. In the Methods first subsection.

15. In the checklist, please refer to individual items by section (e.g., "Methods") and paragraph number, not by page or line numbers as these generally change in the event of publication.

RESPONSE: See #14 above.

**Academic Editor comments**

16. *The impact of the 3 drug regimens on asymptomatic infections should be included in the abstract.*

RESPONSE: We have added to the Abstract: “…The incidence of *P. falciparum* infection relative to daily Proguanil was similar in the monthly SP-AQ group (IRR 0.46; 95% CI: 0.17—1.20; p=0.13) but reduced with monthly DP (IRR 0.21; 95% CI: 0.08—0.56; p<0.01).”

17. *The authors could also create an additional endpoint by combining asymptomatic and symptomatic Pf infections to increase the power.*

RESPONSE: This endpoint was not considered as a secondary or exploratory endpoint given that we may possible “double count” some episodes, as a symptomatic event may have occurred within days after an asymptomatic event. Therefore, some events could be part of the same episode. Additionally, given that only 9 symptomatic cases were observed, this would not provide additional insight.

18. *There is an intriguing trend suggesting that SP-AQ is more beneficial than proguanil but less beneficial than DP. The authors conclude that there is no role for SP-AQ and DP is the solution. This may be jumping to conclusions as Reviewer 2 points out.*

RESPONSE: We have changed the Discussion to read: “On balance of efficacy and safety, monthly SP-AQ does not appear to have a role…” and “Reductions in asymptomatic *P. falciparum* infection and dactylitis did not reach statistical significance, and while these were undermined by low malaria transmission and likely by prevalent resistance to SP in Kenya,[26] rates of general outcomes did not suggest benefit of the putative antibacterial activity of the sulfadoxine component.”

19. *SP-AQ has by now been used in 10s of millions of children on seasonal chemoprophylaxis we know it is safe, available and accepted. DP may be ideal, but policymakers may be reluctant to roll out DP for prophylaxis. The experience in Cambodia shows that DP -resistance is a real danger. If DP no longer works, there are few options left. This threat of emerging and spreading resistance should be discussed by authors.*

RESPONSE: We have added to the Discussion: “These clinical factors would need to be complemented before wider policy adoption by considerations of DP resistance, which was detected as declining clinical efficacy first in Southeast Asia [Saunders DL Nejm 2014] and more recently at several sites in Africa [Moriarty Ajtmh 2021, Gansane Mal J 2021]. Though wider use of DP will necessarily increase pressure to develop resistance, this may be mitigated by a restriction of DP use to high-risk populations like pregnant women and children with SCA, and by the observation through multiple studies [26-29] that mass-drug administration with DP across diverse populations has not reliably increased molecular markers of DP resistance.”

20. *The excess mortality in the SP-AQ arm is not statistically significant this is most probably due to chance. The authors have to be careful not to mislead the reader.*

RESPONSE: Indeed, similar to the “trends” noted above for efficacy of SP-AQ, this is also a statistically-non-significant finding. But, it is undoubtedly clinically-significant, and so we have presented it as clearly and transparently as possible.

21. *Similarly, the observed variability in QTc intervals sounds worrying the way it is described in the current text and was immediately picked up by reviewer 4. It is most unfortunate that the investigators only measured the QTc in the patients receiving DP. Without a control it is not possible to infer any causality.*

RESPONSE: We have updated the Results text to read: “… of children, with a maximum QTcF of 479msec. There was 1 (0.7%) case of a prolongation of >50 msec QTcF compared to baseline to 52 msec (Table 4, Table S6). Mean (SD) QTcF changes from baseline peaked at 17 (13) msec at 6 months and then declined to 8 (16) msec after 12 months of administration” in order to lend some balance to the dire-appearing QTcF measurements. In this case, children served as their own controls by measuring change from baseline, because we are not aware of data indicating that QTcF are prolonged by SP-AQ or Proguanil.

22. *At a minimum the authors have to show the QTc variability over the follow-up period for the 12 trial participants on whom they have serial QTcs. Leaving the safety reporting as it is now will create a lot of unnecessary concern*.

RESPONSE: The QTcF summary statistics are reported in **Table S8**, including the mean and standard deviation. However, we have now also included a figure of the QTcF over time with 95% confidence intervals at each timepoint collected (**Supplemental Figure**).

**Reviewer #1**

23. *Abstract: "incidence rates were similar" - I would recommend changing this to "not significantly different" as the confidence intervals allow for fairly large differences in the IRR.*

RESPONSE: Have changed x2 “were similar” to “were not significantly different”

24. *Abstract (and throughout results): 'p<0.01' - I would recommend reporting p-values more precisely unless <0.0001.*

RESPONSE: See #5 above.

25. *Abstract 'Serious adverse events were common but distributed between groups' - could this be more precisely written that 'distributed between groups', e.g. 'but no arm showed safety concerns'?*

RESPONSE: As with #20 above, the observed deaths among SP-AQ recipients, while not statistically significant, merits consideration if it is a potential alternative to Proguanil. We’ve altered this sentence to read: “Serious adverse events were common and distributed between groups, though more children died receiving monthly SP-AQ (n=7) than Proguanil (2) or DP (1).”

26. *Abstract: I note the clinicaltrials.gov page has many secondary endpoints that are not mentioned in the abstract. I would recommend (if the abstract reports secondary endpoints) it is clear there were other non-reported secondary endpoints that were not significant.*

RESPONSE: Clinicaltrials.gov enumerates 16 secondary outcomes, and Table 3 “Secondary outcomes in the as-treated population” includes 12 of these. The 4 not presented are molecular markers, because this is a lagging endpoint that is not yet generated, and 3 composite outcomes that were not ultimately included in the Statistical Analysis Plan (SAP) owing to their infeasiblity. The SAP tabulates the finalized outcomes.

27. *Methods, randomisation: was a fixed block allocation used, or was this stratified or using random block sizes? Providing the block size would be useful.*

RESPONSE: We have added to the text: “…Randomization was performed through the electronic data capture program using a block allocation with a block size of 9. No stratification criteria were used.”

28. *Methods, outcome measures: I would recommend each outcome on the clinicaltrials.gov page is mentioned here and reported in the paper, or a reason for it/them not being reported given here.*

RESPONSE: As noted above in #26, we have included the SAP with the submission which includes the final outcomes. The non-included outcomes were excluded following statistical consultation that essentially observed that what the PI aspired to do was numerically nonsensical.

29. *Methods, statistical analysis: "assuming that each experimental arm would be made" - I would change this to 'each comparison between experimental arm and control would be tested at the 0.0269 level (Dunnett correction)'.*

RESPONSE: We have changed this text to: “…assuming that each comparison between experimental arm and control would be tested at the 0.0269 level (Dunnett’s correction) in order to preserve a nominal alpha level of 0.05.”

30. *Methods, statistical analysis: I did not quite follow how the as-treated analysis worked. Was the indicator for SP-AQ arm a time varying covariate in the model? Later on it is said the AT population was not evaluated - is this just for secondary outcomes?*

RESPONSE: The original planned analysis was intention-to-treat (ITT); however, it was changed to as-treated (AT) to allow subjects in the SP-AQ group to crossover to the Proguanil arm amid DSMB safety concerns for 7 months (see #35 below). This allowed us to compute the incidence rate ratio (IRR) based on the exact duration within each treatment arm. Secondary dichotomous outcomes within the AT population were not evaluated for cumulative incidence rates due to multiple crossing of treatment arms. We have clarified this within the Statistical Analysis section: “The AT population was not evaluated for comparing cumulative incidence rates due to multiple crossing of treatment arms with events possibly occurring in each crossed arm.”

31. *Methods, statistical analysis: I note the protocol said different count outcome analyses would be considered with the one with the lowest residual variance used - this may be worth briefly mentioning in the analysis methods.*

RESPONSE: We have added to the Statistical Analysis section: “Model distributions considered included the NB, Poisson and zero-inflated Poisson and NB with the distribution chosen based on the residual plots.”

32. *Results, lines 238-242: similar comment to comment [23].*

RESPONSE: Have changed x2 “were similar” to “were not significantly different.”

**Reviewer #2**

33. *The tables need to be revised, they should include the number of each type of event, and the amount of person time.*

RESPONSE: We have added the variables to the associated tables (**Tables 2, 3 and S2**)

34. *And there is a problem with the analysis approach in that the conclusions relate to a comparison of the two monthly regimens, SPAQ and DP, but the statistical analyses are limited to comparison of each with the proguanil group.*

RESPONSE: The conclusions relate to the comparison of each of the two monthly regimens, SPAQ and DP to Proguanil (standard-of care). The purpose was determine if either of the experimental arms were more efficacious than the current SOC while also showing no additional safety concerns and not to determine if SPAQ or DP were statistically different.

35. *The DSMB stopped the SPAQ arm temporarily, patients in that arm of the trial were switched to proguanil, and then switched back to SPAQ later, I couldn't see any explanation of the reason for this.*

RESPONSE: We have added to the Results: “…the DSMB temporarily suspended administration of SP-AQ owing to the observation that the first 4 participant deaths occurred among children receiving SP-AQ. Pending the implementation of enhanced safety monitoring, addition of eligibility criteria, and adoption of a stopping rule, the SP-AQ group temporarily crossed over to Proguanil; SP-AQ dispensing resumed in May 2019.”

36. *It will be important to explain clearly how much person time was spent on each treatment and the number of doses received of each type of treatment, I didn't see these details.*

RESPONSE: We have added the person-years of follow-up for each treatment group and analysis population (see #33 above). The dosing schedule and duration of treatment in provided in the Supplemental document including table information is provided in **Table S1**.

37. *I felt that the conclusions were not supported by the data. The fact that the analyses didn't directly compare the SPAQ and DP groups may possibly have led to some confusion about how to interpret the results. For example, referring to SPAQ: "There was no signal of efficacy on parasitologic or hematologic outcomes" and "monthly DP did significantly reduce the incidence of asymptomatic parasitization... and we observed a significant reduction in dactylitis in the children receiving monthly DP". Such conclusions comparing SPAQ and DP are based on whether or not there was a statistically significant reduction (P<0.05) compared to proguanil, rather than a comparison of the magnitude of reduction between SPAQ and DP.
    A closed testing procedure could be have been used for example. (Using the closed testing procedure, first test the global hypothesis with a 2df level α test, and if it is rejected, then test each of pairwise comparison also at level α.)*

RESPONSE: Yes, we agree with the reviewer that a closed testing procedure could have been used if the purpose had been to determine if a treatment difference existed among the three treatment arms. However, our study was designed to test the null hypothesis of no treatment difference between each of the experimental arms (monthly SP-AQ and monthly DP) compared to the standard-of-care (SOC) arm (daily Proguanil) with an overall type I error of alpha=0.05. Each of the pairwise comparisons was performed using Dunnett’s ad-hoc method to compare the experimental arms to the SOC arm. The purpose of the study was not to determine if SP-AQ or monthly DP were better but rather to see if either were better than the current SOC. Our conclusion is clear in that we compare the benefit in dactylitis and asymptomatic *P. falciparum* infection using monthly DP compared to Proguanil, and that monthly SP-AQ had no measured benefit compared to Proguanil with increased morbidity.

38. *It seems unlikely there is any evidence of real differences between SPAQ and DP in this trial. For example, the rate ratio comparing the incidence of dactylis (blocked blood flow in the hands and feet causing painful swelling) in the DP group with the proguanil group was 0.47 (95%CI 0.23 - 0.96), and for the SPAQ group compared to proguanil, 0.78 (95%CI 0.41 - 1.50), it seems unlikely there could be a significant difference between these effects. And the rate ratios for asymptomatic malaria of 0.46 (0.17 - 1.20) and 0.21 (0.08 - 0.56), also look similar (the fact that the P-value is significant for DP and not for SPAQ does not indicate there is a difference in magnitude of effect).*

RESPONSE: The study was not powered to detect any differences between DP and SP-AQ. It does seem likely that efficacy on several outcomes is very similar. The clear difference is in safety.

39. *The conclusion that "Monthly SP-AQ does not appear to have a role as chemoprevention in children with sickle cell anemia" does not seem justified on the basis of these data. There were 10 deaths, 7 of them in the SPAQ arm. The hazard ratio comparing the SPAQ group with the proguanil group had a confidence interval from 0.92 to 32. It seems likely that the cause of death was linked to sickle cell disease rather than drug toxicity. Many millions of children, including many thousands with sickle cell disease, receive SPAQ each year in Seasonal Malaria Chemoprevention programmes. An earlier trial of SPAQ for malaria prevention in sickle cell disease (J Infect Dis. 2015 Aug 15;212(4):617-25) did not find any excess mortality -
    that trial, in Nigeria, had 3 arms with a similar number of patients per arm as the current study, there were 7 deaths in total, none of them in the SPAQ group.*

RESPONSE: The Olaosebikan trial [*J Infect Dis* 2015] had several important differences to ours:

a) We enrolled only children under 10y, while theirs included about 30% > 10y;
b) We administered SP-AQ monthly, while theirs was every other month;
c) We used a pre-qualified manufacturer (Guilin Pharmaceuticals) for SP-AQ, while theirs was procured locally and without any quality assurance, and therefore the actual content of their IP is unclear;
d) We confirmed HbSS by hemoglobin electrophoresis, and they did not. We note that , in our study, 11/333 (3%) of children screened did not have HbSS disease despite carrying this diagnosis clinically (Figure 1), and therefore it is not clear to us if their study truly included only children with HbSS.

In sum, without wishing to cast aspersions on that trial, our study had more rigorous confirmation both of enrollment criteria and of the investigational product.

More broadly, it is true that many millions of children receive annually monthly courses of SP-AQ as SMC, and for that reason these deaths were unexpected. It is also true that monitoring in those settings is necessarily minimal. As we note in the Discussion: “This high rate of SAEs was unexpected given the favorable safety profile of monthly SP-AQ in recipients as Seasonal Malaria Chemoprophylaxis,[33] during which 3-4 monthly courses are administered to all children under 5 years of age. This difference could be the result of our use of SP-AQ in a chronically ill population or accumulated risk following repeated monthly dosing.”

40. A more minor point related to the statistical analysis is the use of Fine & 203 Gray's method to allow death to be a competing risk, this is questionable. It is better to simply treat all competing events as though the individual were right censored at the time the competing event occurs. See [https://urldefense.com/v3/__https://statisticalhorizons.com/for-causal-analysis-of-competing-risks/__;!!OToaGQ!_E9MddCVJay2hIt11dKk7CtyJsv8ISaVmuJpn-VSW0u3CwY5czIxgepmBI2MuMY1l8o$](https://urldefense.com/v3/__https:/statisticalhorizons.com/for-causal-analysis-of-competing-risks/__;!!OToaGQ!_E9MddCVJay2hIt11dKk7CtyJsv8ISaVmuJpn-VSW0u3CwY5czIxgepmBI2MuMY1l8o$)

RESPONSE: We considered death a competing event when analyzing other clinical outcomes. Fine & Gray’s method allows us to analyze the sub-distribution hazard for each event to directly estimate the cumulative incidence of the respective outcome. Censoring at the time of the competing event will provide information regarding the etiology of the disease but may indicate that treatment is associated with death but not necessarily with the outcome of interest. If few deaths occur or the event rate is balanced, both methods will give approximately the same answer.

41. *The sample size section mentioned anticipated incidence rate of malaria of 3.7 episodes per person year but then says the study is powered to detect a reduction of 40% with DP and of 40% with proguanil, as if compared to placebo, but there was no placebo group.*

RESPONSE: That is true, there was no placebo. We added to the Supplemental Methods: “As described in the protocol (Section 9.5), the sample size was computed based upon the rate of observed events of clinical malaria among children aged up to 17m participating in a randomized trial of the RTS,S/AS01 malaria vaccine in Siaya district. The observed event rate was 3.7 episodes per year per child. This was considered a suitable estimate of the baseline event rate of the primary outcome amongst recipients of the standard of care daily Proguanil for several reasons:

1.	Malaria transmission is historically similar between Siaya and Homa Bay districts;

2.	The expected mean age of enrollment of children with SCA was expected to be 2-3 years of age;

3.	Because the vaccine efficacy was ultimately reported at 43%, and the observed rate represents an aggregate of vaccinated and unvaccinated children, this mild decrease from the true event rate could feasibly approximate the mild protection offered by daily Proguanil.”

**Reviewer #3**

42. *It would be useful to the readers if the authors mentioned the cause of the cross-over, which in this manuscript is implied as safety concern, but it is not clear.*

RESPONSE: See #35 above.

43. *Although there was no significant difference in the risk of clinical malaria comparing AS-AQ and DP to daily proguanil, I would suggest that the authors keep this as the lead finding and conclusion and then highlight a few positive related secondary outcomes e.g risk reduction for asymptomatic parasitemia.*

RESPONSE: We agree that the primary outcome should be a “lead finding” that is reported in the Abstract and as the focus of the first Results section and the first Results table (**Table 2**).

44. *In the results tables, it would be useful to clearly indicate numerator and denominator numbers used to estimate the incidence/risk, ie number of episodes of clinical malaria and total number of person years of follow-up for each chemoprevention arm.*

RESPONSE: See #33 above.

45. *Some of the numbers in the tables need to be revised to correct minor errors in rounding off, for example the proportion of households with IRS in table 1 is 26.4%, proportion of children reporting no prior painful crises in the last 12 months is 15.0%. The authors can check all numbers in the tables to ensure accuracy.*

RESPONSE: We’ve added denominators i.e. n/N to the categorical variables to clarify.

46. *Line 320, and 359 mention acceptability of DP but the methods of how this was assessed and the data to support this are missing.*

RESPONSE: We have added a call-out to **Table S1** that includes these data.

**Reviewer #4**

47. Provide more details on the study site "routine SCA clinic" from where participants were recruited and in addition, include details on the routine SCA care practices (including use of folic acid etc) at the site.

RESPONSE: We have added “… were enrolled from a routine SCA clinic at Homa Bay County Hospital and randomized…”. We have also added use of Folic acid at baseline to **Table 1.**

48. *Provide a rationale for performing certain investigations (e.g., repeat ECG) in only a selected subset of DP recipients (and why not across the three groups)*

RESPONSE: We are not aware of data reporting that Proguanil or SP-AQ prolong the QTc, or, at least, to any degree that limits their routine, repeated use as chemoprevention. This effect has been most limiting for monthly DP use, in children and in pregnant women. The open-label nature of our study allowed us to forego repeated ECG measurements in the non-DP groups, and the relevant comparison is then change from a person’s baseline in the QTc.

49. Per the manuscript (page 9) complete blood count was done every three months. It is surprising that authors present data for only hemoglobin, especially where other parameters (total WBC, platelets) are known to exhibit distinct characteristics in SCA and are linked to the pathophysiology of VOC/painful events as well as other significant SCA complications (e.g., stroke; transfusion requirements etc). Furthermore, the (historical) relationship between amodiaquine and neutropenia/agranulocytosis especially within the context of prophylaxis, is also well known. Given the above, I would strongly encourage authors to show the data for these other hematological parameters as this will strengthen the manuscript along both the safety and pathophysiology domains. In this respect, authors should present not only aggregated summaries but also a) changes from baseline; b) individual outlier data; as well as c) relationships (if any) between the data and the VOC/dactylitis outcome measure(s).

RESPONSE: We have added **Tables S4** and **S5** reporting the change in baseline overall and by study arm in the ITT population in neutrophils and in platelets. Although we did also measure WBCs as a part of the CBC, these values were uninterpretable owing, presumably, to the mis-counting of reticulocytes as leukocytes, and the uniformly elevated values make them unreliable. Therefore, they are presented nowhere. An analysis of the relationships between hematologic values and outcomes would indeed be interesting and is outside the scope of this analysis.

50. Authors should provide more complete information on participants in the "unanticipated crossover" group (number of participants, reasons for the intervention switch etc); suggest to (indeed required, per CONSORT Guidelines) to indicate such a 'flow' in the Trial Profile

RESPONSE: See #14 above re: the CONSORT checklist and #35 re: the cross-over.

51. *There is a discrepancy in the number of children in whom followup ECG was conducted (see pages 9 vs 16).*

RESPONSE: The Methods indicate “up to the first 20” allocated to DP who resided in Homa Bay town, but we ultimately only enrolled 12 meeting those criteria. The rationale for the criteria is that children participants came from far and wide, and we could not ensure adherence to the day-3 ECG return visit for participants from outside of the immediate area. To avoid confusion for further readers, we have removed “up to the first 20” from the Methods.

52. *The authors report more than 50% incidence of QTc prolongation in (an albeit limited) number of DP recipients. This is an important safety signal that should not, in my opinion, be ignored especially given the known relationship between DP and QTc interval prolongation. Importantly, the statement "given the observed safety of DP….."in the discussion (page 17) is not factually accurate.*

RESPONSE: See #21 above. Additionally, we think it is notable that other studies of DP monthly dosing are using 480msec as an event cutoff. Our interpretation is that the increases we noted are mild, and the measurements above 450msec reflect the higher-than-expected QTc intervals at baseline among these children.

53. *It is premature to conclude on the safety of DP for prophylaxis in SCA children given the findings and the discussion should be substantially revised to take this into consideration.*

RESPONSE: We have highlighted in response to #21 above the reassuring features of the QTc investigation, and complemented this in response to #19 above the potential population-level effects of DP use.

54. The summary statement that "our results provide a rationale for the wider use of DP with appropriate monitoring as a routine component of care for children with SCA in malaria endemic settings" is not supported by the data.

RESPONSE: We’ve softened this to “Our results provide a rationale to consider the wider use of DP with appropriate monitoring…”

55. Other comment: kindly use appropriate classification nomenclature for dactylitis (?hematologic event)

RESPONSE: We have used the definition in wide use for dactylitis in other studies, including the BABY HUG trial (Thornburg CD *et al. Blood* 2012; 120: 4304).

We are much obliged for your consideration of our manuscript.

Warm regards,

Steve M Taylor MD MPH

Associate Professor of Medicine and Global Health

Duke University
