# Supplementary material for: Monthly sulfadoxine/pyrimethamine-amodiaquine or dihydroartemisinin-piperaquine as malaria chemoprevention in young Kenyan children with sickle cell anemia: A randomized controlled trial
Source: PLoS Med. 2022 Oct 10;19(10):e1004104. doi: 10.1371/journal.pmed.1004104 (PMC9591057; doi:10.1371/journal.pmed.1004104)
Supplement: Attachment — Submitted filename: EPiTOMISE PlosMed RevReResponse.docx [file pmed.1004104.s006.docx]

20 June 2022

Richard Turner PhD

Senior Editor, *Plos Medicine*

Dr. Turner:

This letter accompanies the re-resubmission of our manuscript entitled “Monthly sulfadoxine/pyrimethamine-amodiaquine or dihydroartemisinin-piperaquine as malaria chemoprevention in young Kenyan children with sickle cell anemia: A randomized controlled trial.” We appreciate the opportunity to revise the manuscript again.

As with the initial submission, we have not submitted this manuscript for consideration to publish elsewhere and my co-authors Sarah Korwa, Angie Wu, Cynthia Green, Betsy Freedman, Sheila Clapp, Joseph Kipkoech Kirui, Wendy O’Meara, and Festus Njuguna all meet criteria for authorship and agree with this submission.

Below we have enumerated our point-by-point responses to the reviewer comments. For each comment, the reviewer comments are italicized, and our responses are indented and in normal text, with specific text changes underlined.

**Editorial Requests**

1. *As mentioned previously, please adapt the data statement (submission form) so as to comply with PLOS' data policy*

RESPONSE: We have created a functional email address [epitomise-study@duke.edu](mailto:epitomise-study@duke.edu) to which a collection of study-associated authors and non-authors will receive any inquiry.

2. *Noting the comments from one referee, please report additional measures as appropriate to their inclusion in the study protocol/SAP (i.e., identifying comparisons as post-hoc if needed).*

RESPONSE: We have added to the Methods: “The **Statistical Analysis Plan** describes all comparisons of the monthly groups to the daily Proguanil group; analyses comparing monthly DP to monthly SP-AQ were added later.”

3. *Please add a few words to the abstract to identify the trial site.*

RESPONSE: We’ve added to the Abstract “…between January 23, 2018, and December 15, 2020, in Homa Bay, Kenya.”

4. *Please correct the referencing at lines 383-4.*

RESPONSE: Sorry, fixed that.

5. *Throughout the text, please move reference call-outs before punctuation (e.g., "... occlusive complications [6,7]."; noting the absence of spaces within the square brackets).*

RESPONSE: We’ve changed these in the text file and supplement.

6. *Noting reference 29 and others, please use the journal name abbreviation "PLoS ONE".*

RESPONSE: Fixed those.

7. *Please correct the spelling of the first author's name for reference 36.*

RESPONSE: Fixed that.

8. *We did not find a completed CONSORT checklist with your revision and ask that you include this with your next revision. In the checklist, please refer to individual items by section (e.g., 'Methods') and paragraph number, not by line or page numbers as these generally change in the event of publication.*

RESPONSE: Whoops! I had drafted it but failed to include it. Included now, hopefully.

**Academic Editor comments**

9. *It may be most efficient to ask the authors to provide an additional direct comparison of*

*a) Text: Incidence of Pf infections in the DP vs SP-AQ arm*

*b) Text: Incidence of dactylitis in the DP vs SP-AQ arm*

*c)* *Table 1 and 2: add direct comparisons of DP vs SP-AQ arm*

*That should address “comparisons throughout the paper”.*

RESPONSE: We’ve added a new Table S2 with comparisons of the primary and secondary outcomes computed between DP and SP-AQ. We’ve also added the following text to the Results section:

- “Similar malaria rates were observed in the ITT population (**Table 2**), and when comparing DP to SP-AQ recipients (**Table S2**).”
- “Similar rates and differences were observed in the ITT population, and when comparing DP to SP-AQ recipients.”
- “Compared to DP recipients, SP-AQ recipients had similar incidences of asymptomatic *P. falciparum* parasitization (IRR 2.23; 95% CI: 0.68 – 7.29) and dactylitis (IRR 1.66; 95% CI: 0.77 – 3.56) (**Table S2**).”
- “Similarly, relative to DP recipients, the risk of death was elevated in SP-AQ recipients (HR 8.86; 95% CI: 0.74 – 106.55) (**Table S2**).”

10. *The authors should review their conclusions (abstract, summary, and discussion) if warranted by the findings.*

RESPONSE: We have done so thoroughly.

**Reviewer 1**

11. *I would still recommend briefly noting the deviation from which endpoints were specified in the SAP compared to ct.gov (perhaps using more diplomatic language than in the response to comment 28!) but will leave that as a suggestion to the editor.*

RESPONSE: To be clear, the non “diplomatic language” was directed against myself, and not any other party. Deviations from SAP addressed in #2 above.

**Reviewer 2**

12. *I think the presentation of results is misleading in the sense that the two interventions are being compared based on comparing the P-values for the difference of each with control, for example: "The incidence of P. falciparum infection relative to daily Proguanil was similar in the monthly SP-AQ group (IRR 0.46; 95% CI: 0.17—1.20; p=0.13) but reduced with monthly DP (IRR 0.21; 9 5% CI: 0.08—0.56; p=0.002)." But it seems unlikely that the efficacy differs between the two interventions. Similarly, the rate ratio comparing the incidence of dactylis (blocked blood flow in the hands and feet causing painful swelling) in the DP group with the proguanil group was 0.47 (95%CI 0.23 - 0.96), and for the SPAQ group compared to proguanil, 0.78 (95%CI 0.41 - 1.50), it seems unlikely there could be a statistically significant difference between these effects. The difference in mortality is also likely to be a chance finding. There needs to be a head-on comparison of the two groups. This applies to comparisons throughout the paper.*

RESPONSE: See response to #9 above.

**Reviewer 3**

13. *The primary outcome could also lead the concluding parts of the abstract and the discussion. The authors can first highlight lack of difference in the primary outcome, probably mention the reason why, and then go on to mention important significant secondary outcomes like parasite prevalence, like in the statement below. "Although there was no significant difference in the incidence of clinical malaria between the three treatment arms, possibly due to reduced malaria transmission intensity, DP was associated with……….then go on to mention important secondary outcomes that were significant."*

RESPONSE: We agree that the primary outcome should be highlighted, though there is risk of mis-interpretation of asserting strongly that there was no difference in the primary outcome, because the limited transmission rendered the study incapable of definitively proving this. To balance this, the 1^st^ paragraph in the Discussion includes as the initial summary of findings: “Consistent with enhanced community malaria control which sharply reduced transmission, we recorded very few episodes of malaria. Although this prevented demonstration of the superiority of the monthly regimens to prevent malaria, monthly DP did significantly reduce the incidence of asymptomatic parasitization.”

14. *Line 371, I suggest that "acceptability" be removed from the statement. To me, data presented in supplemental table 1 supports adherence not acceptability.*

RESPONSE: The acceptability claim is based on the variable in Table S1 “Ever difficulties getting the child to take the medication”. Because only 6.3% of parents answered for DP that they ever had difficulty getting their child to take DP (compared to 27.5% for SP-AQ), we interpret this as DP being acceptable to the children.

Thanks again for your consideration of our manuscript.

Warm regards,

Steve M Taylor MD MPH

Associate Professor of Medicine and Global Health

Duke University
