# Supplementary material for: Monthly sulfadoxine/pyrimethamine-amodiaquine or dihydroartemisinin-piperaquine as malaria chemoprevention in young Kenyan children with sickle cell anemia: A randomized controlled trial
Source: PLoS Med. 2022 Oct 10;19(10):e1004104. doi: 10.1371/journal.pmed.1004104 (PMC9591057; doi:10.1371/journal.pmed.1004104)
Supplement: Attachment — Submitted filename: EPiTOMISE PlosMed 3rdResubResponse.docx [file pmed.1004104.s007.docx]

24 August 2022

Caitlyn Moyer PhD

Associate Editor, *Plos Medicine*

Dr. Moyer:

This letter accompanies the re-resubmission of our manuscript entitled “Monthly sulfadoxine/pyrimethamine-amodiaquine or dihydroartemisinin-piperaquine as malaria chemoprevention in young Kenyan children with sickle cell anemia: A randomized controlled trial.”

As with the initial submission, we have not submitted this manuscript for consideration to publish elsewhere and my co-authors Sarah Korwa, Angie Wu, Cynthia Green, Betsy Freedman, Sheila Clapp, Joseph Kipkoech Kirui, Wendy O’Meara, and Festus Njuguna all meet criteria for authorship and agree with this submission.

Below we have enumerated our point-by-point responses to the requested changes. For each comment, our responses are indented and in normal text, with specific text changes underlined.

**Editorial Requests**

1. Data availability statement: The Data Availability Statement (DAS) requires revision. Please spell out “ICF” in the statement rather than using an acronym. It would be helpful to include a general web address where interested researchers can learn more about the dataset/ accessing the data. Please note that a study author cannot be the contact person for the data.

RESPONSE: The DAS has been revised. There is no general web address. The contact is the functional email address as specified.

2. Funding statement: Please add the following information to the funding statement: “SP-AQ was supplied free of charge to the trial by Guilin Pharmaceuticals, which had no role in the design, conduct, analysis, reporting, or decision to report the results.”

RESPONSE: We’ve added this.

3. Throughout: Please be sure that all abbreviations and acronyms are defined in the text at their first point of use (both for the Abstract and the main text).

RESPONSE: We have tried to do so.

4. Abstract: Background: We suggest an additional sentence providing context on the current chemoprevention practices or similar.

RESPONSE: Changed to: “Chemoprevention regimens vary between countries, and the comparative efficacy of prevention regimens is largely unknown.”

5. Abstract: Line 40: Please define HbSS at first use in the text. Please describe some brief information on the inclusion criteria/recruitment of the children with HbSS. Please note in the abstract that children were followed up monthly for 12 months.

RESPONSE: Changed to “We enrolled Kenyan children aged 1-10 years with homozygous hemoglobin S (HbSS) in a…” and “…or monthly dihydroartemisinin/piperaquine (DP) and followed monthly for 12 months”

6. Abstract: Line 45: Please provide a brief description of painful events and how this outcome was determined. Please list other secondary outcomes, including: parasitologic outcomes and hematologic outcomes.

RESPONSE: Added: “…the cumulative incidence of painful events by self-report.” Also added, because there are too many secondary outcomes to make for interesting reading: “Secondary outcomes included other parasitologic, hematologic, and general events.”

7. Abstract: Line 58-59: Please point out that the differences in numbers of deaths between groups did not reach statistical significance.

RESPONSE: We changed this to add the actual modeled hazard ratios: “Serious adverse events were common and distributed between groups, though compared to daily Proguanil (n=2), more children died receiving monthly SP-AQ (n=7; Hazard Ratio [HR] 5.44; 95% CI: 0.92—32.11) but not DP (n=1; HR 0.61; 95% CI 0.04—9.22).”

8. Abstract: Conclusions: We suggest revising the first sentence to address the study implications without overreaching what can be concluded from the data, commenting on the primary outcome first and then key secondary findings; the phrase "In this study, we observed ..." may be useful. We suggest: In this study, we observed that monthly malaria chemoprevention with monthly SP-AQ or monthly DP did not significantly reduce incidence of clinical malaria compared with daily Proguanil. Monthly DP was associated with a lower rate of dactylitis and infection with P. falciparum relative to the Proguanil group, despite limited malaria transmission during the study period.” Please note that there is usually a distinction in the language in terms of causal vs. associational for primary and secondary trial outcomes. It would be beneficial to use associational language throughout when describing secondary outcomes.

RESPONSE: We changed this to “In this study with limited malaria transmission, malaria chemoprevention in Kenyan children with sickle cell anemia with monthly SP-AQ or DP did not reduce clinical malaria, but DP was associated with reduced dactylitis and *P. falciparum* parasitization.”

We understand the general practice of favoring associational language for secondary outcomes. However, in this specific case of the secondary outcome being infection with malaria parasites following administration of antimalarial medications, we feel that causal language is justified. We do agree with the use of associational language for secondary outcomes for which there is a more convoluted causal pathway between the exposure and the outcome, e.g. dactylitis.

9. Author summary: Line 93-94: Please rephrase slightly to: “...a higher rate of deaths occurred in the SP-AQ group, but this did not reach statistical significance.” or similar.

RESPONSE: Changed to: “…but SP-AQ was associated with an unexpectedly higher rate of deaths that did not achieve statistical significance”

10. Author summary: Line 98-99: Please revise to: “The observed greater number of deaths among children with SCA in the SP-AQ treated group was unexpected, and while this association did not reach statistical significance, further evaluation is warranted.” or similar.

RESPONSE: Changed to: “SP-AQ-associated mortality among children with SCA was unexpected and though it did not achieve statistical significance merits further evaluation”

11. Methods: Line 145-146: Please revise to: “"This study is reported as per the Consolidated Standards of Reporting Trials (CONSORT) guideline (S1 Checklist)."

RESPONSE: Added that.

12. Methods: Please provide additional details on eligibility in the main text of the Methods.

RESPONSE: Added per #32 below.

13. Methods: Lines 196 and 209: Please make sure that a complete listing of all primary and secondary outcomes, and safety outcomes, is included here.

RESPONSE: We’ve highlighted major ones here, with a direct reference to the Supplemental Information which has the fuller listing with their strict definitions as well. We have also included the full protocol which lists them as well. There are so many that it would be unwieldy to list them all in the main text, particularly given that several require a definition to make sense. For these reasons, we have provided an executive summary in the main text and the fuller listings in the additional documents.

14. Methods: Line 223-229: Please provide an explanation for the cross-over from SP-AQ to Proguanil.

RESPONSE: Added: “this crossover resulted from the temporary suspension of SP-AQ administration at the request of the DSMB to allow assessment of 4 deaths among SP-AQ recipients.”

15. Methods: Line 242-244: Please describe changes to the protocol, e.g. those prompted by the pandemic, in the text. Please mention and explain the reasons for the modifications including: exclusion of children with low hemoglobin, the collection of baseline laboratory testing at screening rather than enrollment, and the enhanced monthly hemoglobin monitoring, SAE review, and patient referral. Please complete and include the CONSERVE checklist, if relevant (https://urldefense.com/v3/__https://www.equator-network.org/reporting-guidelines/guidelines-for-reporting-trial-protocols-and-completed-trials-modified-due-to-the-covid-19-pandemic-and-other-extenuating-circumstances-the-conserve-2021-statement/__;!!OToaGQ!sS3byGJasa0FLFaWQgFVaXsmL5pxuYSaFNcKFzW9m8452tXLR4SFDGjPaYC4V1m1bTts2SeOQUTOWR-AZ7be$ )

RESPONSE: Luckily, our enrollment was completed and follow-ups were winding down during Covid, which limited changes. We have altered the sentence to “Pre-specified sub-group analyses explored the impact of changes to protocol and access to care prompted by the Covid-19 pandemic and other covariates on clinical malaria and painful events by including an interaction term in the model; the main pandemic-related protocol change was that monthly follow-up visits alternated between in-person and telephone visits.”

16. Results: Line 302: Please revise to: “...DP was associated with reduced rates of asymptomatic parasitization…”

RESPONSE: See #8 above.

17. Results: Line 315: Please revise to: “...did not modify the association between DP and dactylitis…”

RESPONSE: Changed to “These subgroups also did not modify the association of DP with reduced rates of dactylitis, which was observed…”

18. Results: Please report p values in the text throughout, where appropriate, for example at lines 318-333.

RESPONSE: In this section, p-values have been provided in the text for all statistical comparisons done per the protocol that presented hazard or incidence rate ratios. Items presented with event rates and cumulative incidence estimates (e.g., SAEs) were not directly compared but presented with 95% confidence intervals.

19. Results: Lines 329-333: Please revise to indicate that the observed increase in risk of death among SP-AQ recipients (compared with the Proguanil or with the DP treated groups) was not statistically significant.

RESPONSE: Changed to: “…SP-AQ recipients (HR 8.86; 95% CI: 0.74 – 106.55), though this did not achieve statistical significance.”

20. Discussion: Line 357-358: Please revise to: “...monthly DP was associated with significantly lower incidence of asymptomatic parasitization.”

RESPONSE: See #8 above.

21. Discussion: Line 365: Please revise to: “Monthly DP was associated with a reduced rate of episodes of dactylitis compared to daily Proguanil…”

RESPONSE: Changed to: “Monthly DP was associated with a reduced rate of episodes of dactylitis compared to…”

22. Discussion: Line 398-399: It is not clear that the data support this statement, and we suggest removing it.

RESPONSE: We explain the reasons for this summary in the balance of the paragraph. It is hard to imagine concluding that a medication has any role for chemoprevention in children with sickle cell anemia when it demonstrates no measurable benefit and only serious, irreparable harm.

23. Discussion: line 426-427: Please mention the primary study outcome up front in the Conclusion paragraph. Please revise the sentence to: “...monthly DP was observed to be acceptable and was associated with reduced incidence of dactylitis and asymptomatic P. falciparum infection…”

RESPONSE: We have foregrounded the primary outcome with: “As a result, neither monthly SP-AQ nor DP reduced the incidence of the primary outcome clinical malaria, though monthly DP did…”

24. Discussion: Line 429: We suggest removing: “and was associated with unacceptable morbidity.”

RESPONSE: We have changed this to reinforce the language elsewhere in the text: “Monthly SP-AQ had no measurable benefit compared to daily Proguanil, and was associated with an unexpectedly higher mortality rate.”

25. Figure 1: Please define all abbreviations used in the legend.

RESPONSE: Changed to “Bars labeled IRS indicate the months in which Indoor Residual Spraying…”

26. Figure 2: Please define all abbreviations used in the legend. On the axis, please make it clear that the point estimates are relative for each SP-AQ or DP relative to Proguanil, not relative to each other.

RESPONSE: We have changed the column headings to clarify the comparisons in each column.

27. Table 1: It seems as though p values should not be needed as participants were randomized.

RESPONSE: There are indeed no p-values present in Table 1.

28. Table 3: Where reporting the p value for asymptomatic P. falciparum infection, please do not report as p<0.01. Please report the exact p value, unless p<0.001.

RESPONSE: We have updated to “0.002.”

29. Reference 29, 34, and 36: Please change the journal to PLoS One.

RESPONSE: Changed.

30. CONSORT checklist: Thank you for including the CONSORT checklist. On the checklist, please make it clear you are referring to paragraph numbers within sections. For “Protocol” please report the location as Supporting Information files, or similar. For “Funding” please report the location as Funding statement, or similar.

RESPONSE: We have updated the checklist as requested and to reflect the changes in paragraphs resulting from these other changes herein.

31. Study Protocol: Thank you for including a copy of the study protocol. Please confirm that the image used in Appendix A-E is not reproduced from other sources that are not CC-BY. Please see https://urldefense.com/v3/__https://journals.plos.org/plosmedicine/s/figures*loc-licenses-and-copyright__;Iw!!OToaGQ!sS3byGJasa0FLFaWQgFVaXsmL5pxuYSaFNcKFzW9m8452tXLR4SFDGjPaYC4V1m1bTts2SeOQUTOWVmHjYCN$ for more information.

RESPONSE: I don’t know its origin. We defer to editorial authority here for possible deletion.

32. Supplemental Methods: Please move the description of inclusion/exclusion criteria and sample size considerations to the main text.

RESPONSE: We moved the inclusion/exclusion criteria to the main text and removed it from the supplement.

We have left the sample size text as-is, because the main text includes the numerical, referenced support for the sample size, and the supplement includes only additional justifications. We have re-labeled the subheading in the supplement to “Additional sample size considerations.”

33. Supplemental Figure: Please define all abbreviations used in a descriptive legend.

RESPONSE: We have now.

34. Supporting information Table S6 and S7: Please define “-” and “(-,-)” in the legend.

RESPONSE: We have now.

35. Supporting information: Please check reference list formatting. Please use the "Vancouver" style for reference formatting, and see our website for other reference guidelines https://urldefense.com/v3/__https://journals.plos.org/plosmedicine/s/submission-guidelines*loc-references__;Iw!!OToaGQ!sS3byGJasa0FLFaWQgFVaXsmL5pxuYSaFNcKFzW9m8452tXLR4SFDGjPaYC4V1m1bTts2SeOQUTOWc-V8SaH$

RESPONSE: We think we fixed these.

Thanks again for your consideration of our manuscript.

Warm regards,

Steve M Taylor MD MPH

Associate Professor of Medicine and Global Health

Duke University
